# Mega-Analysis of Structural Brain Imaging in Functional Neurological Disorder

**DOI:** 10.1016/j.biopsych.2026.06.031

**Published:** 2026-07-10

**Authors:** Matt Butler, Miriam Vignando, Jane B. Allendorfer, Thomas Hassa, Silvina G. Horovitz, Jacobus F.A. Jansen, Johannes Jungilligens, Wesley T. Kerr, Ramesh S. Marapin, Enrico Premi, Aleksandar J. Ristic, Tereza Serranova, Petr Sojka, Daichi Sone, Barbara Borroni, Anthony David, Quinton Deeley, Ibai Diez, Sara Paredes Echeverri, Dawn S. Elashiv, Jeannette M. Gelauff, Mark Hallett, Richard Kanaan, Marina A.J. de Koning-Tijssen, Madeleine Love, Mauro Magoni, Kirpal Sadheura, Noriko Salamon, Noriko Sato, Ariel Schoenfeld, John M. Stern, Jerzy P. Szaflarski, David L. Perez, Selma Aybek, Mitul A. Mehta, Timothy Nicholson

**Affiliations:** Institute of Psychiatry, Psychology and Neuroscience, King’s College London, London, United Kingdom; Institute of Psychiatry, Psychology and Neuroscience, King’s College London, London, United Kingdom; University-Alabama Birmingham Epilepsy Center, Department of Neurology, Heersink School of Medicine, University of Alabama, Birmingham, Alabama; Lurija Institute for Rehabilitation and Health Sciences, Stuttgart, Germany; National Institute of Neurological Disorders and Stroke, National Institutes of Health, Bethesda, Maryland; Department of Radiology and Nuclear Medicine, Maastricht University Medical Center, Maastricht, the Netherlands; Department of Neurology, University Hospital Knappschaftskrankenhaus Bochum, Ruhr University Bochum, Bochum, Germany; Department of Neurology and Psychiatry and Biobehavioral Sciences, University of California, Los Angeles, Los Angeles, California; Department of Neurology and Biomedical Informatics, University of Pittsburgh, Pittsburgh, Pennsylvania; University Medical Center Groningen, Groningen, the Netherlands; Stroke Unit, Azienda Socio Sanitaria Territoriale Spedali Civili, Brescia, Italy; School of Medicine, University of Belgrade, Belgrade, Serbia; Charles University, Prague, Czech Republic; Charles University, Prague, Czech Republic; National Center of Neurology and Psychiatry, Tokyo, Japan; Department of Clinical and Experimental Sciences, University of Brescia, Brescia, Italy; Molecular Markers Laboratory, Istituto di Ricovero e Cura a Carattere Scientifico Istituto Centro San Giovanni di Dio, Fatebenefratelli Brescia, Italy; University College London, London, United Kingdom; Institute of Psychiatry, Psychology and Neuroscience, King’s College London, London, United Kingdom; Mass General Brigham Departments of Neurology and Psychiatry, Massachusetts General Hospital and Brigham and Women’s Hospital, Harvard Medical School, Boston, Massachusetts; Mass General Brigham Departments of Neurology and Psychiatry, Massachusetts General Hospital and Brigham and Women’s Hospital, Harvard Medical School, Boston, Massachusetts; Department of Neurology and Psychiatry and Biobehavioral Sciences, University of California, Los Angeles, Los Angeles, California; University Medical Center Groningen, Groningen, the Netherlands; National Institute of Neurological Disorders and Stroke, National Institutes of Health, Bethesda, Maryland; University of Melbourne, Melbourne, Victoria, Australia; University Medical Center Groningen, Groningen, the Netherlands; Institute of Psychiatry, Psychology and Neuroscience, King’s College London, London, United Kingdom; Stroke Unit, Azienda Socio Sanitaria Territoriale Spedali Civili, Brescia, Italy; Berkshire Healthcare NHS Foundation Trust, Berkshire, United Kingdom; Department of Neurology and Psychiatry and Biobehavioral Sciences, University of California, Los Angeles, Los Angeles, California; National Center of Neurology and Psychiatry, Tokyo, Japan; Lurija Institute for Rehabilitation and Health Sciences, Stuttgart, Germany; Department of Neurology and Psychiatry and Biobehavioral Sciences, University of California, Los Angeles, Los Angeles, California; University-Alabama Birmingham Epilepsy Center, Department of Neurology, Heersink School of Medicine, University of Alabama, Birmingham, Alabama; Mass General Brigham Departments of Neurology and Psychiatry, Massachusetts General Hospital and Brigham and Women’s Hospital, Harvard Medical School, Boston, Massachusetts; Neurology, Faculty of Sciences and Medicine, Fribourg University, Fribourg, Switzerland; Institute of Psychiatry, Psychology and Neuroscience, King’s College London, London, United Kingdom; Institute of Psychiatry, Psychology and Neuroscience, King’s College London, London, United Kingdom

## Abstract

**BACKGROUND:**

Despite recent advances, the pathophysiology of functional neurological disorder (FND) remains incompletely understood. Structural neuroimaging studies have identified gray matter alterations in somatomotor-, salience-, limbic-, and default mode network–associated areas, although findings have been inconsistent. Mega-analyses, which combine individual-level data across studies, can help clarify structural alterations.

**METHODS:**

We conducted a mega-analysis of brain structural morphometrics derived from T1-weighted magnetic resonance imaging scans from 15 international research groups. After across-site harmonization with ComBat, we compared 493 patients with functional motor disorder and functional seizures with 564 healthy control participants. Euler numbers were included to account for head motion.

**RESULTS:**

The FND cohort showed reduced cortical thickness in the bilateral superior frontal gyri (left *d* = 0.22, right *d* = 0.21) and sulci (*d* = 0.22 and 0.23), bilateral superior precentral sulcus (*d* = 0.22 and 0.26), right precentral gyrus (*d* = 0.25), right paracentral gyrus and sulcus (*d* = 0.23), right cuneus (*d* = 0.23), and right inferior opercular gyrus (*d* = 0.21); we also found reduced left postcentral gyrus surface area (*d* = 0.25) and right hippocampal volume (*d* = 0.22). No regions were different in relative surface area. There were no associations between morphometrics and illness duration or lifetime history of depression or anxiety. Differences between motor and seizure variants were not identified.

**CONCLUSIONS:**

This large mega-analysis suggests subtle morphometric differences, particularly in prefrontal and motor regions. This may represent predisposing vulnerabilities, compensatory mechanisms, or FND-specific alterations. Improved neuropsychiatric characterization of FND research cohorts will help further contextualize the biological relevance of structural alterations.

Functional neurological disorder (FND), previously known as conversion disorder, is a common, costly, and potentially disabling neuropsychiatric disorder (1–3). It is characterized by neurological symptoms involving disturbances in voluntary control of movement or alterations in awareness (4,5). The 2 most prevalent subtypes of FND include functional motor disorder (FND-motor) and functional seizures (FND-seiz) (4). Individuals often experience more than one symptom type simultaneously or over time (6).

FND is one of the most common reasons for individuals presenting to outpatient neurology and neuropsychiatry clinics (5,7), and it causes equitable disability to multiple sclerosis, Parkinson’s disease, and epilepsy (8,9). Despite available treatments, many patients experience long-term disability (10). There is a pressing need to better understand the neurobiology of FND to aid the development of novel mechanistically informed interventions (11).

Form follows function: the brain’s structure and function are intricately linked, and changes in activity and functional connectivity may be associated with morphological alterations (12,13). Morphometric alterations have been reported in areas such as the frontal and limbic cortices, but the findings from single-center studies with small to moderate sample sizes have been heterogeneous (maximum *n* = 48) (14). Systematic reviews of structural neuroimaging have highlighted alterations in the prefrontal cortex (including paralimbic structures), motor regions, and limbic areas (14–16). Some studies have identified differences in morphometrics related to symptom severity, illness duration, and predisposing vulnerabilities (17–20).

Structural magnetic resonance imaging (MRI) studies in FND-motor have shown reduced volumes in subcortical structures such as the bilateral basal ganglia and right thalamus (21–23) and larger volumes in subcortical regions such as the left amygdala, striatum, and cerebellum and bilateral thalamus (24). Cortical studies in FND-motor have shown smaller volumes in the left sensorimotor cortices (24) and right post-central gyrus (23) and greater cortical thickness in the bilateral premotor cortex (17).

Structural neuroimaging studies of FND-seiz have shown smaller volumes in the middle frontal gyrus, anterior cingulate gyrus, and bilateral cerebellum (18) and smaller thickness in the frontal gyri, superior temporal, and limbic regions (18,25–28). There is also evidence of smaller subcortical volumes in the left amygdala and hippocampus (29) and reduced surface area in frontal and insular regions in FND-seiz (30).

Overall, the literature is heterogeneous, and some studies have shown limited or no differences (19,31–35). One possible reason is the high prevalence of mental health disorders in patients with FNDs (36), which only a subset of studies have accounted for (28). Other reasons may be the relatively small sample sizes and the potentially subtle differences expected to exist between patients with FND and control participants. Given this, big data approaches are necessary.

Large-scale analyses of shared data—for example via mega-analyses—help overcome heterogeneity observed in individual studies (37–39). Mega-analytic approaches to neuroimaging datasets have distinct advantages, including a consistent methodology, improved ability to address confounding variables, a uniformly applied analysis model, and the ability to include unpublished structural data (37). A further advantage is the improved power.

Cortical thickness and surface area are considered orthogonal components that are affected by distinct underlying processes; thus, they can be considered separate components. The primary correlate of cortical volume is cortical surface area, but volume loss is best captured by cortical thickness (40). Thus, separate analysis of these 2 components offers a better understanding of underlying cortical changes than the analysis of volume measures alone (28).

A comprehensive analysis of all available FND structural neuroimaging data is a critical next step in developing a more complete understanding of the underlying structural neurobiology (11). In this mega-analysis, we aimed to obtain data from research groups that had published any study involving a structural MRI sequence in patients with FND-motor and FND-seiz (including mixed cohorts) and to aggregate raw individual participant data into a single analysis.

We hypothesized that previously identified cortical and subcortical regions, such as the frontal, motor, (para)limbic, and basal ganglia, would be significantly different in patients with FND versus healthy control (HC) participants. We aimed to probe these general hypotheses using a whole-brain approach, extracting morphometric values from datasets, harmonizing the data to reduce intersite variation, and comparing regions via multivariate analysis of covariance (MANCOVA). Secondary analyses were also performed to contextualize the potential impact of FND-motor versus FND-seiz, lifetime history of depression or anxiety, and biological sex differences.

## METHODS AND MATERIALS

The study obtained King’s College London ethical approval from the Research Ethics Office, Psychiatry, Nursing and Midwifery Research Ethics Panel (LRS-20/21–22422) on April 15, 2021. Included studies were identified via an initial systematic literature search (see the Supplemental Methods).

### Data Requests

We contacted research groups for T1-weighted MRI scans as well as demographic and clinical information. In total, 26 authors were contacted, and 20 responded. Of these 20, 4 could not access data, 1 could not arrange a data sharing agreement, and 1 declined to participate. Further studies that met the inclusion criteria (*n* = 3) were identified via word of mouth (one additional site). We asked groups to send as much cohort characterization information as was available.

### Participants

Patients were included in the main FND group if they had any FND-seiz or FND-motor, as defined by the information given by the contributing groups (see the Supplemental Methods for more information).

### MRI Data Preprocessing and Harmonization

The analysis pipeline for preprocessing and data harmonization was based on that used in Vignando *et al.* (37). MRI data were preprocessed with FreeSurfer version 6.0.0 (41). Individual cortical thickness, cortical surface area, and subcortical volume measures were extracted based on the Destrieux atlas (number of regions entered in the principal component analysis [PCA]: *n* = 148 for thickness, *n* = 148 for surface area, *n* = 20 for subcortical volumes) (42). For a complete description of the analysis pipeline, see the Supplemental Methods.

### ComBat Harmonization

To minimize variance due to different scanners and maintain variance related to biological features, we used the ComBat harmonization method (an empirical Bayesian algorithm) on the parcellation results (43,44). This method has been previously used successfully in studies of cortical thickness, cortical surface area, and subcortical volumes (37,43) (see Supplemental Note S1 for more details).

### Motion Correction

Subtle in-scanner motion may produce artifactual alterations in cortical thickness (45); this may be particularly noteworthy in FND-motor (11). Euler numbers have been shown to be a reliable numerical estimate of image quality and motion (46). Euler numbers were calculated using the function in Free-Surfer, and a mean value was produced ([left + right]/2). We were unable to obtain a Euler number for 1 participant, who was not included in relevant analyses.

### Case-Control Matching

Prior to between-group analyses, case-control matching was performed via fuzzy matching in SPSS 24 (IBM Corp., 2016) with tolerance factors for age and sex set to 1 year and 0, respectively. Case-control matching was repeated separately for each of the subanalyses (FND vs. HC, FND-motor vs. HC, FND-seiz vs. HC, FND-motor vs. FND-seiz, FND sex-matched sample, lifetime depression^+^ vs. depression^-^, and lifetime anxiety^+^ vs. anxiety^-^).

### Feature Selection

To reduce dimensionality, we conducted a feature-selection PCA. This ensured computational tractability and interpretability for the MANCOVA via an unbiased, data-driven structure approach. We did not undertake feature selection for subcortical volumes due to a smaller number of regions (*n* = 20). The PCA feature selection was performed independently for each of the subanalyses (FND vs. HC, FND-motor vs. HC, FND-seiz vs. HC, FND-motor vs. FND-seiz, FND sex-matched sample, lifetime depression^+^ vs. depression^-^, and lifetime anxiety^+^ vs. anxiety^-^). For a complete description, see the Supplemental Methods.

### Group Difference Analyses

We used MANCOVAs to compare the FND group with the HC group, while controlling for age, sex, estimated total intracranial volume (eTIV) (subcortical), and Euler number (cortical thickness and surface area). Results were corrected for multiple comparisons using false discovery rate correction (see Supplemental Tables S2–S7 for all regions entered into the significant MANCOVAs). The primary analyses were patients with FND versus HC participants; there were several secondary analyses as outlined below.

The models were calculated using SPSS and R version 4.0.0 (R Core Team, 2017). Effect sizes (Cohen’s *d*) were derived from η_p_^2^ values. Figures were created by overlaying regional labels on a brain render with color coding based on effect size.

### Clinical Characteristics

We aimed to include as confounding variables any clinical information that was available for >60% of participants. Depression and anxiety caseness were established if the groups had indicated the presence of either current or lifetime depression or anxiety or via information derived from clinical scales (see the Supplemental Methods).

### Secondary Analyses

The analyses outlined above were repeated for each of the separate subanalyses (FND vs. HC, FND-motor vs. HC, FND-seiz vs. HC, FND-motor vs. FND-seiz, FND sex-matched sample, lifetime depression^+^ vs. depression^-^, and lifetime anxiety^+^ vs. anxiety^-^).

### Illness Duration

We ran a partial correlation analysis between illness duration in the significant regions in the FND group, while controlling for age and sex (and eTIV in the case of subcortical volumes).

### Leave-One-Site-Out Analysis

The MANCOVA for the overall FND cohort was repeated, with manual removal of 1 of the 15 study cohorts at a time to check by visual inspection whether a single site was driving the results. Because there was no indication of a single site effect, we did not calculate bias or variance estimators.

### Exploratory PCA

To interpret the results from the MANCOVAs further, we used an exploratory post hoc PCA to identify latent dimensions and determine how well variables fit into these dimensions. We aimed to explore how the regions that differ interact and contribute to variance in FND (37). For a complete description, see the Supplemental Methods.

## RESULTS

Fifteen groups contributed, offering published or unpublished data as NIfTI files or preprocessed datasets. We began processing with 553 FND and 594 HC MRI scans; 23 FND and 30 HC scans were discarded due to insufficient quality. Further clinical scans were removed due to missing clinical data (*n* = 24) or not meeting diagnostic criteria (*n* = 13). The final sample comprised 493 patients (372 female [75.5%]; mean [SD] age = 38.2 [12.7] years) and 564 HC participants (402 female [71.3%]; mean [SD] age = 38.1[12.7] years); (see Table 1). In total, there were 314 patients in the FND-motor group and 193 patients in the FND-seiz group (*n* = 14 in both groups).

### Gross Metrics

Mean eTIV was significantly different between groups (1,442,791 mm^3^ in patients with FND vs. 1,492,294 mm^3^ in HCs; *t*_1055_ = 5.325, *p* < .001). This difference remained significant (*p* < .001) when age and sex were included as covariates.

There was a significant difference between total surface area of patients with FND and HC participants (164,703 mm^2^ in patients with FND vs. 169,943 mm^2^ in HC participants; *t*_1055_ = 5.431, *p* < .001). This difference remained significant (*p* < .001) when age and sex were included as covariates.

There was a significant difference between mean Euler number in patients with FND and HC participants (-62.5 [55.1] vs. -54.4 [52.0], *t*_923_ = 2.31, *p* = .21). There was no significant difference between mean Euler values in patients with FND-motor and HC participants (-54.8 [53.4] vs. -54.4 [52.0], *t*_761_ = 0.109, *p* = .913). There was a difference between mean Euler values in patients with FND-seiz and HC participants (-74.8 [54.5] vs. -54.4 [52.0], *t*_636_ = 4.37, *p* <.001).

### Full FND Cohort

After case-control matching, 463 patients with FND (352 female [76.0%], mean age [SD] = 37.83 [12.35], including 294 patients with FND-motor [63.50%]), and 463 HC participants (352 female [76.03%], mean age = 37.83 [12.35]) were included in the analysis.

### Cortical Thickness

There was a significant main effect of FND (*F*_102,819_ = 1.659, *d* = 0.91, *p* < .001), age (*F*_102,819_ = 9.89, *d* = 2.22, *p* < .001), mean Euler number (*F*_102,819_ = 3.013, *d* = 1.22, *p* < .001), and sex (*F*_102,819_ = 3.301, *d* = 1.27, *p* = .001).

Pairwise comparisons of between-subjects effects revealed lower cortical thickness values in patients with FND compared with HC participants in 10 regions: the bilateral superior frontal gyrus (left *d* = 0.22, *p* = .013; right *d* = 0.21, *p* = .018), bilateral superior frontal sulcus (left *d* = 0.22, *p* = .013; right *d* = 0.23, *p* = .010), bilateral superior precentral sulcus (left *d* = 0.22, *p* = .013; right *d* = 0.26, *p* = .010), right precentral gyrus (*d* = 0.25, *p* = .010), right paracentral gyrus and sulcus (*d* = 0.23, *p* = .011), right cuneus gyrus (*d* = 0.23, *p* = .010), and right inferior opercular gyrus (*d* = 0.207, *p* = .018) (Figure 1).

### Surface Area (Relative)

A main effect was observed for FND (*F*_96,825_ = 1.306, *d* = 0.780, *p* = .032), age (*F*_96,825_ = 4.275, *d* = 1.410, *p* < .001), Euler number (*F*_96,825_ = 3.128, *d* = 1.208, *p* < .001), and sex (*F*_96,825_ = 3.709, *d* = 1.312, *p* < .001).

Pairwise comparisons of between-subjects effects showed lower cortical surface area values in patients with FND compared with HC participants in the left postcentral gyrus (*d* = 0.247, *p* = .018).

For a summary of the MANCOVAs using absolute surface area, see Table S4.

### Subcortical Volumes

A main effect was observed for FND (*F*_20,902_ = 2.003, *d* = 0.42*, p* = .006), age (*F*_20,902_ = 17.099, *d* = 1.22*, p* < .001), sex (*F*_20,902_ = 4.399, *d* = 0.60*, p* < .001), and eTIV (*F*_20,902_ = 46.032, *d* = 2.00*, p* < .001).

Pairwise comparisons of between-subjects effect revealed lower volume values in patients with FND compared with HC participants in the right hippocampus (*d* = 0.22*, p* = .018).

### Leave-One-Site-Out Analysis (Whole Group)

See the Supplemental Results.

### Association With Clinical Characteristics

#### Illness Duration.

Illness duration data were available for 384 patients (77.9% sample), and mean illness duration in the overall group was 5.2 years (SD = 5.9; minimum = 0, maximum = 35 years; median = 3 years). There were no significant correlations between illness duration and any of the regions on any of the morphometrics (*p* > .10).

#### Depression and Anxiety.

Lifetime depression data were available for 306 patients (62.1% of the sample). Lifetime anxiety data were also available for 306 patients (62.1% of the sample). Insufficient clinical data were available for all other clinical variables and for HC participants.

#### Depression.

After propensity score matching, 118 (88.06%) of the patients with a lifetime history of depression were matched to participants with FND without a lifetime history of depression (see Table S8).

There was no overall effect of depression on cortical thickness (*p* = .099), surface area (relative) (*p* = .436), or subcortical volumes (*p* = .255).

#### Anxiety.

After propensity score matching, 105 (80.15%) of the patients with anxiety were matched to participants with FND without a lifetime history of anxiety (see Table S9).

Three was no overall effect of anxiety on cortical thickness (*p* = .560), cortical surface area (relative) (*p* = .128), or subcortical volumes (*p* = .136).

### Subgroup Comparisons

In total, 192 patients with FND-seiz (99.5%) were matched with HC participants, and 307 patients with FND-motor (97.8%) were matched with HC participants (Table S10).

### Functional Motor Symptoms

#### Cortical Thickness.

There was a main effect of FND-motor (*F*_113,497_ = 1.665, *d* = 1.232, *p* < .001), age (*F*_113,497_ = 6.614, *d* = 2.456, *p* < .001), Euler number (*F*_113,497_ = 3.064, *d* = 1.671, *p* < .001), and sex (*F*_113,497_ = 2.891, *d* = 1.622, *p* < .001).

Pairwise comparisons of between-subjects effects revealed lower thickness in patients with FND-motor compared with HC participants in 9 individual regions: the bilateral superior frontal sulcus (left *d* = 0.249, *p* = .0358; right *d* = 0.295, *p* = .0108), bilateral superior frontal gyrus (left *d* = 0.234, *p* = .0486; right *d* = 0.267, *p* = .0200), left postcentral sulcus (*d* = 0.312, *p* = .0108), left superior precentral sulcus (*d* = 0.297, *p* = .0108), right paracentral gyrus and sulcus (*d* = 0.266, *p* = .0200), right precentral gyrus (*d* = 0.286, *p* = .0128), and right inferior precentral sulcus (*d* = 0.244, *p* = .0383) (Figure 2).

#### Cortical Surface Area (Relative).

There was a main effect of FND-motor (*F*_87,523_ = 1.334, *d* = 0.943, *p* = .032), age (*F*_87,523_ = 3.176, *d* = 1.454, *p* < .001), mean Euler number (*F*_87,523_ = 3.852, *d* = 1.602, *p* < .001), and sex (*F*_87,523_ = 2.589, *d* = 1.312, *p* < .001).

No individual regions were significant in pairwise comparisons.

#### Subcortical Volumes.

There was a main effect of FND-motor (*F*_20,590_ = 1.600, *d* = 0.46*, p* = .047), age (*F*_20,590_ = 12.299, *d* = 1.35*, p* < .001), eTIV (*F*_20,590_ = 30.093, *d* = 2.00*, p* < .001), and sex (*F*_20,590_ = 3.182, *d* = 0.63*, p* < .001).

No individual regions were significant on pairwise comparisons.

### Functional Seizures

#### Cortical Thickness.

There was a main effect of FND-seiz (*F*_44,335_ = 1.883, *d* = 0.993, *p* = .0011), age (*F*_44,335_ = 5.572, *d* = 1.712, *p* < .001), mean Euler number (*F*_44,225_ = 1.976, *d* = 1.019, *p* < .001), and sex (*F*_44,335_ = 1.727, *d* = 0.952, *p* = .004).

Pairwise comparisons revealed higher thickness in the right cuneal gyrus (*d* = 0.350, *p* = .0178) and right lateral orbital sulcus (*d* = 0.348, *p* = .0178) (see Figure 3).

#### Cortical Surface Area (Relative).

There was no overall effect of functional seizures (*p* = .097).

#### Subcortical Volumes.

There was no overall effect of functional seizures (*p* = .204).

### Comparing FND-Motor and FND-Seiz

A matched dataset of 138 participants with FND-motor and 138 participants with FND-seiz was created (Table S11).

#### Cortical Thickness.

There was a main effect of age (*F*_91,252_ = 4.026, *d* = 2.408 *p* < .001), sex (*F*_91,252_ = 2.257, *d* = 1.805, *p* < .001), mean Euler number (*F*_91,252_ = 2.684, *d* = 1.968, *p* < .001), and diagnosis (*F*_91,252_ = 2.021, *d* = 1.709, *p* < .001).

No regions were significantly different between groups.

#### Cortical Surface Area (Relative).

There was a main effect of age (*F*_62,281_ = 2.052, *d* = 1.347, *p* < .001), sex (*F*_62,281_ = 1.950, *d* = 1.312, *p* < .001), mean Euler number (*F*_62,281_ = 2.442, *d* = 1.468, *p* < .001), and diagnosis (*F*_62,281_ = 1.871, *d* = 1.284, *p* < .001).

No individual regions were significantly different between groups.

#### Subcortical Volumes.

There was a main effect of age (*F*_20,324_ = 6.071, *d* = 1.21, *p* < .001), sex (*F*_20,324_ = 2.811, *d* = 0.78*, p* < .001), eTIV (*F*_20,324_ = 20.091, *d* = 2.24*, p* < .001), and diagnosis (*F*_20,324_ = 1.616, *d* = 0.61*, p* = .047).

No individual regions were significantly different between regions.

### Sex Comparison (FND Group)

A matched dataset was created that comprised 119 females with FND and 119 males with FND (see Table S12).

#### Cortical Thickness.

There was a main effect of age (*F*_95,140_ = 3.638, *d* = 3.09, *p* < .001), mean Euler number (*F*_95,140_ = 1.991, *d* = 2.33, *p* < .001), and sex (*F*_95,140_ = 1.622, *d* = 2.07, *p* = .013).

No individual regions were significantly different between groups.

#### Cortical Surface Area (Relative).

There was no overall effect of sex (*p* = .121).

#### Subcortical Volumes.

There was a main effect of sex (*F*_20,215_ = 1.990, *d* = 0.81*, p* = .009), age (*F*_20,215_ = 5.245, *d* = 1.46*, p* < .001), and eTIV (*F*_20,215_ = 15.390, *d* = 2.32*, p* < .001).

No individual regions were significantly different between groups.

### Principal Component Analysis

#### Full FND Cohort: Cortical Thickness.

The PCA returned 2 dimensions with eigenvalues >1 explaining 64.80% of the total variance. The principal components for dimension 1 (eigenvalue = 5.99, 54.41% of the variance) were the bilateral superior precentral gyri and sulci, bilateral superior frontal gyri, and the right precentral gyrus (Figure S11). The principal components for dimension 2 (eigenvalue = 1.14, 10.39% of the variance) were the bilateral paracentral gyri and sulci and the bilateral superior frontal sulci (Figure 4).

## DISCUSSION

In this mega-analysis of structural MRI in FND, we identified subtle but consistent differences in brain morphology compared with HC participants, particularly lower cortical thickness. These findings are consistent with current conceptualizations of FND as predominantly a disorder of functional network alterations rather than consistent macroscopic structural changes (14,47). Nevertheless, effect sizes were of a small magnitude. At around 0.05 mm, the observed differences were also small; average cortical thickness is estimated to be around 2.5 mm, so regional differences represented up to approximately 2% of average thickness (48). Such changes are within the range of test-retest variability for most scanners.

The presence of subtle morphological changes across a large multisite dataset strengthens the evidence for structural correlates of FND and provides important guidance for future work. The study suggests that the amalgamation of multiple datasets in disorders such as FND is achievable, encouraging the future use of large data approaches that may help to surmount some of the limitations of smaller-scale studies and provide granular linkage between pathophysiology and illness presentations. Furthermore, the inclusion of Euler numbers suggests that differences in cortical thickness and relative surface area between FND and HC participants are not fully accounted for by head motion.

There were lower cortical thicknesses in regions responsible for motor control, including the precentral and paracentral areas, as well as planning and initiation of movement, such as the superior frontal gyri. Most of the regions that showed significant differences at the FND cohort group level also showed them at the FND-motor group level, suggesting that the lower values may be particularly associated with changes in motor abilities. These findings are consistent with a dataset outside of this analysis in which reduced cortical thickness was found in right paracentral and precentral regions (18).

In this study, there were lower thicknesses in regions responsible for motor planning and initiation. These alterations may reflect preexisting predisposing vulnerabilities, such as impaired prefrontal regulation of motor or emotional processing, consistent with theoretical models of corticolimbic dysfunction (49). Alternatively, they could be consequences of chronic symptoms, stress, or aberrant neuroplastic changes. The lack of correlation with illness duration leans toward a mechanistically important role (or trait marker), but longitudinal studies are needed to clarify these possibilities, especially in view of functional MRI studies that have shown duration-activation relationships (50,51).

Many regions previously identified as significant in smaller, single-site studies were not replicated in this large-scale analysis. This may reflect the increased statistical rigor and heterogeneity introduced through data harmonization, but it also underscores the importance of mega-analytic approaches in identifying findings with the highest generalizability across patient populations.

We did not observe alterations in regions commonly implicated in emotional processing in FND, such as the amygdala. One possible explanation is that variability in amygdala volume across individuals might have resulted in net null effects at the group level. It would be informative to assess structural changes in more granular detail, for example via interrogation of amygdalar nuclei or hippocampal subfields, to investigate whether FND is associated with increased heterogeneity in limbic regions (52). We also did not find any changes in the temporoparietal junction, a region implicated in self-agency and action-ownership, which has been suggested to be involved in the pathogenesis of FND (53).

### Subgroup Interpretations

There was an absence of significant structural differences between the FND-motor and FND-seiz subgroups, although the subtype comparisons with HC participants showed different patterns of change. Several factors may explain this result. First, the shared pathophysiology may manifest in subtle, overlapping alterations (e.g., in frontal or limbic regions) that are not sufficiently distinct to yield significant subgroup differences in morphometric measures. Second, our sample, while large, may lack sufficient power to detect small subtype-specific effects, particularly given the heterogeneity of FND presentations.

The current data did not allow us to fully explore other potential sources of variance, including the influence of comorbid depression, anxiety, or sex-based effects. These are factors that have shown variable associations with brain structure in previous FND studies (35,54,55). These limitations underscore the need for even larger, stratified datasets and complementary imaging modalities to parse out subgroup-specific differences.

Patients in the FND-seiz group showed increased right orbitofrontal thickness. While long-term treatment with selective serotonin reuptake inhibitors (56), lithium (57), and some antipsychotics (58) has been associated with prefrontal cortical thickening, we lacked sufficient medication history to assess this, and therefore such effects remain speculative in our sample.

The study did not reveal any differences in cortical thickness between patients with and without a lifetime depression history. Such changes might have been expected according to previous research (39). The fact that no differences were found in this large sample suggests that the methodology of assigning lifetime depression status might not have adequately separated the groups. We suggest that future imaging studies on FND attempt to record current and past depression status with increased granularity.

### Network-Level Interpretation

A potentially unifying theme among the implicated regions is their integration within distributed cortical networks involved in motor control, executive function, and interoceptive awareness. Many of the affected regions, such as the superior frontal gyrus, precentral gyrus, and inferior frontal regions, are components characterized by multifaceted intracortical connectivity and serve integrative roles across sensorimotor and cognitive domains (59–61). The structural convergence on such regions may point to shared microarchitectural vulnerabilities in FND, although this hypothesis warrants further study using histologically informed imaging metrics.

PCA identified 2 distinct dimensions of cortical thickness variation in the FND cohort, offering insight into potential network-level abnormalities. The primary component included the bilateral superior frontal gyri, superior precentral sulci, and inferior opercular gyrus, which are regions implicated in motor planning, cognitive control, and salience processing, suggesting an involvement of frontoparietal and salience networks (62,63).

Together, these components highlight a possible disruption in higher-order cognitive control, supporting the notion that multinetwork dysfunction underlies functional symptoms (11,62,64). The co-alteration suggests that aberrations in structure may influence or reflect maladaptive network dynamics; for example, the observed patterns could contribute to impaired top-down modulation of motor output or interoceptive prediction errors, which are core components of current theoretical accounts of FND via predictive coding frameworks (64).

Another notable absence was the lack of robust PC-based evidence for a single unifying structural network underlying FND. While some clustering of affected regions was observed, the diversity of implicated areas suggests that FND does not have a singular morphological signature. Instead, the disorder may involve multiple routes to network-level dysfunction (65), supporting a model of neurobiological degeneracy (many-to-one mapping), wherein diverse structural changes can give rise to similar phenotypic outcomes (66).

### Future Directions

Additional studies on structural data could include emerging methods such as source-based morphometry and mode-based morphometry (67,68). Mega-analytic approaches to other imaging modalities such as diffusion-weighted imaging or functional MRI may shed further light. Further granular analysis of structural imaging, particularly methods that incorporate deep phenotypic characterization, neurotransmitter analyses [e.g., neurotransmitter correlation analysis (69)], or genetic correlations (70,71), could help elucidate the underlying biological mechanisms. High-field-strength research is also needed to investigate potential mechanistically relevant roles for brainstem structures (72). Future studies could also explore how gray and white matter changes interact to support symptom generation and recovery (54).

The effect size of other (non-FND) factors on the cortex may be greater than that of FND and therefore obscure observable morphometric differences, even in a large study such as this. Nevertheless, FND-related microanatomical changes relevant to mechanism may still be present. This underscores the need for a better understanding of microanatomical changes in FND.

### Limitations

Despite the successful application of harmonization procedures to the data, this is unlikely to surmount all variability associated with site effects (73). We were also unable to characterize childhood maltreatment and adverse life experiences in our dataset despite them likely contributing (74). We were also unable to consider medication effects. Some of the included groups had small datasets. Although we used conservative post hoc corrections, this study utilized multiple comparisons through several different analyses.

## Conclusions

In this mega-analysis, we found subtly lower cortical thickness in motor and cognitive control regions in patients with FND compared with HC participants. These results are consistent with models of FND as a multinetwork disorder. Nevertheless, there are identifiable, although small, structural correlates that warrant further investigation.

This study provides a proof of concept for future big data approaches and offers several hypotheses for future work on the structural underpinnings of FND and granular analysis of genetic or neurotransmitter expression. Analysis of functional imaging data would be highly valuable in future investigations because alterations in functional networks may emerge even when structural changes are confined to a single region.

## Supplementary Material

Supplemental Methods

Supplementary material cited in this article is available online at https://doi.org/10.1016/j.biopsych.2026.06.031.

## Figures and Tables

**Figure 1.**
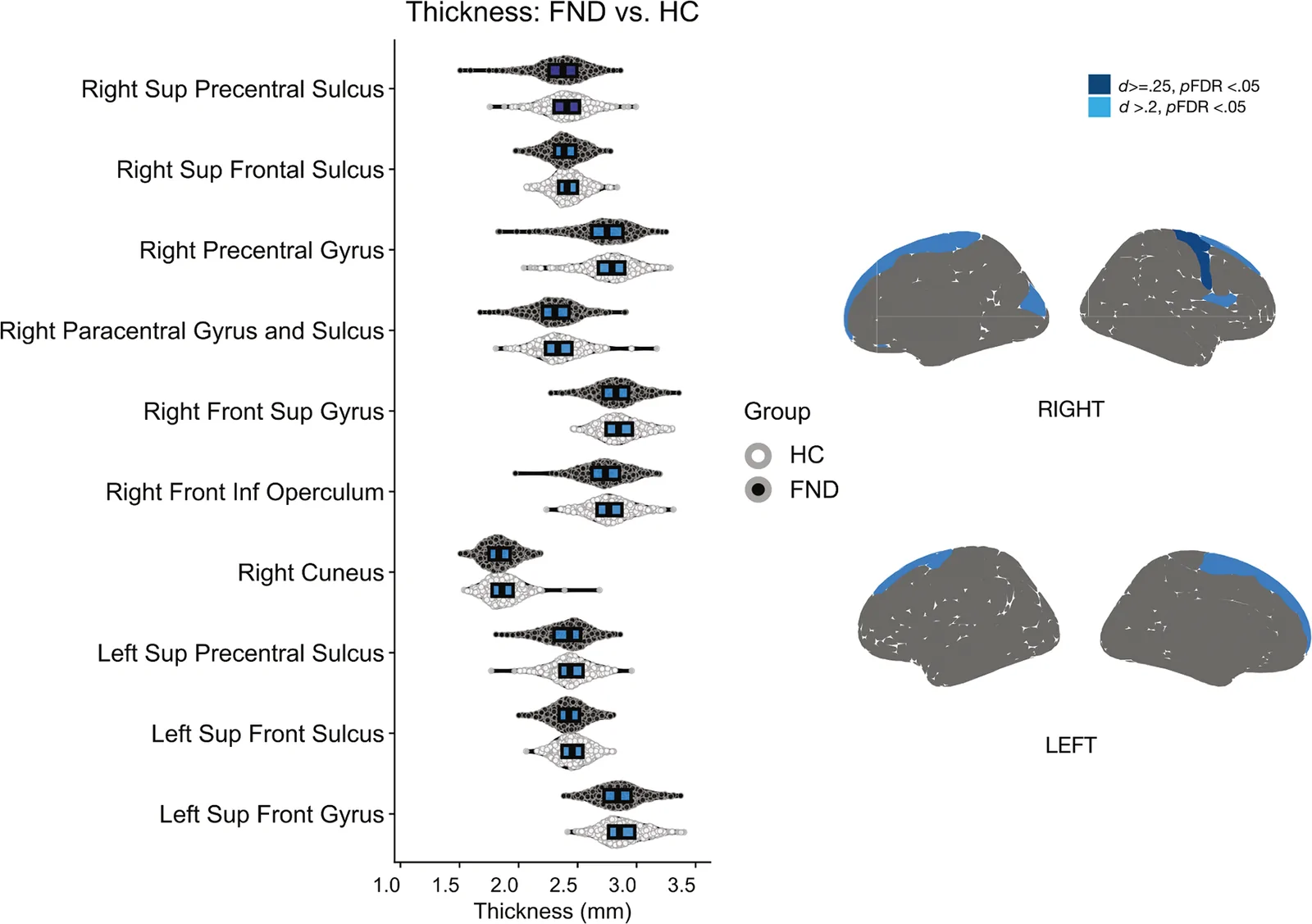
Regions with significant differences between patients with functional neurological disorder (FND) and healthy control (HC) participants in the cortical thickness morphometric. Regions are colored based on η_p_^2^ values obtained from pairwise comparisons. In every case, the group differences were of a small magnitude, as indicated by the box-and-whisker plot. FDR, false discovery rate.

**Figure 2.**
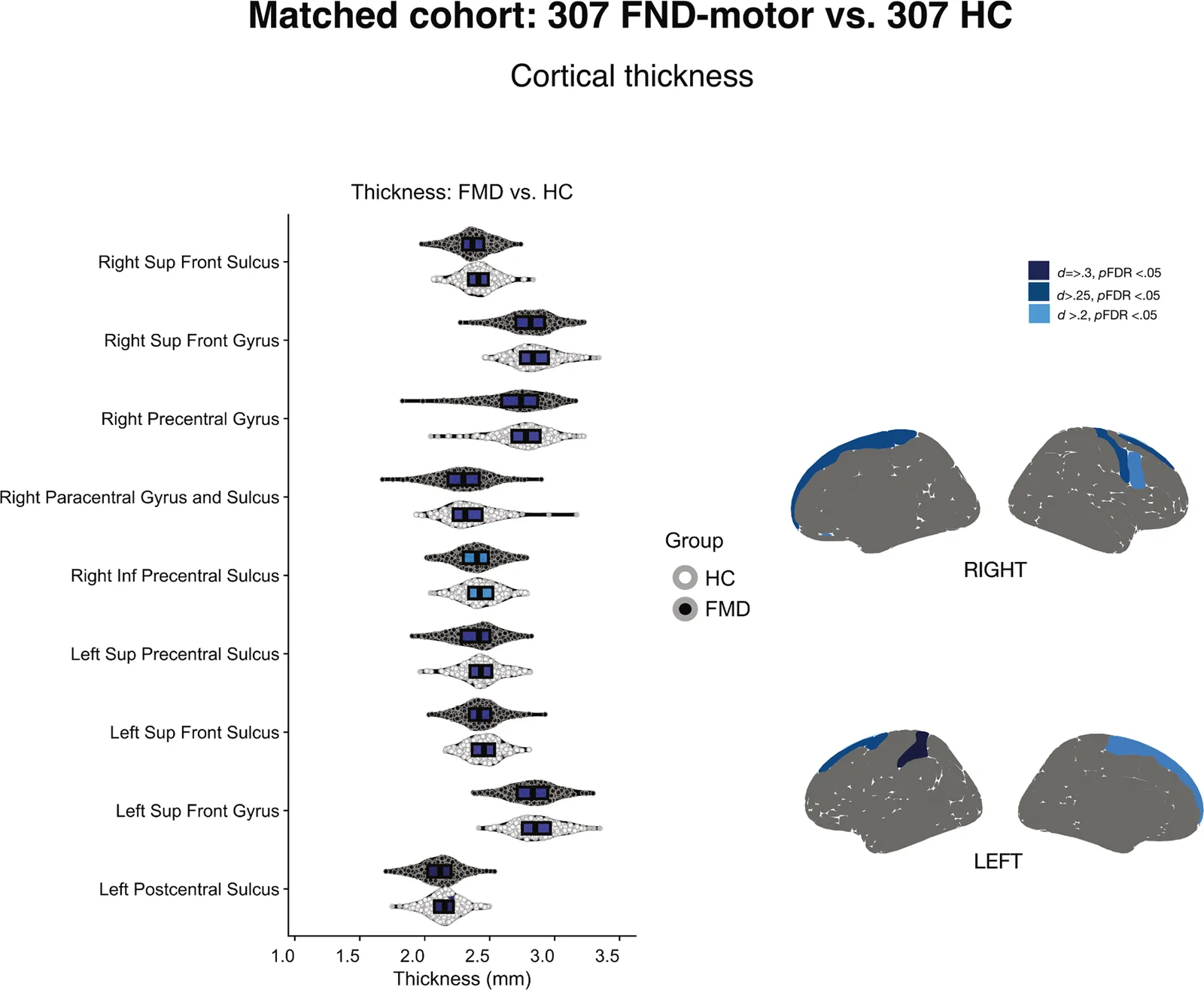
Regions showing a difference between patients with functional motor disorder (FMD) (functional neurological disorder [FND]-motor) and healthy control (HC) participants in the cortical thickness morphometric. In every region, thickness was lower in the FND-motor group. Regions are colored based on η_p_^2^ values obtained from pairwise comparisons. In every case, the group differences were of a small magnitude, as indicated by the violin plots. FDR, false discovery rate.

**Figure 3.**
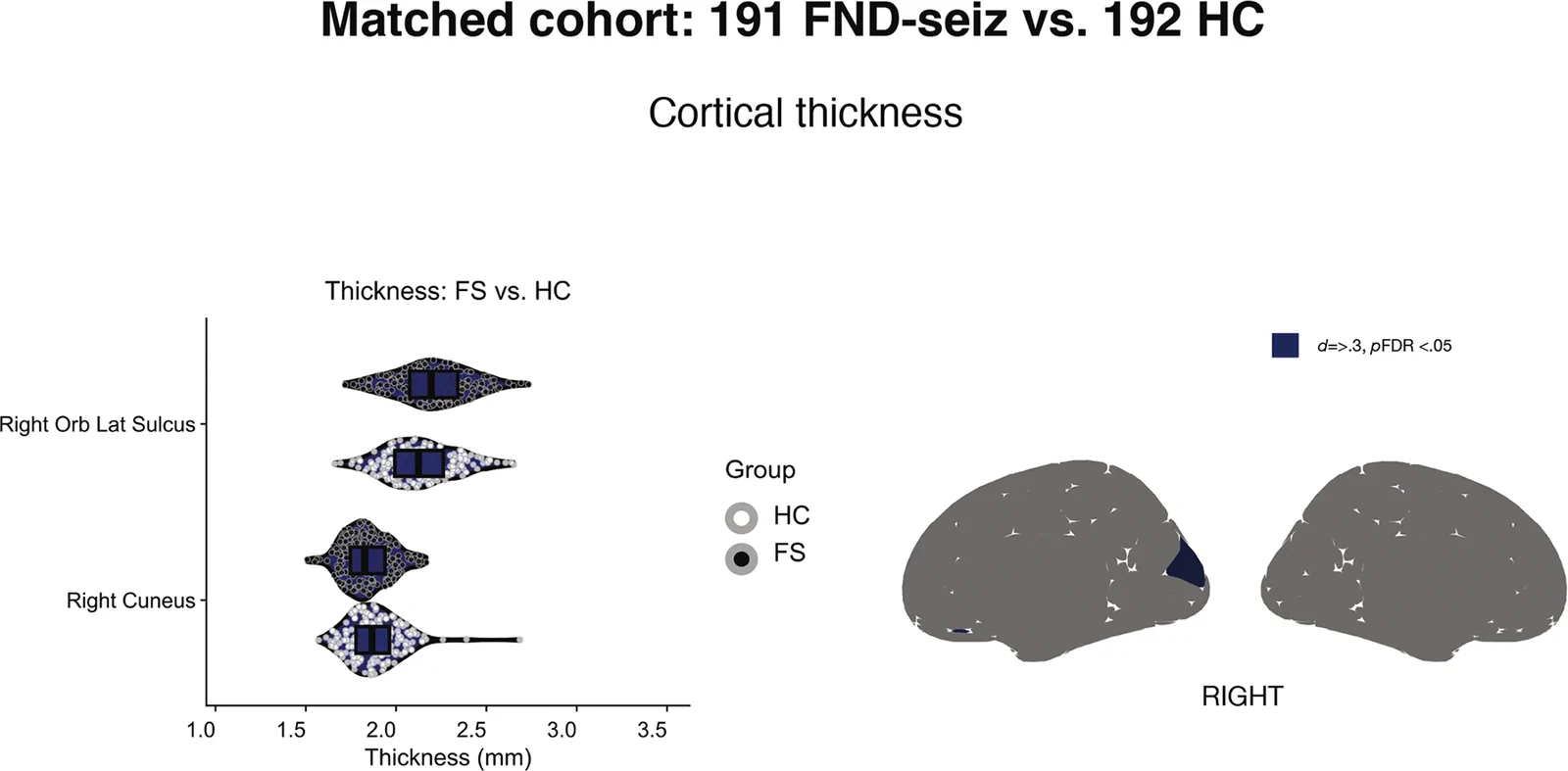
Regions showing a difference between patients with functional seizures (FS) and healthy control (HC) participants in the cortical thickness morphometric. Thickness was lower in the right cuneal and the right lateral orbital sulcus in the FS group. These regions are colored based on the η_p_^2^ value obtained from pairwise comparisons. The group difference was of a small magnitude, as indicated by the violin plot.

**Figure 4.**
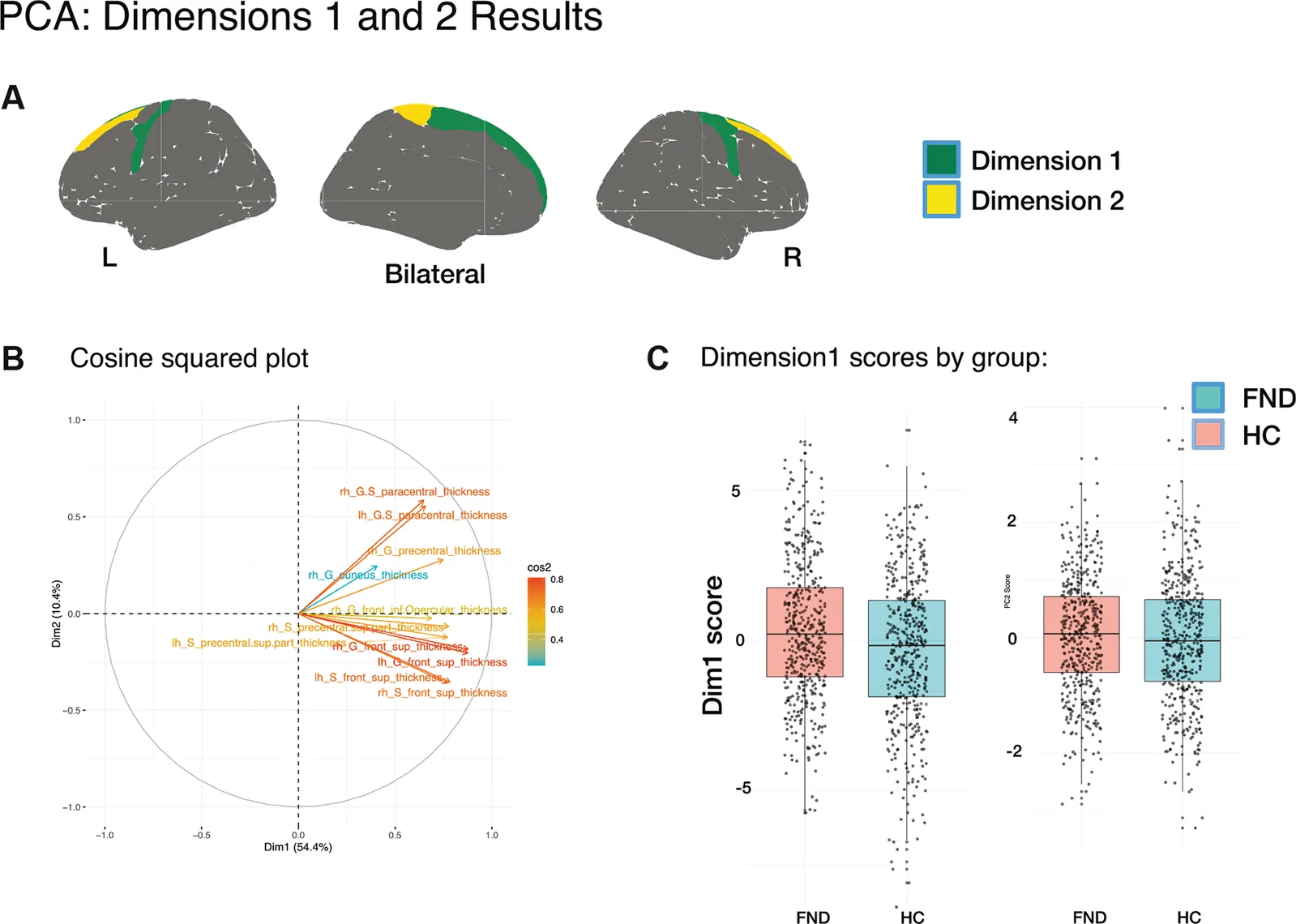
Principal component analysis (PCA) of cortical thickness measures. **(A)** Spatial visualization of loadings. Each cortical region is colored by the PC on which it shows the larger loading (for percentage contribution [squared loading/component eigenvalue × 100]; see Supplemental Figure S11. Regions mapping onto PC 1 (Dim-1, accounting for 54% of the total variance) are shown in green; those mapping onto PC 2 (Dim-2, 10%) are shown in yellow. The center panel shows both hemispheres. **(B)** Squared-cosine correlation circle. Arrows represent different regions, positioned by their loadings on Dim-1 (x-axis) and Dim-2 (y-axis). Arrow length and color scale represent the squared-cosine (cos^2^) value, which is an indicator of the proportion of a region’s variance that is captured by this 2-dimensional solution. Parcels clustering along the horizontal axis shape PC 1; those near the vertical axis are those that shape PC 2. **(C)** Component scores by group. Individual participants scores on PC 1 are overlaid on box-and-whisker plots for the functional neurological disorder (FND) group (cyan) and healthy control (HC) participants (pink). Boxes represent the interquartile range (IQR), the center line represents the median, and whiskers represent = ±1.5 × IQR; individual dots correspond to individual participants. PC1 showed meaningful group separation. L, left hemisphere; R, right hemisphere.

**Table 1.** Breakdown of Contributing Research Groups After Preparing the Dataset

Research Group	Study Group	*N*	Age, Years		Sex			
			Mean (SD)	*p*	Female, *n*	Male, *n*	χ^2^	*p*
Allendorfer *et al*., United States (75,76)	HC	20	36.7 (12.2)	.918	13	7	OR = 0.17^a^	.205
	FND	13	37.2 (12.8)		11	1		
Hassa *et al*., Germany (77–79)	HC	27	46.5 (13.4)	.305	14	13	0.682	.409
	FND	27	42.4 (15.5)		17	10		
Horovitz *et al*., United States (24,80,81)	HC	52	44.1 (11.6)	.912	39	13	0.344	.557
	FND	59	43.9 (12.0)		47	12		
Jansen *et al*., Netherlands (82,83)	HC	29	35.9 (11.8)	.698	21	8	0.448	.503
	FND	22	34.6 (12.0)		14	8		
Tijssen *et al*., Netherlands (84)	HC	17	43.3 (14.6)	.745	9	8	0.0289	.866
	FND	16	41.7 (12.4)		8	8		
Aybek *et al*., Switzerland (85–87)	HC	75	33.2 (11.0)	.114	56	19	0.035	.852
	FND	75	36.3 (13.1)		55	20		
Premi *et al*., Italy (88) (+ Unpublished Data)	HC	15	41.3 (9.2)	.701	15	0	2.245^b^	.134
	FND	7	43.2 (13.2)		6	1		
Ristic *et al*., Serbia (26)	HC	20	43.1 (10.8)	.209	15	5	OR = 0.47^a^	.445
	FND	22	38.3 (13.5)		19	3		
Serranova *et al*., Czechia (Unpublished Data)	HC	60	43.0 (9.9)	.187	45	15	0.002	.967
	FND	83	40.6 (11.5)		62	21		
Sojka *et al*., Czechia (89)	HC	38	33.5 (13.8)	.872	30	8	0.013	.908
	FND	40	33.0 (13.3)		32	8		
Sone *et al*., Japan (54)	HC	26	29.7 (6.9)	.716	20	6	OR = 1.03^a^	1.00
	FND	17	28.8 (9.4)		13	4		
Kerr *et al*., United States (Unpublished Data)	HC	126	36.2 (14.2)	.873	80	46	5.096	.024
	FND	52	35.9 (11.3)		42	10		
Nicholson *et al*., United Kingdom (21)	HC	12	36.6 (7.8)	.758	8	4	OR = 0.676^a^	.691
	FND	14	37.8 (11.3)		12	4		
Perez *et al*., United States (19,35)	HC	27	40.5 (10.8)	.975	22	5	0.004	.947
	FND	26	40.4 (11.5)		21	5		
Jungilligens *et al*., Germany (90)	HC	20	36.6 (12.2)	.686	15	5	0.00	1.00
	FND	20	35.1 (11.2)		15	5		
Total	HC	564	38.1 (12.8)	.884	402	162	2.34	.126
	FND	493	38.2 (12.7)		372	121		

FND, functional neurological disorder; HC, healthy control; OR, odds ratio.

a Fisher’s exact test was performed due to low numbers.

b Unable to complete Fisher’s exact test due to 0 value, so a χ^2^ test was performed.

**KEY RESOURCES TABLE:** 

Resource Type	Specific Reagent or Resource	Source or Reference	Identifiers	Additional Information
Add additional rows as needed for each resource type	Include species and sex when applicable.	Include name of manufacturer, company, repository, individual, or research lab. Include PMID or DOI for references; use “this paper” if new.	Include catalog numbers, stock numbers, database IDs or accession numbers, and/or RRIDs. RRIDs are highly encouraged; search for RRIDs at https://scicrunch.org/resources.	Include any additional information or notes if necessary.
Antibody	N/A			
Bacterial or Viral Strain	N/A			
Biological Sample	N/A			
Cell Line	N/A			
Chemical Compound or Drug	N/A			
Commercial Assay Or Kit	N/A			
Deposited Data; Public Database	N/A			
Genetic Reagent	N/A			
Organism/Strain	N/A			
Peptide, Recombinant Protein	N/A			
Recombinant DNA	N/A			
Sequence-Based Reagent	N/A			
Software; Algorithm	Freesurfer 6.0.0; SPSS 24; R 4.0.0	Laboratory for Computational Neuroimaging at the Athinoula A. Martinos Center for Biomedical Imaging.; IBM Corp.; R core team	https://surfer.nmr.mgh.harvard.edu/fswiki/rel6downloads; https://www.ibm.com/support/pages/downloading-ibm-spss-statistics-24; 10.1006/nimg.1998.0395	
Transfected Construct	N/A			
Other	N/A			

## References

[1] Finkelstein SA, Diamond C, Carson A, Stone J (2025): Incidence and prevalence of functional neurological disorder: A systematic review. J Neurol Neurosurg Psychiatry 96:383–395. 10.1136/jnnp-2024-334767 PMC12015090 39663114

[2] Stephen CD, Fung V, Perez DL, Espay AJ (2025): Comparison of inpatient and emergency department costs to research funding for functional neurologic disorder: An economic analysis. Neurology 104:e213445. 10.1212/WNL.0000000000213445 PMC11863780 39999398

[3] Gelauff JM, Carson A, Ludwig L, Tijssen MAJ, Stone J (2019): The prognosis of functional limb weakness: A 14-year case-control study. Brain 142:2137–2148. 10.1093/brain/awz138 31167232

[4] Espay AJ, Aybek S, Carson A, Edwards MJ, Goldstein LH, Hallett M, (2018): Current concepts in diagnosis and treatment of functional neurological disorders. JAMA Neurol 75:1132–1141. 10.1001/jamaneurol.2018.1264 PMC7293766 29868890

[5] Stone J, Carson A, Duncan R, Roberts R, Warlow C, Hibberd C, (2010): Who is referred to neurology clinics?—The diagnoses made in 3781 new patients. Clin Neurol Neurosurg 112:747–751. 10.1016/j.clineuro.2010.05.011 20646830

[6] Tinazzi M, Morgante F, Marcuzzo E, Erro R, Barone P, Ceravolo R, (2020): Clinical correlates of functional motor disorders: An Italian multicenter study. Mov Disord Clin Pract 7:920–929. 10.1002/mdc3.13077 PMC7604660 33163563

[7] Carson A, Lehn A (2016): Epidemiology. Handb Clin Neurol 139:47–60. 10.1016/B978-0-12-801772-2.00005-9 27719864

[8] Carson A, Stone J, Hibberd C, Murray G, Duncan R, Coleman R, (2011): Disability, distress and unemployment in neurology outpatients with symptoms ‘unexplained by organic disease’. J Neurol Neurosurg Psychiatry 82:810–813. 10.1136/jnnp.2010.220640 21257981

[9] Jones B, Reuber M, Norman P (2016): Correlates of health-related quality of life in adults with psychogenic nonepileptic seizures: A systematic review. Epilepsia 57:171–181. 10.1111/epi.13268 26701628

[10] Nicholson TR, Carson A, Edwards MJ, Goldstein LH, Hallett M, Mildon B, (2020): Outcome measures for functional neurological disorder: A review of the theoretical complexities. J Neuropsychiatry Clin Neurosci 32:33–42. 10.1176/appi.neuropsych.19060128 31865871

[11] Perez DL, Nicholson TR, Asadi-Pooya AA, Bègue I, Butler M, Carson AJ, (2021): Neuroimaging in functional neurological disorder: State of the field and research agenda. NeuroImage Clin 30:102623. 10.1016/j.nicl.2021.102623 PMC8111317 34215138

[12] Maguire EA, Gadian DG, Johnsrude IS, Good CD, Ashburner J, Frackowiak RS, Frith CD (2000): Navigation-related structural change in the hippocampi of taxi drivers. Proc Natl Acad Sci U S A 97:4398–4403. 10.1073/pnas.070039597 PMC18253 10716738

[13] Nixon NL, Liddle PF, Nixon E, Worwood G, Liotti M, Palaniyappan L (2014): Biological vulnerability to depression: Linked structural and functional brain network findings. Br J Psychiatry 204:283–289. 10.1192/bjp.bp.113.129965 24357570

[14] Bègue I, Adams C, Stone J, Perez DL (2019): Structural alterations in functional neurological disorder and related conditions: A software and hardware problem? NeuroImage Clin 22:101798. 10.1016/j.nicl.2019.101798 PMC6484222 31146322

[15] Mcsweeney M, Reuber M, Levita L (2017): Neuroimaging studies in patients with psychogenic non-epileptic seizures: A systematic meta-review. NeuroImage Clin 16:210–221. 10.1016/j.nicl.2017.07.025 PMC5544493 28808618

[16] Rossetti MG, Delvecchio G, Calati R, Perlini C, Bellani M, Brambilla P (2021): Structural neuroimaging of somatoform disorders: A systematic review. Neurosci Biobehav Rev 122:66–78. 10.1016/j.neubiorev.2020.12.017 33359097

[17] Aybek S, Nicholson TRJ, Draganski B, Daly E, Murphy DG, David AS, Kanaan RA (2014): Grey matter changes in motor conversion disorder. J Neurol Neurosurg Psychiatry 85:236–238. 10.1136/jnnp-2012-304158 23236016

[18] Labate A, Cerasa A, Mula M, Mumoli L, Gioia MC, Aguglia U, (2012): Neuroanatomic correlates of psychogenic non-epileptic seizures: A cortical thickness and VBM study. Epilepsia 53:377–385. 10.1111/j.1528-1167.2011.03347.x 22150750

[19] Perez DL, Matin N, Williams B, Tanev K, Makris N, LaFrance WC, Dickerson BC (2018): Cortical thickness alterations linked to somatoform and psychological dissociation in functional neurological disorders. Hum Brain Mapp 39:428–439. 10.1002/hbm.23853 PMC5747307 29080235

[20] Jungilligens J, Popkirov S, Perez DL, Diez I (2022): Linking gene expression patterns and brain morphometry to trauma and symptom severity in patients with functional seizures. Psychiatry Res Neuroimaging 326:111533. 10.1016/j.pscychresns.2022.111533 PMC9968826 36055038

[21] Nicholson TR, Aybek S, Kempton MJ, Daly EM, Murphy DG, David AS, Kanaan RA (2014): A structural MRI study of motor conversion disorder: Evidence of reduction in thalamic volume. J Neurol Neurosurg Psychiatry 85:227–229. 10.1136/jnnp-2013-305012 24039028

[22] Atmaca M, Aydin A, Tezcan E, Poyraz AK, Kara B (2006): Volumetric investigation of brain regions in patients with conversion disorder. Prog Neuropsychopharmacol Biol Psychiatry 30:708–713. 10.1016/j.pnpbp.2006.01.011 16600450

[23] Espay AJ, Maloney T, Vannest J, Norris MM, Eliassen JC, Neefus E, (2018): Impaired emotion processing in functional (psychogenic) tremor: A functional magnetic resonance imaging study. NeuroImage Clin 17:179–187. 10.1016/j.nicl.2017.10.020 PMC5655406 29085776

[24] Maurer CW, LaFaver K, Limachia GS, Capitan G, Ameli R, Sinclair S, (2018): Gray matter differences in patients with functional movement disorders. Neurology 91:e1870–e1879. 10.1212/WNL.0000000000006514 PMC6260194 30305450

[25] Kerr WT, Tatekawa H, Lee JK, Karimi AH, Sreenivasan SS, O’Neill J, (2022): Clinical MRI morphological analysis of functional seizures compared to seizure-naïve and psychiatric controls. Epilepsy Behav 134:108858. 10.1016/j.yebeh.2022.108858 35933959

[26] Ristić AJ, Daković M, Kerr M, Kovačević M, Parojčić A, Sokić D (2015): Cortical thickness, surface area and folding in patients with psychogenic nonepileptic seizures. Epilepsy Res 112:84–91. 10.1016/j.eplepsyres.2015.02.015 25847343

[27] Mcsweeney M, Reuber M, Hoggard N, Levita L (2018): Cortical thickness and gyrification patterns in patients with psychogenic non-epileptic seizures. Neurosci Lett 678:124–130. 10.1016/j.neulet.2018.04.056 29763635

[28] Sharma AA, Goodman AM, Allendorfer JB, Philip NS, Correia S, LaFrance WC, Szaflarski JP (2022): Regional brain atrophy and aberrant cortical folding relate to anxiety and depression in patients with traumatic brain injury and psychogenic nonepileptic seizures. Epilepsia 63:222–236. 10.1111/epi.17109 PMC8742780 34730239

[29] Tatekawa H, Kerr WT, Savic I, Engel J, Salamon N (2020): Reduced left amygdala volume in patients with dissociative seizures (psychogenic nonepileptic seizures). Seizure 75:43–48. 10.1016/j.seizure.2019.12.014 31874358

[30] Vasta R, Cerasa A, Sarica A, Bartolini E, Martino I, Mari F, (2018): The application of artificial intelligence to understand the pathophysiological basis of psychogenic nonepileptic seizures. Epilepsy Behav 87:167–172. 10.1016/j.yebeh.2018.09.008 30269939

[31] Williams B, Jalilianhasanpour R, Matin N, Fricchione GL, Sepulcre J, Keshavan MS, (2018): Individual differences in corticolimbic structural profiles linked to insecure attachment and coping styles in motor functional neurological disorders. J Psychiatr Res 102:230–237. 10.1016/j.jpsychires.2018.04.006 PMC6005758 29702433

[32] Atmaca M, Baykara S, Mermi O, Yildirim H, Akaslan U (2016): Pituitary volumes are changed in patients with conversion disorder. Brain Imaging Behav 10:92–95. 10.1007/s11682-015-9368-6 25877743

[33] Ospina JP, Larson AG, Jalilianhasanpour R, Williams B, Diez I, Dhand A, (2019): Individual differences in social network size linked to nucleus accumbens and hippocampal volumes in functional neurological disorder: A pilot study. J Affect Disord 258:50–54. 10.1016/j.jad.2019.07.061 PMC6783807 31394458

[34] Perez DL, Williams B, Matin N, Mello J, Dickerson BC, LaFrance WC Jr, Keshavan MS (2018): Anterior hippocampal grey matter predicts mental health outcome in functional neurological disorders: An exploratory pilot study. J Neurol Neurosurg Psychiatry 89:1221–1224. 10.1136/jnnp-2017-317305 PMC6041189 29326291

[35] Perez DL, Williams B, Matin N, LaFrance WC, Costumero-Ramos V, Fricchione GL, (2017): Corticolimbic structural alterations linked to health status and trait anxiety in functional neurological disorder. J Neurol Neurosurg Psychiatry 88:1052–1059. 10.1136/jnnp-2017-316359 28844071

[36] Butler M, Shipston-Sharman O, Seynaeve M, Bao J, Pick S, Bradley-Westguard A, (2021): International online survey of 1048 individuals with functional neurological disorder. Eur J Neurol 28:3591–3602. 10.1111/ene.15018 34245646

[37] Vignando M, Ffytche D, Lewis SJG, Lee PH, Chung SJ, Weil RS, (2022): Mapping brain structural differences and neuroreceptor correlates in Parkinson’s disease visual hallucinations. Nat Commun 13:519. 10.1038/s41467-022-28087-0 PMC8791961 35082285

[38] Whelan CD, Altmann A, Botía JA, Jahanshad N, Hibar DP, Absil J, (2018): Structural brain abnormalities in the common epilepsies assessed in a worldwide ENIGMA study. Brain 141:391–408. 10.1093/brain/awx341 PMC5837616 29365066

[39] Schmaal L, Pozzi E, C Ho T, Van Velzen LS, Veer IM, Opel N, (2020): ENIGMA MDD: Seven years of global neuroimaging studies of major depression through worldwide data sharing. Transl Psychiatry 10:172. 10.1038/s41398-020-0842-6 PMC7260219 32472038

[40] Panizzon MS, Fennema-Notestine C, Eyler LT, Jernigan TL, Prom-Wormley E, Neale M, (2009): Distinct genetic influences on cortical surface area and cortical thickness. Cereb Cortex 19:2728–2735. 10.1093/cercor/bhp026 PMC2758684 19299253

[41] Dale AM, Fischl B, Sereno MI (1999): Cortical surface-based analysis. I. Segmentation and surface reconstruction. NeuroImage 9:179–194. 10.1006/nimg.1998.0395 9931268

[42] Destrieux C, Fischl B, Dale A, Halgren E (2010): Automatic parcellation of human cortical gyri and sulci using standard anatomical nomenclature. NeuroImage 53:1–15. 10.1016/j.neuroimage.2010.06.010 PMC2937159 20547229

[43] Radua J, Vieta E, Shinohara R, Kochunov P, Quidé Y, Green MJ, (2020): Increased power by harmonizing structural MRI site differences with the ComBat batch adjustment method in ENIGMA. NeuroImage 218:116956. 10.1016/j.neuroimage.2020.116956 PMC7524039 32470572

[44] Fortin JP, Cullen N, Sheline YI, Taylor WD, Aselcioglu I, Cook PA, (2018): Harmonization of cortical thickness measurements across scanners and sites. NeuroImage 167:104–120. 10.1016/j.neuroimage.2017.11.024 PMC5845848 29155184

[45] Alexander-Bloch A, Clasen L, Stockman M, Ronan L, Lalonde F, Giedd J, Raznahan A (2016): Subtle in-scanner motion biases automated measurement of brain anatomy from in vivo MRI. Hum Brain Mapp 37:2385–2397. 10.1002/hbm.23180 PMC5110234 27004471

[46] Rosen AFG, Roalf DR, Ruparel K, Blake J, Seelaus K, Villa LP, (2018): Quantitative assessment of structural image quality. NeuroImage 169:407–418. 10.1016/j.neuroimage.2017.12.059 PMC5856621 29278774

[47] Coebergh J, Habib S, Teodoro T, Edwards M, Butler M (2024): From software to hardware: A case series of functional neurological symptoms and cerebrovascular disease. J Neuropsychiatry Clin Neurosci 36:206–213. 10.1176/appi.neuropsych.20220182 38343312

[48] Fischl B, Dale AM (2000): Measuring the thickness of the human cerebral cortex from magnetic resonance images. Proc Natl Acad Sci USA 97:11050–11055. 10.1073/pnas.200033797 PMC27146 10984517

[49] Diez I, Ortiz-Terán L, Williams B, Jalilianhasanpour R, Ospina JP, Dickerson BC, (2019): Corticolimbic fast-tracking: Enhanced multimodal integration in functional neurological disorder. J Neurol Neurosurg Psychiatry 90:929–938. 10.1136/jnnp-2018-319657 PMC6625895 30850473

[50] Szaflarski JP, Allendorfer JB, Goodman AM, Byington CG, Philip NS, Correia S, LaFrance WC (2022): Diagnostic delay in functional seizures is associated with abnormal processing of facial emotions. Epilepsy Behav 131:108712. 10.1016/j.yebeh.2022.108712 35526462

[51] Goodman AM, Allendorfer JB, LaFrance WC, Szaflarski JP (2022): Precentral gyrus and insula responses to stress vary with duration to diagnosis in functional seizures. Epilepsia 63:865–879. 10.1111/epi.17179 35112346

[52] Westlin C, Guthrie AJ, Paredes-Echeverri S, Maggio J, Finkelstein S, Godena E, (2025): Machine learning classification of functional neurological disorder using structural brain MRI features. J Neurol Neurosurg Psychiatry 96:249–257. 10.1136/jnnp-2024-333499 PMC11743827 39033019

[53] Weber S, Bühler J, Bolton TAW, Aybek S (2025): Altered brain network dynamics in motor functional neurological disorders: The role of the right temporo-parietal junction. Transl Psychiatry 15:167. 10.1038/s41398-025-03385-5 PMC12081630 40374624

[54] Sone D, Sato N, Ota M, Kimura Y, Matsuda H (2019): Widely impaired white matter integrity and altered structural brain networks in psychogenic non-epileptic seizures. Neuropsychiatr Dis Treat 15:3549–3555. 10.2147/NDT.S235159 PMC6939397 31920315

[55] Gninenko N, Müller E, Aybek S (2025): Reduced microstructural white matter integrity is associated with the severity of physical symptoms in functional neurological disorder. NeuroImage Clin 46:103791. 10.1016/j.nicl.2025.103791 PMC12090312 40318503

[56] Nemati S, Abdallah CG (2020): Increased cortical thickness in patients with major depressive disorder following antidepressant treatment. Chronic Stress (Thousand Oaks) 4:2470547019899962. 10.1177/2470547019899962 PMC6959134 31938760

[57] Monkul ES, Matsuo K, Nicoletti MA, Dierschke N, Hatch JP, Dalwani M, (2007): Prefrontal gray matter increases in healthy individuals after lithium treatment: A voxel-based morphometry study. Neurosci Lett 429:7–11. 10.1016/j.neulet.2007.09.074 PMC2693231 17996370

[58] Ansell BRE, Dwyer DB, Wood SJ, Bora E, Brewer WJ, Proffitt TM, (2015): Divergent effects of first-generation and second-generation antipsychotics on cortical thickness in first-episode psychosis. Psychol Med 45:515–527. 10.1017/S0033291714001652 PMC4413868 25077698

[59] Li W, Qin W, Liu H, Fan L, Wang J, Jiang T, Yu C (2013): Subregions of the human superior frontal gyrus and their connections. NeuroImage 78:46–58. 10.1016/j.neuroimage.2013.04.011 23587692

[60] Sundermann B, Pfleiderer B (2012): Functional connectivity profile of the human inferior frontal junction: Involvement in a cognitive control network. BMC Neurosci 13:119. 10.1186/1471-2202-13-119 PMC3582543 23033990

[61] Du J, Rolls ET, Cheng W, Li Y, Gong W, Qiu J, Feng J (2020): Functional connectivity of the orbitofrontal cortex, anterior cingulate cortex, and inferior frontal gyrus in humans. Cortex 123:185–199. 10.1016/j.cortex.2019.10.012 31869573

[62] Drane DL, Fani N, Hallett M, Khalsa SS, Perez DL, Roberts NA (2021): A framework for understanding the pathophysiology of functional neurological disorder. CNS Spectr 26:555–561. 10.1017/S1092852920001789 PMC7930164 32883381

[63] Westlin C, Guthrie AJ, Bleier C, Finkelstein SA, Maggio J, Ranford J, (2025): Delineating network integration and segregation in the pathophysiology of functional neurological disorder. Brain Commun 7:fcaf195. 10.1093/braincomms/fcaf195 PMC12107243 40433115

[64] Edwards MJ, Adams RA, Brown H, Pareés I, Friston KJ (2012): A Bayesian account of ‘hysteria’. Brain 135:3495–3512. 10.1093/brain/aws129 PMC3501967 22641838

[65] Szaflarski JP, LaFrance WC (2018): Psychogenic nonepileptic seizures (PNES) as a network disorder—Evidence from neuroimaging of functional (psychogenic) neurological disorders. Epilepsy Curr 18:211–216. 10.5698/1535-7597.18.4.211 PMC6145391 30254510

[66] Westlin C, Theriault JE, Katsumi Y, Nieto-Castanon A, Kucyi A, Ruf SF, (2023): Improving the study of brain-behavior relationships by revisiting basic assumptions. Trends Cogn Sci 27:246–257. 10.1016/j.tics.2022.12.015 PMC10012342 36739181

[67] Cao T, Pang JC, Segal A, Chen YC, Aquino KM, Breakspear M, Fornito A (2024): Mode-based morphometry: A multiscale approach to mapping human neuroanatomy. Hum Brain Mapp 45:e26640. 10.1002/hbm.26640 PMC10915742 38445545

[68] Xu L, Groth KM, Pearlson G, Schretlen DJ, Calhoun VD (2009): Source-based morphometry: The use of independent component analysis to identify gray matter differences with application to schizophrenia. Hum Brain Mapp 30:711–724. 10.1002/hbm.20540 PMC2751641 18266214

[69] Dukart J, Holiga S, Rullmann M, Lanzenberger R, Hawkins PCT, Mehta MA, (2021): JuSpace: A tool for spatial correlation analyses of magnetic resonance imaging data with nuclear imaging derived neurotransmitter maps. Hum Brain Mapp 42:555–566. 10.1002/hbm.25244 PMC7814756 33079453

[70] Spagnolo PA, Johnson K, Hodgkinson C, Goldman D, Hallett M (2023): Methylome changes associated with functional movement/conversion disorder: Influence of biological sex and childhood abuse exposure. Prog Neuropsychopharmacol Biol Psychiatry 125:110756. 10.1016/j.pnpbp.2023.110756 PMC10205664 36958667

[71] Spagnolo PA, Norato G, Maurer CW, Goldman D, Hodgkinson C, Horovitz S, Hallett M (2020): Effects of TPH2 gene variation and childhood trauma on the clinical and circuit-level phenotype of functional movement disorders. J Neurol Neurosurg Psychiatry 91:814–821. 10.1136/jnnp-2019-322636 PMC7402460 32576619

[72] Myren S, Jungilligens J, Diez I, Bøen E, Elvsåshagen T, Forstmann B, (2026): Ultra-high field brain MRI for functional neurological disorder: Opportunities and challenges. NeuroImage Clin 50:103972. 10.1016/j.nicl.2026.103972 PMC13010964 41846122

[73] Saqib M, Horovitz SG (2024): Harmonization for Parkinson’s disease multi-dataset T1 MRI morphometry classification. NeuroSci 5:600–613. 10.3390/neurosci5040042 PMC11678312 39728674

[74] Yang W, Jin S, Duan W, Yu H, Ping L, Shen Z, (2023): The effects of childhood maltreatment on cortical thickness and gray matter volume: A coordinate-based meta-analysis. Psychol Med 53:1681–1699. 10.1017/S0033291723000661 36946124

[75] Allendorfer JB, Nenert R, Hernando KA, DeWolfe JL, Pati S, Thomas AE, (2019): FMRI response to acute psychological stress differentiates patients with psychogenic non-epileptic seizures from healthy controls – A biochemical and neuroimaging biomarker study. NeuroImage Clin 24:101967. 10.1016/j.nicl.2019.101967 PMC6718876 31446314

[76] Szaflarski JP, Allendorfer JB, Nenert R, LaFrance WC, Barkan HI, DeWolfe J, (2018): Facial emotion processing in patients with seizure disorders. Epilepsy Behav 79:193–204. 10.1016/j.yebeh.2017.12.004 29309953

[77] Hassa T, De Jel E, Tuescher O, Schmidt R, Schoenfeld MA (2016): Functional networks of motor inhibition in conversion disorder patients and feigning subjects. NeuroImage Clin 11:719–727. 10.1016/j.nicl.2016.05.009 PMC4900693 27330971

[78] Hassa T, Sebastian A, Liepert J, Weiller C, Schmidt R, Tüscher O (2017): Symptom-specific amygdala hyperactivity modulates motor control network in conversion disorder. NeuroImage Clin 15:143–150. 10.1016/j.nicl.2017.04.004 PMC5429234 28529870

[79] Hassa T, Spiteri S, Schmidt R, Merkel C, Schoenfeld MA (2021): Increased amygdala activity associated with cognitive reappraisal strategy in functional neurologic disorder. Front Psychiatry 12:613156. 10.3389/fpsyt.2021.613156 PMC8032865 33841199

[80] Maurer CW, LaFaver K, Ameli R, Epstein SA, Hallett M, Horovitz SG (2016): Impaired self-agency in functional movement disorders: A resting-state fMRI study. Neurology 87:564–570. 10.1212/WNL.0000000000002940 PMC4977370 27385746

[81] Waugh RE, Parker JA, Hallett M, Horovitz SG (2023): Classification of functional movement disorders with resting-state functional magnetic resonance imaging. Brain Connect 13:4–14. 10.1089/brain.2022.0001 PMC9942186 35570651

[82] Van Der Kruijs SJM, Jagannathan SR, Bodde NMG, Besseling RMH, Lazeron RHC, Vonck KEJ, (2014): Resting-state networks and dissociation in psychogenic non-epileptic seizures. J Psychiatr Res 54:126–133. 10.1016/j.jpsychires.2014.03.010 24703187

[83] Van Der Kruijs SJM, Bodde NMG, Vaessen MJ, Lazeron RHC, Vonck K, Boon P, (2012): Functional connectivity of dissociation in patients with psychogenic non-epileptic seizures. J Neurol Neurosurg Psychiatry 83:239–247. 10.1136/jnnp-2011-300776 22056967

[84] Marapin RS, Gelauff JM, Marsman JBC, De Jong BM, Dreissen YEM, Koelman JHTM, (2021): Altered posterior midline activity in patients with jerky and tremulous functional movement dsorders. Brain Connect 1:584–593. 10.1089/brain.2020.0779 33724053

[85] Blakemore RL, Sinanaj I, Galli S, Aybek S, Vuilleumier P (2016): Aversive stimuli exacerbate defensive motor behaviour in motor conversion disorder. Neuropsychologia 93:229–241. 10.1016/j.neuropsychologia.2016.11.005 27842291

[86] Wegrzyk J, Kebets V, Richiardi J, Galli S, De Ville DV, Aybek S (2018): Identifying motor functional neurological disorder using resting-state functional connectivity. NeuroImage Clin 17:163–168. 10.1016/j.nicl.2017.10.012 PMC5651543 29071210

[87] Bègue I, Blakemore R, Klug J, Cojan Y, Galli S, Berney A, (2018): Metacognition of visuomotor decisions in conversion disorder. Neuropsychologia 114:251–265. 10.1016/j.neuropsychologia.2018.04.018 29698734

[88] Premi E, Benussi A, Compostella S, Gilberti N, Vergani V, Delrio I, (2017): Multimodal brain analysis of functional neurological disorders: A functional stroke mimic case series. Psychother Psychosom 86:317–319. 10.1159/000465524 28903119

[89] Sojka P, Lošák J, Lamoš M, Bareš M, Kašpárek T, Brázdil M, (2019): Processing of emotions in functional movement disorder: An exploratory fMRI study. Front Neurol 10:861. 10.3389/fneur.2019.00861 PMC6703143 31474926

[90] Jungilligens J, Wellmer J, Kowoll A, Schlegel U, Axmacher N, Popkirov S (2021): Microstructural integrity of affective neurocircuitry in patients with dissociative seizures is associated with emotional task performance, illness severity and trauma history. Seizure 84:91–98. 10.1016/j.seizure.2020.11.021 33307466

