# Supplementary material for: Mega-Analysis of Structural Brain Imaging in Functional Neurological Disorder
Source: Biol Psychiatry. Author manuscript; Available in PMC 2026 Oct 29. (PMC13719111; doi:10.1016/j.biopsych.2026.06.031)
Supplement: Supplementary file 1 [file NIHMS2211682-supplement-Supplemental_Methods.pdf]

SUPPLEMENTARY INFORMATION

Mega-analysis of Structural Brain Imaging in Functional Neurological Disorder

Butler *et al.*

Contents

Note 1: initial systematic search 3

Table S1: list of included studies in initial systematic review. 5

Note 2: Supplementary Methods 7

Note 3: Supplementary Results 9

Table S2: individual region outcomes; FND vs controls, cortical thickness (without Euler covariate). 11

Table S3: individual region outcomes; FND vs controls, cortical surface area (relative), without Euler covariate 14

Table S4: individual region outcomes; FND vs controls, cortical surface area (absolute) 15

Table S5: individual region outcomes; FND vs controls, subcortical volumes. 16

Table S6: individual region outcomes; FND-motor vs controls, cortical thickness, without Euler covariate. 18

Table S7: individual region outcomes; FS vs controls, cortical thickness. 19

Table S8: Breakdown of cases used in the depression subgroup analyses. 20

Table S9: Breakdown of cases used in the anxiety subgroup. 20

Table S10: Breakdown of cases used in the subgroup vs. HC comparisons. 21

Table S11: breakdown of cases in FND-motor vs FND-seiz comparisons. 21

Table S12: breakdown of cases in the sex-matched FND group. 22

Figure S1: Full sample thickness PCA scree plot 23

Figure S2: Full sample area PCA scree plot 24

Figure S3: Anxiety matched thickness PCA scree plot 25

Figure S4: Anxiety matched area PCA scree plot. 26

Figure S5: Depression matched thickness PCA scree plot. 27
Figure S6: Depression matched area PCA scree plot. 28
Figure S8: FMD – FS matched area scree plot. 30
Figure S9: Sex matched PCA thickness scree plot. 31
Figure S10: Sex matched PCA area scree plot. 32
Figure S11: Exploratory PCA – cortical thickness scree plot. 33
Table S13: Leave one out analyses for cortical thickness (main FND cohort). 38
Table S14: Leave one out analyses for subcortical volumes (main FND cohort). 40
Table S15: cortical thickness differences FND vs HCs (with inclusion of Euler as covariate) 44
Table S16: cortical thickness differences FND vs HCs (with inclusion of Euler as covariate) 48
Table S17: cortical thickness differences FND-motor vs HCs (with inclusion of Euler as covariate) 53
Table S18: relative surface area differences in FND-motor vs HCs (with inclusion of Euler as covariate) 57
Table S19: cortical thickness differences in FND-seiz vs HCs (with inclusion of Euler as covariate) 59
Table S20: cortical thickness differences in FND-seiz vs FND-motor (with inclusion of Euler as covariate) 63
Table S21: relative cortical surface area differences in FND-seiz vs FND-motor (with inclusion of Euler as covariate) 66
Table S22: relative cortical surface area differences in FND-seiz vs FND-motor (with inclusion of Euler as covariate) 70
Supplementary References 71

Initial systematic review

An initial systematic search of the literature was conducted to identify suitable studies. Studies were not initially restricted to MRI alone, in case structural MRI data were undertaken as part of another imaging modality.

Systematic search inclusion criteria: neuroimaging (functional or structural) studies of human patient populations of FND with any symptom type aged over 18 (no upper limit to age).

Systematic search exclusion criteria: not English language, case reports, not in humans, were unpublished or grey literature. Excluded diagnoses: somatisation disorder, undifferentiated somatoform disorder, hypochondriasis, somatic symptom disorder, illness anxiety disorder; other FND subtypes outside of FND-motor and FND-seiz (i.e., persistent postural perceptual dizziness, functional vision loss, non-dermatomal somatosensory loss); functional somatic disorders (e.g., fibromyalgia, irritable bowel syndrome).

The following scientific databases were searched: MEDLINE, PsycINFO, Psyndex, and Cochrane Library. See supplementary information for search terms. Searches were run between 13th - 16th March 2020. In total, the searches yielded 32,179 abstracts. After removal of duplicates, 21,109 abstracts remained. After searching through, 677 papers were identified for closer review (101 Inclusions, 171 Conflicts, 405 Maybes). After full text screening, a final total of 80 papers for further review were full-text screened (see *Supplementary Table S1*).

We identified 52 neuroimaging papers that included acquisition of T1-weighted structural MRI scan, as part of a structural or functional data analyses, in both FND patients (FND-motor and FND-seiz) and controls.[18,20–23,25,26,28,29,31,38,40–42,47–85] Several of these papers were re-analyses of the same data.

Search terms

Search terms were as follows: (‘FND’ OR ‘functional neurological disorder’ OR ‘functional neurological symptom disorder’ OR ‘conversion disorder’ OR ‘psychogenic symptoms’ OR ‘pseudoseizure’ OR ‘non-epileptic’ OR ‘dissociative seizures’ OR ‘psychogenic movement’ OR ‘functional movement’ OR ‘functional gait’ OR ‘psychogenic gait’ OR ‘astasia-abasia’ OR ‘astasia abasia’ OR ‘functional tremor’ OR ‘psychogenic tremor’ OR ‘functional dystonia’ OR ‘psychogenic dystonia’ OR ‘fixed dystonia’ OR ‘functional tic’ OR ‘psychogenic tic’ OR ‘functional jerk’ OR ‘psychogenic jerk’ OR ‘functional myoclonus’ OR ‘psychogenic myoclonus’ OR ‘psychogenic movement disorder’ OR ‘functional limb weakness’ OR ‘functional weakness’ OR ‘psychogenic limb weakness’ OR ‘psychogenic weakness’ OR ‘functional paralysis’ OR ‘psychogenic paralysis’ OR ‘hysteri*’ OR ‘non-organic’) AND (“imaging” OR "neuroimaging" OR "neural correlates" OR “magnetic resonance imaging” OR "PET" OR "MRI" OR "fMRI" OR "DAT" OR “neuro imaging” OR “VBM”)

Note 1: initial systematic search

List of studies

Reference	Subtype	N (FND)	Study type	Modality
**Perez et al 2018**	Motor	26	Cross-sectional (comparative)	MRI (struct.)
**Maurer et al 2018**	Motor	48	Cross-sectional (comparative)	MRI (struct.)
**Aybek et al 2014**	Motor	15	Cross-sectional (comparative)	MRI (struct.)
**Williams et al 2018**	Motor	26	Cross-sectional (comparative)	MRI (struct.)
**Ospina et al 2019**	Motor	23	Cross-sectional (comparative)	MRI (struct.)
**Espay et al 2018**	Motor (dystonia)	12	Cross-sectional (comparative)	fMRI
**Nicholson et al 2013**	Motor	15	Cross-sectional (comparative)	fMRI
**Voon et al 2011**	Motor	11	Cross-sectional (comparative)	fMRI
**Blakemore et al 2016**	Motor	10	Cross-sectional (comparative)	fMRI
**Canu et al 2020**	Motor (dystonia)	40	Cross-sectional (comparative)	RS-fMRI
**Diez et al 2019**	Motor	30	Cross-sectional (comparative)	fMRI
**Diez et al 2020**	Motor	30	Cross-sectional (comparative)	fMRI
**Aybek et al 2015**	Motor	12	Cross-sectional (comparative)	fMRI
**Voon et al 2010**	Motor	16	Cross-sectional (comparative)	fMRI
**Espay et al 2017**	Motor (tremor)	27	Cross-sectional (comparative)	fMRI
**Maurer et al 2016**	Motor	35	Cross-sectional (comparative)	fMRI
**Nahab et al 2017**	Motor	14	Cross-sectional (comparative)	fMRI
**Wegrzyk et al 2018**	Motor	23	Cross-sectional (comparative)	fMRI
**Bègue et al 2018**	Motor	10	Cross-sectional (comparative)	fMRI
**Aybek et al 2014**	Motor	12	Cross-sectional (comparative)	fMRI
**Sojka et al 2019**	Motor	15	Cross-sectional (comparative)	fMRI
**Yaźići et al 1998**	Motor (atasia-abasia)	5	Cross-sectional	99mTc-PET
**Czarnecki et al 2011**	Motor (tremor)	5	Cross-sectional (comparative)	SPECT
**Bonilha et al 2007**	Motor (dystonia)	7	Cross-sectional (comparative)	DTI
**Tomic et al 2020**	Motor (dystonia)	43	Cross-sectional (comparative)	DT-MRI
**Burgmer et al 2005**	Paralysis	4	Cross-sectional (comparative)	fMRI
**Van Bielen et al 2011**	Paralysis	9	Cross-sectional (comparative)	fMRI
**De Lange et al 2010**	Paralysis	8	Cross-sectional	fMRI
**Saj et al 2014**	Paralysis	2	Cross-sectional	fMRI
**Stone et al 2007**	Paralysis	4	Cross-sectional (comparative)	fMRI
**Hassa et al 2016**	Paralysis	13	Cross-sectional (comparative)	fMRI
**De Lange et al 2007**	Paralysis	8	Within subjects	fMRI
**Premi et al 2017**	Paralysis (stroke)	4	Cross-sectional (comparative)	fMRI
**Voon et al 2010**	Motor (tremor)	8	Within subjects	fMRI
**Monsa et al 2018**	Paralysis (stroke)	7	Cross-sectional (comparative)	fMRI
**Hassa et al 2017**	Paralysis	13	Cross-sectional (comparative)	fMRI
**Spence et al 2000**	Paralysis	2	Cross-sectional	PET
**Tanaka et al 2007**	Paralysis	4	Cross-sectional	SPECT
**Vuelleumier et al 2001**	Paralysis + sensory loss	7	Cross-sectional	SPECT
**McSweeney et al 2018**	Seizures	20	Cross-sectional (comparative)	MRI (struct.)
**Ristić et al 2015**	Seizures	37	Cross-sectional (comparative)	MRI (struct.)
**Johnstone et al 2016**	Seizures	47	Cross-sectional (comparative)	MRI (struct.)

**Labate et al 2012**	Seizures	20	Cross-sectional (comparative)	MRI (struct.)
**Tatekawa et al 2020**	Seizures	29	Cross-sectional (comparative)	MRI (struct.)
**Vasta et al 2018**	Seizures	21	Cross-sectional (comparative)	MRI (struct.)
**Ding et al 2014**	Seizures	18	Cross-sectional (comparative)	fMRI (FCDM)
**Ding et al 2013**	Seizures	17	Cross-sectional (comparative)	fMRI
**Li et al 2015a**	Seizures	18	Cross-sectional (comparative)	fMRI
**Li et al 2015b**	Seizures	18	Cross-sectional (comparative)	fMRI
**Allendorfer et al 2019**	Seizures	12	Cross-sectional (comparative)	fMRI
**Van der Kruijs**	Seizures	11	Cross-sectional (comparative)	rsfMRI
**van der Kruijs et al 2014**	Seizures	21	Cross-sectional (comparative)	rs-fMRI
**Dienstag et al 2019**	Seizures	9	Cross-sectional (comparative)	fMRI
**Arthuis et al 2015**	Seizures	16	Cross-sectional (comparative)	PET
**Schrag et al 2013**	Motor (dystonia)	6	Cross-sectional (comparative)	PET
**Spanaki et al 1999**	Seizures	11	Cross-sectional	SPECT
**Ettinger 1998**	Seizures	11	Cross-sectional (comparative)	SPECT
**Varma et al 1996**	Seizures	10	Cross-sectional (comparative)	SPECT
**Olver et al 2019**	Seizures	6	Cross-sectional	SPECT
**Gallucci-Neto et al 2020**	Seizures	26	Cross-sectional (comparative)	SPECT
**Neiman et al 2009**	Seizures	13	Cross-sectional	SPECT and fMRI
**Biraben et al 1999**	Seizures	3	Cross-sectional	SPECT
**Hernando et al 2015**	Seizures	8	Cross-sectional (comparative)	DTI
**Lee et al 2015**	Seizures	16	Cross-sectional (comparative)	DTI
**Sone et al 2019**	Seizures	16	Cross-sectional (comparative)	DTI
**Mailis-Gagnon et al 2003**	Sensory	4	Cross-sectional	fMRI
**Burke et al 2014**	Sensory	10	Cross-sectional	fMRI
**Ghaffar et al 2006**	Sensory	3	Cross-sectional	fMRI
**Perez et al 2018**	Mixed	22	Cross-sectional (comparative)	MRI (VBM)
**Perez et al 2017**	Mixed	23	Cross-sectional	MRI (struct.)
**Perez et al 2017**	Mixed	26	Cross-sectional (comparative)	MRI (struct.)
**Kozlowska et al 2017**	Mixed	25	Cross-sectional (comparative)	MRI (struct.)
**Atmaca et al 2016**	Mixed	20	Cross-sectional (comparative)	MRI (struct.)
**Atmaca et al 2006**	Mixed	10	Cross-sectional (comparative)	MRI (struct.)
**Morris et al 2017**	Mixed	25	Cross-sectional (comparative)	fMRI
**Baek et al 2017**	Mixed	26	Cross-sectional (comparative)	fMRI
**Diez et al 2019**	Mixed	32	Cross-sectional (comparative)	DTI
**Kryshtopava et al 2017**	Dysphonia	10	Cross-sectional (comparative)	fMRI
**Brand et al 2009**	Cognitive (amnesia)	14	Cross-sectional	FDG-PET
**Werring et al 2004**	Visual loss	5	Cross-sectional (comparative)	fMRI

Table S1: list of included studies in initial systematic review.

Supplementary Methods

Participants

FND-motor patients were categorised if functional motor symptoms were reported (including functional movement disorder and functional limb weakness regardless of other co-occurring symptoms). FND-seiz patients were classified if functional seizure symptoms were reported. In some cases, patients had both motor and functional symptoms: these were categorised as both FND-motor and FND-seiz and included in both independent analyses. We were not able to definitively establish from the dataset which patients were formally examined or asked about comorbid FND symptoms.

Clinical characteristics

Additionally, caseness via current depression and anxiety scales were established; cutoffs were 14 on the Beck Depression Inventory II,[96] 8 on the Hospital Anxiety and Depression Scale - Anxiety and Depression Subscales,[97] 40 on the State-Trait Anxiety Inventory – Trait Subscale,[98] and 16 on the Beck Anxiety Inventory.[99]

Morphometric extraction

Low quality scans (e.g., with excessive motion) or scans that did not segment well upon troubleshooting were discarded. Data was processed on King's College London HPC Neuroimaging Analysis Network, with the standard recon-all procedure, consisting of motion correction, skull-stripping, affine registration to Talairach atlas, segmentation, smoothing, and parcellation mapping.

To screen for errors in the segmentation process, a Matlab script was be used to identify possible outliers using the read.curv Freesurfer function (https://surfer.nmr.mgh.harvard.edu/). Potential outliers were then manually screened via a slice-by-slice inspection to identify possible errors in the white-grey matter boundary and pial reconstruction steps. We had planned to utilise corrections to the segmentation process via modifying the grey and white matter masks or by adding point sets with Freeview (*FreeSurfer*), depending on which was the most suitable option; however, no scans met these criteria.

Feature selection

We performed a PCA using FactoMineR 2.47.[91] The selection of relevant brain regions to include in the MANCOVAs was based on their contributions to the principal components (PCs), which indicate each variable's importance in defining a particular component.[92] Relevant PCs were identified using a scree plot and eigenvalues >1. The contributions of each variable to the principal components were obtained using the function *get_pca_var(pdp.pca)$contrib*. We retained variables with a contribution coefficient >0.8 to ensure high dimensional influence. While there is no universal cutoff, thresholds above 0.7 for contributions or loadings are frequently used as practical indicators of variable importance in PCA, particularly when interpretability or dimensional reduction is a goal.[93] Additionally, to introduce a dynamic data-driven criterion, we calculated each variable's cumulative contribution across components and retained those above the 70th percentile, a strategy inspired by percentile-based feature selection frameworks.[94,95] The final variable set included those meeting either criterion.

Exploratory PCA

This PCA focussed exclusively on the regions identified as significantly different and FDR corrected in the MANCOVA comparing HC and FND prior to the addition off Euler numbers as covariate (see *Supplementary Table S2*). Thus, while the hypothesis testing was carried out with the MANCOVA, this step uses PCA as an exploratory tool relying on a multivariate approach.[100,101] Unlike the first PCA, which focussed solely on contributions for the 148 variables (regions) and removed those that had a low contribution to the explanation of the variance, this second PCA technique focuses only on the regions which were significantly different in HC vs FND and relies on several different measures (e.g. cos^2, correlation, contribution) to explore the underlying latent factors driving the differences between the groups.

We aimed to summarise the regions based on how strongly they loaded on each dimension, whilst retaining as much variance as possible. The scope was to interpret the results of the MANCOVA by characterizing how the selected brain regions differentiated the groups, and the structure of the group differences identified with the MANCOVA. Regions were grouped based on shared variance to reveal how different brain regions contribute to latent dimensions in a data-driven fashion. We entered data from both hemispheres.

Analyses were conducted with R packages factominer 2.47 and factoextra 1.0.7.[91] The scree plot for the PCA is reported in *Supplementary Fig. 11*) Regions were assigned to each dimension based on their cos^2, for quality of representation of each variable on the dimensions. Cos^2 values were visualised with corrplot(var$cos2, is.corr=FALSE), ensuring that only well-represented variables were considered. Results are presented in *Fig. 5*, created with a custom colour coding based on the components and by overlaying region labels on a brain render.

Statistical analysis of PCAs

To further explore the differences observed, we examined individual contributions for FND and HCs, to identify variables that might be significantly differentially contributing to PC1 and PC2 in each group. To do so, Shapiro-Wilk test was first used to check normality and then t-tests or Wilcoxon tests where appropriate were used to compare dimensions scores. Cohen's d was computed as an effect size measure to quantify differences in PC1 and PC2 between groups.

ComBat harmonisation additional details.

As mentioned in the Methods, we used the ComBat algorithm for cortical thickness harmonisation (1) to control for site effects. ComBat was originally developed for genomic studies combining datasets across batches (2), and has since been successfully adapted to neuroimaging data, including cortical thickness (1), diffusion tensor imaging (3) and more recently EEG (4). All the relevant details regarding how the method was developed for cortical thickness are provided in (1). In brief, the method uses empirical Bayes estimation to model and remove scanner-specific variance using a multiplicative error term. The algorithm also allows to preserve biological variance related to variables such as age, sex, and clinical group (e.g., FND vs. HC), which we explicitly modelled. Harmonisation was performed on parcellated thickness data prior to any statistical analysis. ComBat has also been adopted by the ENIGMA consortium to harmonise different datasets (5).

Note 2: Supplementary Methods

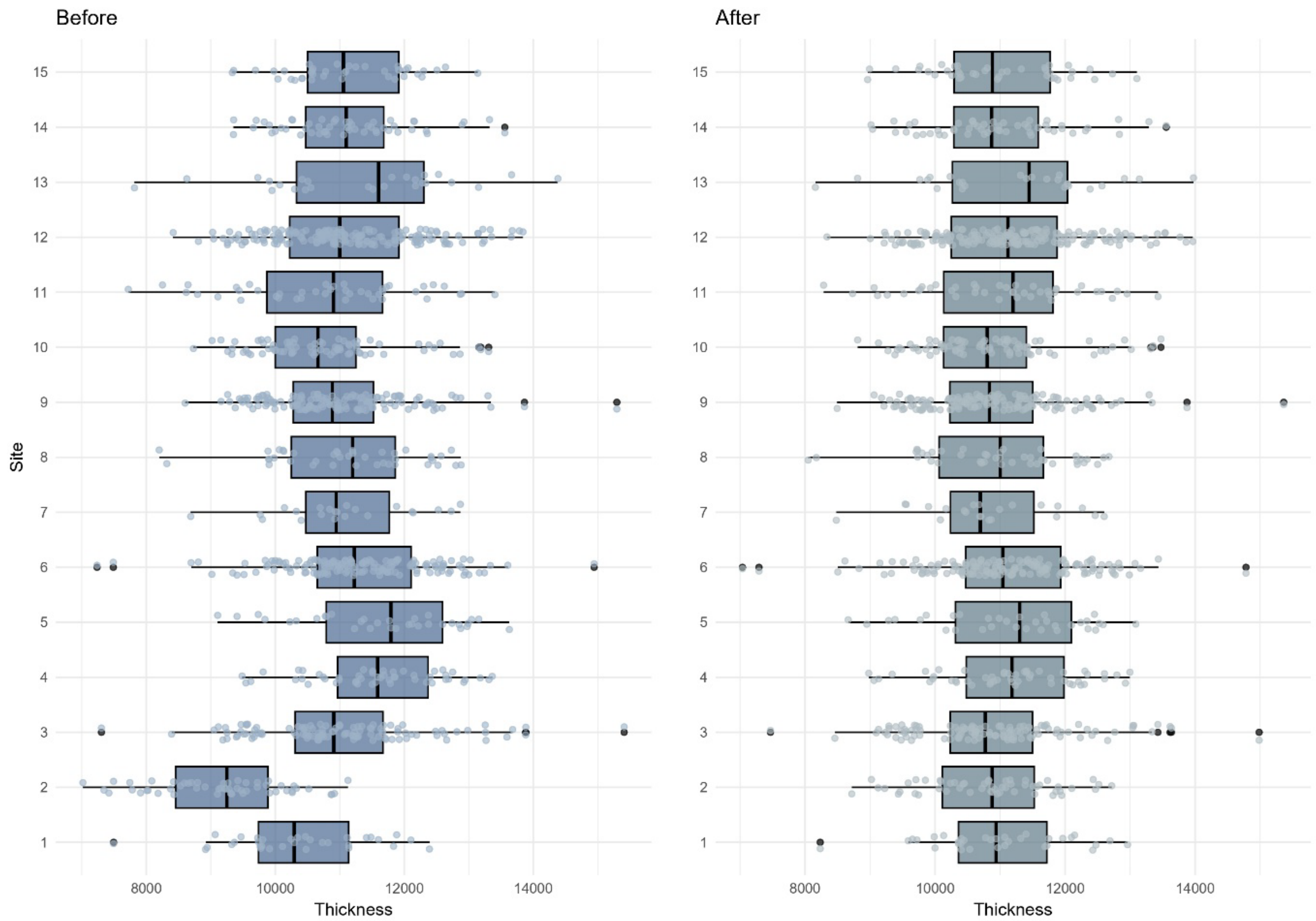

Supplementary Figure A: **Mean cortical thickness before and after harmonisation with ComBat.** Thickness values are on the x-axis; numbers on the y axis refer to different sites. Site 1 = Allendorfer (n = 33); Site 2 = Hassa (n = 54); Site 3 = Horovitz (n = 111); Site 4 = Jansen (n = 51); Site 5 = Tijssen (n = 33); Site 6 = Aybek (n = 150); Site 7 = Premi (n = 22); Site 8 = Ristic (n = 42); Site 9 = Serranova (n = 143); Site 10 = Sojka (n =78); Site 11 = Sone (n = 43); Site 12 = Kerr (n = 178); Site 13 = Nicholson (n = 26); Site 14 = Perez (n = 53); Site 15 = Jungilligens (n = 40).

Supplementary Results

Leave one site out analysis

Thickness

The left precentral gyrus was no longer significant after the removal of Site 12 (Kerr). For all other analyses that were significant in the full analyses, the removal of one site did not alter the significance (see *Supplementary Table S13* for presentation of all regions).

Three regions were significant in 14 of 15 subanalyses, but not in the overall analysis (left central sulcus, left postcentral sulcus, right postcentral gyrus).

Subcortical volumes

The right hippocampus was significant in each subanalysis after the removal of all sites. The right nucleus accumbens was significant in each sub-analysis after the removal of all sites, but not the overall analysis

(see *Supplementary Table S14* for presentation of all regions).

Exploratory PCA

PC1 scores were normally distributed (Shapiro-Wilk $p = 0.1475$). FND had significantly lower PC1 scores than controls ($t = -4.28, p = 0.000021$). The top contributors to this dimension were the bilateral superior frontal gyri (right =.88, $p<.00001$; left =.88, $p <.00001$), and the bilateral superior precentral sulci (right r=.78 $p<.00001$, left r=.77, $p<.00001$). PC1 indicated meaningful group separation (Cohen's $d = 0.285$), whereas PC2 (Cohen's $d= 0.067$) did not.

Note 3: Supplementary Results

Full results from MANCOVAs

The p-values were FDR corrected if the multivariate analyses were significant for main group effect and the analyses were included in the main manuscript. Otherwise, the p-values are not FDR corrected.

	F	P	ηp^2	Observed power	HC-FND thickness difference (mm)
lh_G_cingul.Post.dorsal_thickness	0.299	0.585	0.000	0.085	0.006
lh_G_cuneus_thickness	3.426	0.064	0.004	0.456	0.016
lh_G_front_inf.Opercular_thickness	3.876	0.049	0.004	0.503	.021*
lh_G_front_inf.Orbital_thickness	0.313	0.576	0.000	0.086	0.008
lh_G_front_inf.Triangul_thickness	0.466	0.495	0.001	0.105	0.008
lh_G_front_middle_thickness	2.987	0.084	0.003	0.408	0.016
lh_G_front_sup_thickness	11.783	0.001	0.013	0.929	.034*
lh_G_Ins_lg.S_cent_ins_thickness	0.049	0.824	0.000	0.056	0.004
lh_G_oc.temp_med.Parahip_thickness	0.259	0.611	0.000	0.080	-0.008
lh_G_occipital_middle_thickness	1.523	0.217	0.002	0.234	0.012
lh_G_occipital_sup_thickness	0.009	0.922	0.000	0.051	-0.001
lh_G_orbital_thickness	0.313	0.576	0.000	0.086	0.006
lh_G_pariet_inf.Angular_thickness	0.013	0.909	0.000	0.052	0.001
lh_G_pariet_inf.Supramar_thickness	0.000	0.986	0.000	0.050	0.000
lh_G_parietal_sup_thickness	0.324	0.569	0.000	0.088	-0.005
lh_G_postcentral_thickness	2.614	0.106	0.003	0.365	0.016
lh_G_precentral_thickness	6.631	0.010	0.007	0.730	.032*
lh_G_precuneus_thickness	1.348	0.246	0.001	0.213	0.015
lh_G_rectus_thickness	0.002	0.965	0.000	0.050	-0.001
lh_G_temp_sup.G_T_transv_thickness	0.255	0.614	0.000	0.080	0.007
lh_G_temp_sup.Lateral_thickness	1.772	0.184	0.002	0.265	0.018
lh_G_temporal_middle_thickness	0.442	0.506	0.000	0.102	0.008
lh_G.S_cingul.Ant_thickness	0.044	0.833	0.000	0.055	0.002
lh_G.S_cingul.Mid.Ant_thickness	1.738	0.188	0.002	0.261	0.014
lh_G.S_cingul.Mid.Post_thickness	1.556	0.213	0.002	0.238	0.011
lh_G.S_frontomargin_thickness	0.667	0.414	0.001	0.129	0.009
lh_G.S_paracentral_thickness	7.820	0.005	0.008	0.798	.032*
lh_G.S_subcentral_thickness	0.004	0.950	0.000	0.050	0.001
lh_Lat_Fis.post_thickness	3.435	0.064	0.004	0.457	0.019
lh_Pole_occipital_thickness	2.832	0.093	0.003	0.390	-0.017
lh_Pole_temporal_thickness	1.266	0.261	0.001	0.203	0.016
lh_S_central_thickness	5.617	0.018	0.006	0.658	.019*
lh_S_circular_insula_ant_thickness	0.673	0.412	0.001	0.130	0.011
lh_S_circular_insula_inf_thickness	0.538	0.463	0.001	0.113	0.009
lh_S_circular_insula_sup_thickness	0.036	0.850	0.000	0.054	0.002
lh_S_front_inf_thickness	1.399	0.237	0.002	0.219	0.010
lh_S_front_middle_thickness	2.199	0.138	0.002	0.316	0.014
lh_S_front_sup_thickness	11.327	0.001	0.012	0.920	.028*
lh_S_intrapariet.P_trans_thickness	0.203	0.652	0.000	0.074	0.004
lh_S_oc_middle.Lunatus_thickness	0.884	0.347	0.001	0.156	0.010
lh_S_oc_sup.transversal_thickness	0.073	0.788	0.000	0.058	0.002
lh_S_oc.temp_med.Lingual_thickness	1.106	0.293	0.001	0.183	0.010
lh_S_occipital_ant_thickness	0.006	0.938	0.000	0.051	-0.001
lh_S_orbital_med.olfact_thickness	0.171	0.679	0.000	0.070	0.005
lh_S_orbital.H_Shaped_thickness	1.099	0.295	0.001	0.182	0.012
lh_S_parieto_occipital_thickness	0.485	0.486	0.001	0.107	0.006
lh_S_postcentral_thickness	6.319	0.012	0.007	0.709	.021*
lh_S_precentral.inf.part_thickness	2.088	0.149	0.002	0.303	0.013
lh_S_precentral.sup.part_thickness	10.835	0.001	0.012	0.908	.034*
lh_S_suborbital_thickness	1.430	0.232	0.002	0.223	0.019
lh_S_temporal_sup_thickness	0.030	0.862	0.000	0.053	-0.001

rh_G_cuneus_thickness	10.273	0.001	0.011	0.893	.027*
rh_G_front_inf.Opercular_thickness	10.339	0.001	0.011	0.895	.033*
rh_G_front_inf.Triangul_thickness	0.171	0.679	0.000	0.070	0.005
rh_G_front_middle_thickness	4.877	0.027	0.005	0.597	.020*
rh_G_front_sup_thickness	10.841	0.001	0.012	0.908	.032*
rh_G_oc.temp_med.Lingual_thickness	0.856	0.355	0.001	0.152	0.008
rh_G_oc.temp_med.Parahip_thickness	0.017	0.896	0.000	0.052	0.002
rh_G_occipital_middle_thickness	1.047	0.307	0.001	0.176	0.010
rh_G_occipital_sup_thickness	1.587	0.208	0.002	0.242	0.014
rh_G_orbital_thickness	2.211	0.137	0.002	0.318	0.015
rh_G_pariet_inf.Angular_thickness	0.046	0.830	0.000	0.055	-0.002
rh_G_pariet_inf.Supramar_thickness	0.971	0.325	0.001	0.166	0.009
rh_G_parietal_sup_thickness	1.241	0.266	0.001	0.200	-0.011
rh_G_postcentral_thickness	4.683	0.031	0.005	0.580	.023*
rh_G_precentral_thickness	16.649	0.000	0.018	0.983	.051*
rh_G_precuneus_thickness	0.000	0.987	0.000	0.050	0.000
rh_G_rectus_thickness	1.264	0.261	0.001	0.202	0.014
rh_G_temp_sup.G_T_transv_thickness	0.062	0.804	0.000	0.057	0.004
rh_G_temp_sup.Lateral_thickness	1.108	0.293	0.001	0.183	0.013
rh_G_temp_sup.Plan_polar_thickness	1.948	0.163	0.002	0.286	0.024
rh_G_temp_sup.Plan_tempo_thickness	1.215	0.271	0.001	0.196	0.014
rh_G_temporal_middle_thickness	0.158	0.691	0.000	0.068	-0.005
rh_G.S_cingul.Ant_thickness	0.050	0.823	0.000	0.056	-0.002
rh_G.S_cingul.Mid.Ant_thickness	4.419	0.036	0.005	0.556	.021*
rh_G.S_cingul.Mid.Post_thickness	1.401	0.237	0.002	0.219	0.011
rh_G.S_frontomargin_thickness	3.882	0.049	0.004	0.503	-.023*
rh_G.S_paracentral_thickness	12.235	0.000	0.013	0.938	.040*
rh_G.S_subcentral_thickness	0.019	0.891	0.000	0.052	0.001
rh_G.S_transv_frontopol_thickness	0.118	0.731	0.000	0.064	-0.004
rh_Lat_Fis.post_thickness	1.689	0.194	0.002	0.255	0.013
rh_Pole_occipital_thickness	0.568	0.451	0.001	0.117	-0.007
rh_Pole_temporal_thickness	0.457	0.499	0.000	0.104	0.009
rh_S_calcarine_thickness	1.942	0.164	0.002	0.286	0.013
rh_S_central_thickness	5.207	0.023	0.006	0.625	.018*
rh_S_cingul.Marginalis_thickness	1.128	0.289	0.001	0.186	0.010
rh_S_circular_insula_ant_thickness	0.324	0.569	0.000	0.088	-0.008
rh_S_circular_insula_sup_thickness	0.535	0.465	0.001	0.113	0.006
rh_S_front_inf_thickness	0.181	0.671	0.000	0.071	0.004
rh_S_front_middle_thickness	0.786	0.376	0.001	0.144	0.008
rh_S_front_sup_thickness	12.234	0.000	0.013	0.937	.030*
rh_S_intrapariet.P_trans_thickness	0.139	0.709	0.000	0.066	0.003
rh_S_oc_middle.Lunatus_thickness	0.021	0.885	0.000	0.052	0.002
rh_S_oc_sup.transversal_thickness	0.828	0.363	0.001	0.149	0.008
rh_S_occipital_ant_thickness	0.127	0.722	0.000	0.065	0.004
rh_S_orbital_med.olfact_thickness	0.635	0.426	0.001	0.125	0.010
rh_S_orbital.H_Shaped_thickness	2.574	0.109	0.003	0.361	0.019
rh_S_parieto_occipital_thickness	3.649	0.056	0.004	0.479	0.018
rh_S_postcentral_thickness	1.498	0.221	0.002	0.231	0.010
rh_S_precentral.inf.part_thickness	4.199	0.041	0.005	0.535	.019*
rh_S_precentral.sup.part_thickness	15.220	0.000	0.016	0.974	.044*
rh_S_temporal_sup_thickness	2.483	0.115	0.003	0.350	-0.012

Table S2: individual region outcomes; FND vs controls, cortical thickness (without Euler covariate).

Dependent Variable	F	Sig.	Partial Eta Squared	Observed Power	mean difference (I-J)
lh_G_cingul.Post.ventral_area	4.824034	0.264261	0.005205	0.592569	-0.00002681
lh_G_cuneus_area	2.050019	0.527831	0.002219	0.298521	9.82527E-05
lh_G_front_inf.Triangul_area	0.052881	0.935058	5.74E-05	0.056067	1.19419E-05
lh_G_front_middle_area	0.039498	0.93574	4.28E-05	0.054527	2.38315E-05
lh_G_front_sup_area	1.543011	0.643458	0.001671	0.236732	-0.000182367
lh_G_Ins_lg.S_cent_ins_area	2.95371	0.375344	0.003193	0.404075	-3.7623E-05
lh_G_insular_short_area	0.033914	0.93574	3.68E-05	0.053886	4.23201E-06
lh_G_oc.temp_med.Lingual_area	0.185935	0.846537	0.000202	0.071514	4.4847E-05
lh_G_occipital_middle_area	0.650103	0.747171	0.000705	0.126987	6.12986E-05
lh_G_occipital_sup_area	1.006016	0.724783	0.00109	0.170559	4.70919E-05
lh_G_pariet_inf.Angular_area	0.687984	0.737326	0.000746	0.131595	6.93941E-05
lh_G_pariet_inf.Supramar_area	0.040667	0.93574	4.41E-05	0.054662	-2.11728E-05
lh_G_parietal_sup_area	2.47E-06	0.998747	2.68E-09	0.05	1.56072E-07
lh_G_postcentral_area	11.48176	0.064129	0.0123	0.922918	0
lh_G_precentral_area	0.032139	0.93574	3.49E-05	0.053682	-1.1399E-05
lh_G_precuneus_area	3.785286	0.331848	0.004089	0.493501	-0.000158765
lh_G_temp_sup.G_T_transv_area	0.08237	0.904747	8.93E-05	0.059469	7.07816E-06
lh_G_temp_sup.Plan_tempo_area	2.186931	0.51518	0.002366	0.314956	-7.27081E-05
lh_G_temporal_inf_area	4.710038	0.264261	0.005083	0.582418	0
lh_G_temporal_middle_area	0.446894	0.800551	0.000484	0.102448	4.81324E-05
lh_G.S_cingul.Ant_area	0.022341	0.943745	2.42E-05	0.052558	8.35523E-06
lh_G.S_cingul.Mid.Ant_area	1.578405	0.643458	0.001709	0.24108	-5.18978E-05
lh_G.S_cingul.Mid.Post_area	4.335648	0.280347	0.00468	0.54782	-0.00007565
lh_G.S_occipital_inf_area	0.276092	0.833948	0.000299	0.082114	3.46697E-05
lh_G.S_paracentral_area	1.373959	0.6817	0.001488	0.21592	-4.64998E-05
lh_Lat_Fis.post_area	6.259414	0.200413	0.006743	0.705168	-0.00008122
lh_Pole_occipital_area	0.021018	0.943745	2.28E-05	0.052406	-8.81993E-06
lh_S_calcarine_area	0.35711	0.825394	0.000387	0.091721	-6.54323E-05
lh_S_central_area	0.154443	0.846537	0.000167	0.067836	-2.80076E-05
lh_S_cingul.Marginalis_area	3.639519	0.331848	0.003932	0.478444	-6.11913E-05
lh_S_circular_insula_ant_area	0.41563	0.800551	0.000451	0.098704	1.21286E-05
lh_S_circular_insula_inf_area	4.707852	0.264261	0.00508	0.582222	-0.00007445
lh_S_circular_insula_sup_area	5.980822	0.200892	0.006445	0.685479	-0.00008705
lh_S_front_inf_area	1.001989	0.724783	0.001086	0.170063	-7.92292E-05
lh_S_front_middle_area	0.009975	0.971039	1.08E-05	0.051141	-6.07821E-06
lh_S_front_sup_area	0.222752	0.846537	0.000242	0.07583	-4.3572E-05
lh_S_intrapariet.P_trans_area	0.153565	0.846537	0.000167	0.067733	-4.00366E-05
lh_S_oc_middle.Lunatus_area	1.472913	0.65512	0.001595	0.228109	7.27118E-05
lh_S_oc.temp_lat_area	1.60919	0.643458	0.001742	0.244859	5.63621E-05
lh_S_orbital_lateral_area	3.749211	0.331848	0.00405	0.4898	4.46067E-05
lh_S_parieto_occipital_area	0.002951	0.978647	3.2E-06	0.050337	4.18305E-06
lh_S_pericallosal_area	1.072097	0.724783	0.001161	0.178697	5.58081E-05

lh_S_postcentral_area	0.712084	0.737326	0.000772	0.134531	8.2868E-05
lh_S_precentral.sup.part_area	0.58971	0.772761	0.000639	0.119661	-4.39308E-05
lh_S_subparietal_area	0.198877	0.846537	0.000216	0.073029	-2.13808E-05
lh_S_temporal_inf_area	1.234736	0.69218	0.001337	0.198751	7.41812E-05
lh_S_temporal_sup_area	0.416845	0.800551	0.000452	0.09885	9.61114E-05
lh_S_temporal_transverse_area	2.573584	0.454972	0.002784	0.360582	3.90063E-05
rh_G_cingul.Post.ventral_area	3.404299	0.331848	0.003679	0.453576	-2.36337E-05
rh_G_cuneus_area	2.03601	0.527831	0.002203	0.296832	-9.93604E-05
rh_G_front_inf.Orbital_area	0.752693	0.737326	0.000816	0.139485	-1.77243E-05
rh_G_front_inf.Triangul_area	0.299474	0.829864	0.000325	0.084879	-2.77606E-05
rh_G_front_middle_area	0.181476	0.846537	0.000197	0.070992	5.18518E-05
rh_G_front_sup_area	6.315175	0.200413	0.006803	0.708988	0
rh_G_oc.temp_med.Lingual_area	0.239249	0.845551	0.000259	0.07777	-4.87341E-05
rh_G_occipital_middle_area	0.905524	0.734794	0.000981	0.158204	7.91451E-05
rh_G_occipital_sup_area	0.000812	0.987562	8.81E-07	0.050093	-1.48684E-06
rh_G_pariet_inf.Angular_area	0.923136	0.734794	0.001	0.160367	9.85755E-05
rh_G_pariet_inf.Supramar_area	0.238572	0.845551	0.000259	0.077691	4.02983E-05
rh_G_parietal_sup_area	3.47026	0.331848	0.00375	0.460619	0.000154115
rh_G_postcentral_area	0.716381	0.737326	0.000776	0.135055	4.62669E-05
rh_G_precentral_area	3.395932	0.331848	0.00367	0.452679	-0.000112801
rh_G_precuneus_area	0.453651	0.800551	0.000492	0.103259	-5.03008E-05
rh_G_temp_sup.G_T_transv_area	0.31636	0.829864	0.000343	0.08688	9.21996E-06
rh_G_temp_sup.Lateral_area	0.403662	0.800551	0.000438	0.097273	-2.98465E-05
rh_G_temp_sup.Plan_tempo_area	1.254087	0.69218	0.001358	0.201138	-3.52615E-05
rh_G_temporal_inf_area	3.009045	0.375344	0.003253	0.41028	0.000137308
rh_G_temporal_middle_area	0.412087	0.800551	0.000447	0.09828	4.5083E-05
rh_G.S_cingul.Ant_area	0.894775	0.734794	0.00097	0.156884	5.90546E-05
rh_G.S_cingul.Mid.Ant_area	0.293967	0.829864	0.000319	0.084228	2.14329E-05
rh_G.S_cingul.Mid.Post_area	4.31907	0.280347	0.004663	0.546244	-0.00007778
rh_G.S_occipital_inf_area	0.531729	0.798971	0.000576	0.112653	3.31404E-05
rh_G.S_paracentral_area	9.600647	0.064129	0.010306	0.871875	0
rh_G.S_transv_frontopol_area	1.664541	0.643458	0.001802	0.251646	4.76884E-05
rh_Lat_Fis.post_area	9.965566	0.064129	0.010693	0.883678	0
rh_Pole_occipital_area	0.002741	0.978647	2.97E-06	0.050313	-5.00231E-06
rh_S_calcarine_area	0.76668	0.737326	0.000831	0.141194	-8.72756E-05
rh_S_central_area	0.099837	0.899068	0.000108	0.06149	-2.30253E-05
rh_S_cingul.Marginalis_area	7.723833	0.133452	0.008308	0.792842	0
rh_S_circular_insula_ant_area	0.31399	0.829864	0.00034	0.086599	1.27953E-05
rh_S_circular_insula_inf_area	5.393972	0.24508	0.005816	0.640626	-0.00006606
rh_S_circular_insula_sup_area	2.34096	0.50542	0.002533	0.333282	-5.1053E-05
rh_S_collat_transv_post_area	2.264424	0.509637	0.00245	0.324199	-4.13218E-05
rh_S_front_inf_area	0.701668	0.737326	0.00076	0.133261	6.54766E-05
rh_S_front_middle_area	0.094511	0.899068	0.000102	0.060873	2.96626E-05

rh_S_front_sup_area	2.958661	0.375344	0.003199	0.404632	-0.000157018
rh_S_intrapariet.P_trans_area	0.07645	0.904747	8.29E-05	0.058785	3.1133E-05
rh_S_oc_middle.Lunatus_area	1.27947	0.69218	0.001386	0.204269	6.34517E-05
rh_S_occipital_ant_area	0.179417	0.846537	0.000195	0.070751	-1.75023E-05
rh_S_orbital_lateral_area	0.152097	0.846537	0.000165	0.067562	1.04018E-05
rh_S_postcentral_area	1.013848	0.724783	0.001098	0.171523	8.79441E-05
rh_S_precentral.sup.part_area	1.012428	0.724783	0.001097	0.171348	-6.05341E-05
rh_S_subparietal_area	0.006111	0.978476	6.63E-06	0.050699	4.442E-06
rh_S_temporal_inf_area	0.76059	0.737326	0.000824	0.14045	4.5902E-05
rh_S_temporal_sup_area	0.464931	0.800551	0.000504	0.104613	-0.000102331
rh_S_temporal_transverse_area	0.696055	0.737326	0.000754	0.132578	1.49643E-05

Table S3: individual region outcomes; FND vs controls, cortical surface area (relative), without Euler covariate

	F	P	ηp^2	Observed power	HC-FND surface area difference (mm^2)
brain_region_lh_G_cingul.Post.dorsal_area	8.329	0.004	0.009	0.822	13.414*
brain_region_lh_G_cingul.Post.ventral_area	0.271	0.603	0.000	0.082	1.140
brain_region_lh_G_cuneus_area	14.544	0.000	0.016	0.968	50.907*
brain_region_lh_G_front_middle_area	7.342	0.007	0.008	0.772	77.311*
brain_region_lh_G_front_sup_area	6.186	0.013	0.007	0.700	89.622*
brain_region_lh_G_Ins_lg.S_cent_ins_area	1.095	0.296	0.001	0.182	4.398
brain_region_lh_G_insular_short_area	7.698	0.006	0.008	0.792	12.961*
brain_region_lh_G_oc.temp_med.Lingual_area	8.487	0.004	0.009	0.829	58.540*
brain_region_lh_G_orbital_area	7.725	0.006	0.008	0.793	32.652*
brain_region_lh_G_pariet_inf.Supramar_area	4.050	0.044	0.004	0.520	41.466*
brain_region_lh_G_postcentral_area	30.377	0.000	0.032	1.000	71.317*
brain_region_lh_G_precentral_area	8.270	0.004	0.009	0.819	39.028*
brain_region_lh_G_temp_sup.Lateral_area	15.594	0.000	0.017	0.976	41.224*
brain_region_lh_G_temporal_inf_area	18.435	0.000	0.020	0.990	75.477*
brain_region_lh_G_temporal_middle_area	10.976	0.001	0.012	0.911	52.578*
brain_region_lh_G.S_cingul.Ant_area	8.558	0.004	0.009	0.832	38.894*
brain_region_lh_G.S_cingul.Mid.Ant_area	2.238	0.135	0.002	0.321	13.007
brain_region_lh_G.S_cingul.Mid.Post_area	1.650	0.199	0.002	0.250	9.715
brain_region_lh_G.S_subcentral_area	4.262	0.039	0.005	0.541	18.059*
brain_region_lh_Lat_Fis.post_area	1.066	0.302	0.001	0.178	6.166
brain_region_lh_Pole_occipital_area	7.648	0.006	0.008	0.789	32.378*
brain_region_lh_Pole_temporal_area	11.459	0.001	0.012	0.922	28.769*
brain_region_lh_S_calcarine_area	2.685	0.102	0.003	0.374	32.758
brain_region_lh_S_circular_insula_sup_area	3.773	0.052	0.004	0.492	14.757
brain_region_lh_S_collat_transv_post_area	5.414	0.020	0.006	0.642	10.715*
brain_region_lh_S_front_sup_area	4.728	0.030	0.005	0.584	42.227*
brain_region_lh_S_oc_middle.Lunatus_area	8.467	0.004	0.009	0.828	31.351*
brain_region_lh_S_oc.temp_med.Lingual_area	13.699	0.000	0.015	0.959	43.052*
brain_region_lh_S_orbital.H_Shaped_area	11.233	0.001	0.012	0.917	29.496*
brain_region_lh_S_temporal_sup_area	13.306	0.000	0.014	0.954	118.789*
brain_region_lh_S_temporal_transverse_area	9.861	0.002	0.011	0.880	13.731*
brain_region_rh_G_cingul.Post.ventral_area	0.312	0.576	0.000	0.086	1.332
brain_region_rh_G_cuneus_area	2.179	0.140	0.002	0.314	20.694
brain_region_rh_G_front_middle_area	8.607	0.003	0.009	0.834	79.127*
brain_region_rh_G_front_sup_area	1.942	0.164	0.002	0.285	47.223
brain_region_rh_G_oc.temp_med.Lingual_area	4.231	0.040	0.005	0.538	40.174*
brain_region_rh_G_occipital_middle_area	9.841	0.002	0.011	0.880	53.247*
brain_region_rh_G_orbital_area	12.917	0.000	0.014	0.948	48.402*
brain_region_rh_G_pariet_inf.Supramar_area	8.275	0.004	0.009	0.820	48.580*
brain_region_rh_G_postcentral_area	12.511	0.000	0.013	0.942	40.571*
brain_region_rh_G_precentral_area	2.925	0.088	0.003	0.401	22.359
brain_region_rh_G_temp_sup.Lateral_area	6.825	0.009	0.007	0.742	26.113*
brain_region_rh_G_temp_sup.Plan_tempo_area	1.926	0.166	0.002	0.284	8.591
brain_region_rh_G_temporal_inf_area	15.024	0.000	0.016	0.972	65.232*
brain_region_rh_G_temporal_middle_area	12.679	0.000	0.014	0.945	57.039*
brain_region_rh_G.S_cingul.Ant_area	13.103	0.000	0.014	0.951	59.048*
brain_region_rh_G.S_cingul.Mid.Ant_area	9.840	0.002	0.011	0.880	27.819*
brain_region_rh_G.S_cingul.Mid.Post_area	2.202	0.138	0.002	0.317	12.706
brain_region_rh_G.S_subcentral_area	10.216	0.001	0.011	0.891	28.888*
brain_region_rh_Lat_Fis.post_area	1.349	0.246	0.001	0.213	7.017
brain_region_rh_Pole_occipital_area	8.482	0.004	0.009	0.829	55.308*
brain_region_rh_Pole_temporal_area	11.223	0.001	0.012	0.917	29.145*
brain_region_rh_S_calcarine_area	2.054	0.152	0.002	0.299	26.915
brain_region_rh_S_circular_insula_sup_area	4.440	0.035	0.005	0.558	14.632*
brain_region_rh_S_collat_transv_post_area	0.044	0.834	0.000	0.055	1.013
brain_region_rh_S_intrapariet.P_trans_area	7.754	0.005	0.008	0.794	60.919*
brain_region_rh_S_oc_middle.Lunatus_area	7.393	0.007	0.008	0.775	28.409*
brain_region_rh_S_orbital.H_Shaped_area	13.474	0.000	0.014	0.956	31.821*
brain_region_rh_S_temporal_sup_area	7.475	0.006	0.008	0.780	88.085*
brain_region_rh_S_temporal_transverse_area	6.098	0.014	0.007	0.694	8.069*

Table S4: individual region outcomes; FND vs controls, cortical surface area (absolute)

	F	P	ηp^2	Observed power	HC-FND volume difference (mm^3)
Left.Cerebellum.White.Matter	0.054	0.816	0.000	0.056	-30.114
Left.Cerebellum.Cortex	2.153	0.143	0.002	0.311	502.248
Left.Thalamus.Proper	1.405	0.236	0.002	0.220	47.639
Left.Caudate	3.307	0.069	0.004	0.443	44.637
Left.Putamen	3.621	0.057	0.004	0.477	62.550
Left.Pallidum	5.771	0.016	0.006	0.670	32.680*
Left.Hippocampus	5.626	0.018	0.006	0.659	51.709*
Left.Amygdala	0.816	0.367	0.001	0.147	10.383
Left.Accumbens.area	0.467	0.494	0.001	0.105	3.957
Left.VentralDC	0.122	0.727	0.000	0.064	-8.582
Right.Cerebellum.White.Matter	3.953	0.047	0.004	0.510	-274.897*
Right.Cerebellum.Cortex	1.967	0.161	0.002	0.288	491.932
Right.Thalamus.Proper	3.981	0.046	0.004	0.513	72.861*
Right.Caudate	2.774	0.096	0.003	0.384	41.144
Right.Putamen	3.630	0.057	0.004	0.477	61.653
Right.Pallidum	1.345	0.246	0.001	0.212	15.654
Right.Hippocampus	11.055	0.001	0.012	0.913	75.415*
Right.Amygdala	0.637	0.425	0.001	0.125	8.805
Right.Accumbens.area	7.097	0.008	0.008	0.758	14.975*
Right.VentralDC	1.041	0.308	0.001	0.175	22.540

Table S5: individual region outcomes; FND vs controls, subcortical volumes.

	F	P	ηp^2	Observed power	HC-FND thickness difference (mm)
lh_G_cingul.Post.dorsal_thickness	1.086	0.5508	0.002	0.180	0.014
lh_G_cingul.Post.ventral_thickness	0.129	0.884	0.000	0.065	-0.008
lh_G_cuneus_thickness	3.985	0.2114	0.006	0.513	.022*
lh_G_front_inf.Opercular_thickness	4.914	0.1695	0.008	0.600	.030*
lh_G_front_inf.Orbital_thickness	0.582	0.6998	0.001	0.119	0.013
lh_G_front_inf.Triangul_thickness	0.506	0.7191	0.001	0.109	0.010
lh_G_front_middle_thickness	3.906	0.2114	0.006	0.505	.023*
lh_G_front_sup_thickness	8.408	0.0486	0.014	0.825	.035*
lh_G_Ins_lg.S_cent_ins_thickness	2.249	0.3096	0.004	0.322	0.031
lh_G_insular_short_thickness	0.013	0.9593	0.000	0.052	-0.002
lh_G_oc.temp_med.Lingual_thickness	2.760	0.2834	0.005	0.382	0.017
lh_G_oc.temp_med.Parahip_thickness	0.186	0.8559	0.000	0.071	0.009
lh_G_occipital_middle_thickness	3.210	0.2543	0.005	0.432	0.021
lh_G_occipital_sup_thickness	0.000	0.9868	0.000	0.050	0.000
lh_G_orbital_thickness	0.930	0.5917	0.002	0.161	0.012
lh_G_pariet_inf.Angular_thickness	0.241	0.8559	0.000	0.078	0.006
lh_G_pariet_inf.Supramar_thickness	0.272	0.8511	0.000	0.082	0.006
lh_G_parietal_sup_thickness	0.283	0.8505	0.000	0.083	0.006
lh_G_postcentral_thickness	2.100	0.3211	0.003	0.304	0.018
lh_G_precentral_thickness	1.957	0.3461	0.003	0.287	0.022
lh_G_precuneus_thickness	0.948	0.5917	0.002	0.163	0.015
lh_G_rectus_thickness	0.147	0.8717	0.000	0.067	0.005
lh_G_temp_sup.G_T_transv_thickness	1.112	0.5501	0.002	0.183	-0.019
lh_G_temp_sup.Lateral_thickness	0.003	0.9718	0.000	0.050	0.001
lh_G_temp_sup.Plan_polar_thickness	3.287	0.2543	0.005	0.441	0.041
lh_G_temp_sup.Plan_tempo_thickness	0.040	0.9222	0.000	0.055	0.003
lh_G_temporal_inf_thickness	0.699	0.6441	0.001	0.133	0.012
lh_G_temporal_middle_thickness	0.078	0.9087	0.000	0.059	0.004
lh_G.S_cingul.Ant_thickness	0.860	0.6158	0.001	0.152	0.011
lh_G.S_cingul.Mid.Post_thickness	1.285	0.5194	0.002	0.205	0.013
lh_G.S_subcentral_thickness	0.527	0.7148	0.001	0.112	-0.010
lh_Lat_Fis.ant.Horizont_thickness	0.019	0.9524	0.000	0.052	0.003
lh_Lat_Fis.post_thickness	5.254	0.1555	0.009	0.629	.028*
lh_Pole_occipital_thickness	0.003	0.9718	0.000	0.050	-0.001
lh_Pole_temporal_thickness	2.639	0.2834	0.004	0.368	0.029
lh_S_central_thickness	2.817	0.2834	0.005	0.388	0.017
lh_S_circular_insula_ant_thickness	4.807	0.1708	0.008	0.591	.037*
lh_S_circular_insula_inf_thickness	0.000	0.9868	0.000	0.050	0.000
lh_S_circular_insula_sup_thickness	0.382	0.7874	0.001	0.095	0.006
lh_S_collat_transv_post_thickness	0.052	0.9087	0.000	0.056	-0.004
lh_S_front_inf_thickness	2.505	0.2869	0.004	0.352	0.017
lh_S_front_middle_thickness	1.666	0.4053	0.003	0.252	0.015
lh_S_front_sup_thickness	9.420	0.0362	0.015	0.865	.031*
lh_S_intrapariet.P_trans_thickness	0.766	0.6385	0.001	0.141	0.008
lh_S_oc_middle.Lunatus_thickness	1.213	0.5282	0.002	0.196	0.014
lh_S_oc_sup.transversal_thickness	0.018	0.9524	0.000	0.052	0.002
lh_S_oc.temp_med.Lingual_thickness	2.799	0.2834	0.005	0.386	0.020
lh_S_occipital_ant_thickness	0.023	0.9524	0.000	0.053	0.002
lh_S_orbital_med.olfact_thickness	4.700	0.1726	0.008	0.581	.030*
lh_S_orbital.H_Shaped_thickness	3.197	0.2543	0.005	0.431	0.025
lh_S_parieto_occipital_thickness	0.094	0.9037	0.000	0.061	0.003
lh_S_postcentral_thickness	14.794	0.0107	0.024	0.970	.039*
lh_S_precentral.inf.part_thickness	5.165	0.1555	0.008	0.621	.026*
lh_S_precentral.sup.part_thickness	13.480	0.0107	0.022	0.956	.045*
lh_S_suborbital_thickness	2.376	0.3023	0.004	0.337	0.031
lh_S_temporal_inf_thickness	0.186	0.8559	0.000	0.071	0.006
lh_S_temporal_sup_thickness	0.005	0.9718	0.000	0.051	0.001
rh_G_cingul.Post.dorsal_thickness	2.318	0.3023	0.004	0.330	0.021
rh_G_cuneus_thickness	7.515	0.0712	0.012	0.781	.029*
rh_G_front_inf.Opercular_thickness	6.506	0.1036	0.011	0.721	.033*
rh_G_front_inf.Triangul_thickness	0.200	0.8559	0.000	0.073	0.006
rh_G_front_middle_thickness	3.567	0.2349	0.006	0.470	0.021
rh_G_front_sup_thickness	10.851	0.0199	0.017	0.908	.039*
rh_G_insular_short_thickness	1.066	0.5508	0.002	0.178	-0.022
rh_G_oc.temp_med.Lingual_thickness	0.780	0.6385	0.001	0.143	0.010
rh_G_oc.temp_med.Parahip_thickness	2.687	0.2834	0.004	0.373	0.033
rh_G_occipital_middle_thickness	0.440	0.7544	0.001	0.102	0.008
rh_G_occipital_sup_thickness	3.040	0.2716	0.005	0.413	0.025
rh_G_orbital_thickness	2.141	0.3189	0.003	0.309	0.018
rh_G_pariet_inf.Angular_thickness	2.348	0.3023	0.004	0.334	0.019
rh_G_pariet_inf.Supramar_thickness	2.735	0.2834	0.004	0.379	0.020
rh_G_parietal_sup_thickness	0.068	0.9087	0.000	0.058	0.003
rh_G_postcentral_thickness	3.543	0.2349	0.006	0.468	0.024
rh_G_precentral_thickness	11.753	0.0183	0.019	0.928	.054*
rh_G_precuneus_thickness	0.695	0.6441	0.001	0.132	0.010
rh_G_rectus_thickness	2.501	0.2869	0.004	0.352	0.025
rh_G_temp_sup.G_T_transv_thickness	0.065	0.9087	0.000	0.057	-0.005
rh_G_temp_sup.Lateral_thickness	0.253	0.8559	0.000	0.079	0.008
rh_G_temp_sup.Plan_polar_thickness	0.536	0.7148	0.001	0.113	0.016
rh_G_temp_sup.Plan_tempo_thickness	1.791	0.3794	0.003	0.267	0.021
rh_G_temporal_inf_thickness	0.758	0.6385	0.001	0.140	0.012
rh_G_temporal_middle_thickness	0.009	0.9682	0.000	0.051	-0.001

rh_G.S_cingul.Ant_thickness	0.197	0.8559	0.000	0.073	0.006
rh_G.S_cingul.Mid.Ant_thickness	5.231	0.1555	0.009	0.627	.028*
rh_G.S_cingul.Mid.Post_thickness	5.296	0.1555	0.009	0.632	.025*
rh_G.S_frontomargin_thickness	7.200	0.0769	0.012	0.764	-.040*
rh_G.S_paracentral_thickness	10.832	0.0199	0.017	0.908	.046*
rh_G.S_subcentral_thickness	0.053	0.9087	0.000	0.056	-0.003
rh_G.S_transv_frontopol_thickness	0.206	0.8559	0.000	0.074	-0.007
rh_Lat_Fis.post_thickness	3.903	0.2114	0.006	0.505	.024*
rh_Pole_occipital_thickness	0.173	0.8604	0.000	0.070	0.005
rh_Pole_temporal_thickness	0.717	0.6441	0.001	0.135	0.015
rh_S_calcarine_thickness	3.389	0.2491	0.006	0.452	0.021
rh_S_central_thickness	3.580	0.2349	0.006	0.472	0.019
rh_S_cingul.Marginalis_thickness	4.056	0.2114	0.007	0.520	.023*
rh_S_circular_insula_ant_thickness	0.055	0.9087	0.000	0.056	0.004
rh_S_circular_insula_sup_thickness	1.233	0.5282	0.002	0.198	0.012
rh_S_front_inf_thickness	0.162	0.8629	0.000	0.069	0.004
rh_S_front_middle_thickness	2.653	0.2834	0.004	0.369	0.017
rh_S_front_sup_thickness	13.319	0.0107	0.021	0.954	.038*
rh_S_intrapariet.P_trans_thickness	1.127	0.5501	0.002	0.185	0.010
rh_S_oc_middle.Lunatus_thickness	0.216	0.8559	0.000	0.075	0.006
rh_S_oc_sup.transversal_thickness	0.345	0.807	0.001	0.090	0.007
rh_S_oc.temp_med.Lingual_thickness	2.631	0.2834	0.004	0.367	0.021
rh_S_orbital_med.olfact_thickness	4.105	0.2114	0.007	0.525	.032*
rh_S_orbital.H_Shaped_thickness	0.080	0.9087	0.000	0.059	0.004
rh_S_parieto_occipital_thickness	2.192	0.3146	0.004	0.315	0.017
rh_S_postcentral_thickness	4.440	0.1911	0.007	0.557	.022*
rh_S_precentral.inf.part_thickness	9.066	0.0383	0.015	0.852	.033*
rh_S_suborbital_thickness	0.101	0.9019	0.000	0.062	0.009
rh_S_temporal_inf_thickness	2.509	0.2869	0.004	0.353	-0.021
rh_S_temporal_sup_thickness	0.107	0.9019	0.000	0.062	-0.003
rh_S_temporal_transverse_thickness	5.783	0.1432	0.009	0.670	-.055*

Table S6: individual region outcomes; FND-motor vs controls, cortical thickness, without Euler covariate.

	F	P	ηp^2	Observed power	HC-FND thickness difference (mm³)
lh_G.S_frontomargin_thickness	*0.029*	*0.9058*	*0.000*	*0.053*	*0.003*
lh_G.S_cingul.Ant_thickness	*0.123*	*0.8338*	*0.000*	*0.064*	*0.006*
lh_G_cuneus_thickness	*5.076*	*0.3489*	*0.013*	*0.613*	*.032**
lh_G_front_middle_thickness	*0.447*	*0.7794*	*0.001*	*0.102*	*0.010*
lh_G_occipital_sup_thickness	*0.469*	*0.7794*	*0.001*	*0.105*	*-0.012*
lh_G_oc.temp_med.Lingual_thickness	*1.142*	*0.6371*	*0.003*	*0.187*	*0.014*
lh_G_oc.temp_med.Parahip_thickness	*0.490*	*0.7794*	*0.001*	*0.107*	*-0.019*
lh_G_parietal_sup_thickness	*0.345*	*0.7794*	*0.001*	*0.090*	*-0.009*
lh_G_temp_sup.Lateral_thickness	*0.529*	*0.7794*	*0.001*	*0.112*	*0.016*
lh_G_temp_sup.Plan_polar_thickness	*0.042*	*0.9038*	*0.000*	*0.055*	*0.005*
lh_Pole_occipital_thickness	*2.214*	*0.6054*	*0.006*	*0.317*	*-0.025*
lh_S_calcarine_thickness	*0.814*	*0.735*	*0.002*	*0.147*	*0.012*
lh_S_cingul.Marginalis_thickness	*0.270*	*0.795*	*0.001*	*0.081*	*0.007*
lh_S_collat_transv_post_thickness	*1.196*	*0.6371*	*0.003*	*0.194*	*0.022*
lh_S_front_inf_thickness	*0.111*	*0.8338*	*0.000*	*0.063*	*0.004*
lh_S_front_middle_thickness	*1.125*	*0.6371*	*0.003*	*0.185*	*0.016*
lh_S_front_sup_thickness	*4.648*	*0.3489*	*0.012*	*0.576*	*.028**
lh_S_intrapariet.P_trans_thickness	*0.009*	*0.9229*	*0.000*	*0.051*	*-0.001*
lh_S_oc_middle.Lunatus_thickness	*0.184*	*0.795*	*0.000*	*0.071*	*0.007*
lh_S_oc_sup.transversal_thickness	*0.208*	*0.795*	*0.001*	*0.074*	*0.007*
lh_S_orbital_lateral_thickness	*0.387*	*0.7794*	*0.001*	*0.095*	*-0.015*
lh_S_orbital.H_Shaped_thickness	*0.208*	*0.795*	*0.001*	*0.074*	*-0.008*
lh_S_postcentral_thickness	*0.019*	*0.9109*	*0.000*	*0.052*	*0.002*
lh_S_suborbital_thickness	*2.481*	*0.6054*	*0.006*	*0.349*	*0.041*
rh_G.S_frontomargin_thickness	*2.060*	*0.608*	*0.005*	*0.299*	*-0.027*
rh_G.S_transv_frontopol_thickness	*1.506*	*0.6371*	*0.004*	*0.232*	*0.024*
rh_G.S_cingul.Ant_thickness	*2.793*	*0.6003*	*0.007*	*0.385*	*-0.027*
rh_G_cuneus_thickness	*8.646*	*0.0765*	*0.022*	*0.835*	*.040**
rh_G_occipital_sup_thickness	*0.369*	*0.7794*	*0.001*	*0.093*	*-0.011*
rh_G_oc.temp_med.Lingual_thickness	*1.425*	*0.6371*	*0.004*	*0.222*	*0.017*
rh_G_parietal_sup_thickness	*3.355*	*0.4971*	*0.009*	*0.447*	*-0.028*
rh_G_temp_sup.G_T_transv_thickness	*0.188*	*0.795*	*0.000*	*0.072*	*0.010*
rh_G_temp_sup.Lateral_thickness	*2.316*	*0.6054*	*0.006*	*0.330*	*0.030*
rh_Pole_occipital_thickness	*0.819*	*0.735*	*0.002*	*0.147*	*-0.013*
rh_S_cingul.Marginalis_thickness	*1.276*	*0.6371*	*0.003*	*0.203*	*-0.017*
rh_S_front_middle_thickness	*0.684*	*0.7708*	*0.002*	*0.131*	*-0.012*
rh_S_front_sup_thickness	*1.292*	*0.6371*	*0.003*	*0.205*	*0.016*
rh_S_intrapariet.P_trans_thickness	*1.290*	*0.6371*	*0.003*	*0.205*	*-0.014*
rh_S_oc_sup.transversal_thickness	*0.040*	*0.9038*	*0.000*	*0.055*	*0.003*
rh_S_orbital_lateral_thickness	*11.836*	*0.0284*	*0.030*	*0.929*	*-.067**
rh_S_orbital_med.olfact_thickness	*0.329*	*0.7794*	*0.001*	*0.088*	*-0.011*
rh_S_parieto_occipital_thickness	*0.651*	*0.7708*	*0.002*	*0.127*	*0.011*
rh_S_postcentral_thickness	*1.171*	*0.6371*	*0.003*	*0.190*	*-0.014*
rh_S_precentral.sup.part_thickness	*4.058*	*0.3932*	*0.011*	*0.520*	*.035**

Table S7: individual region outcomes; FS vs controls, cortical thickness.

Matched subgroups

Depression

	**Depression$^+$**	**Depression$^-$**	
n	118	118	
Female	93 (78.8%)	93 (78.8%)	P=1.0
Age	37.9 (12.4)	37.9 (12.4)	P=0.92
FND-motor (%)	72 (61.0%)	68 (57.6%)	P=0.60

Table S8: Breakdown of cases used in the depression subgroup analyses.

There were no differences between FND cases with a lifetime history of depression (depression+) vs. those without a lifetime history of depression (depression-) in sex, age, and proportion of FND-motor cases.

Anxiety

	**Anxiety$^+$**	**Anxiety$^-$**	
n	105	105	
Female	80 (76.19%)	80 (76.19%)	P=1.0
Age	37.42 (12.43)	37.42 (12.44)	P=0.963
FND-motor (%)	80 (76.19%)	52 (49.52%)	P<0.001

Table S9: Breakdown of cases used in the anxiety subgroup.

There were no differences between FND cases with a lifetime history of anxiety (anxiety$^+$) vs. those without a history of anxiety (anxiety$^-$) in sex, age, and proportion of FND-motor cases. We didn't match on FND subtype too as would make the already small groups even smaller.

	**FND-motor**	**Controls**	
n	307	307	
Female	224 (73.0%)	224 (73.0%)	P=1.0
Age	40.1	40.1	P=0.944
	**FND-seiz**	**Controls**	
n	192	192	
Female	152 (79.2%)	152 (79.2%)	P=1.0
Age	34.7 (11.8)	34.9 (11.7)	P=0.734

Table S10: Breakdown of cases used in the subgroup vs. HC comparisons.

There were no differences between FND-motor and FND-seizures versus controls in sex and age.

FND-motor vs FND-seiz

	**Motor**	**Seizures**		**p**
Female	138	138	Chi square = 0.000	1.000
Male	36	36		
Age	35.4	35.4	F=0.005	0.993

Table S11: breakdown of cases in FND-motor vs FND-seiz comparisons.

There were no differences between FND-motor versus FND-seizures in the age and sex variables.

FND sex matched

	**Females**	**Males**	Chi square	**p**
n	119	119	0.000	1.000
Age	39.5 (13.0)	39.5 (13.0)		
FND-Motor (%)	82 (68.9)	80 (67.2)	0.77	0.781

Table S12: breakdown of cases in the sex-matched FND group.

There were no differences between females (n=119) vs. males (n=119) in age and proportion of FND-motor.

Feature selection principal component analysis (PCA)

For surface area, we used relative surface area as described in the main text.

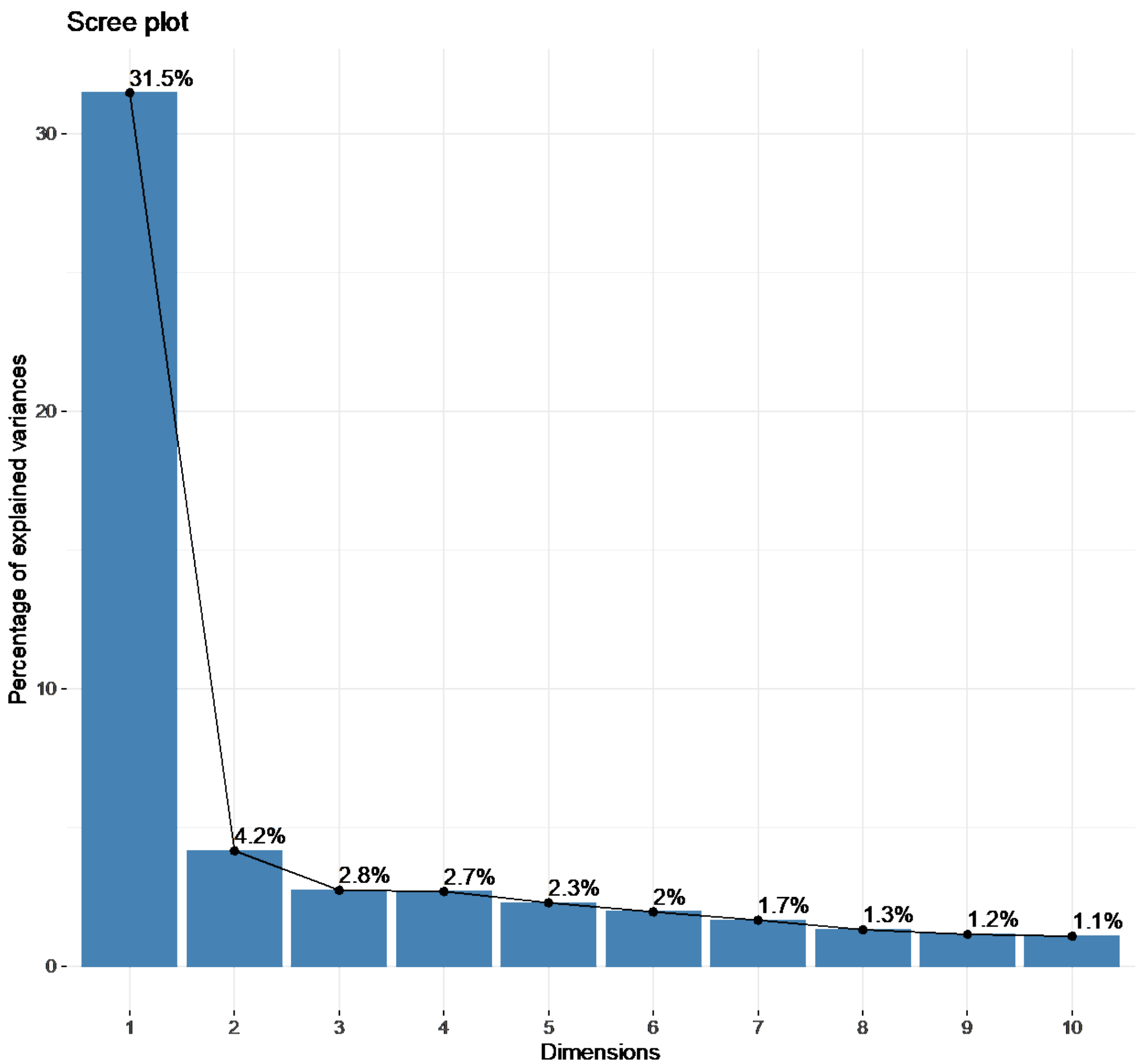

Figure S1: Full sample thickness PCA scree plot

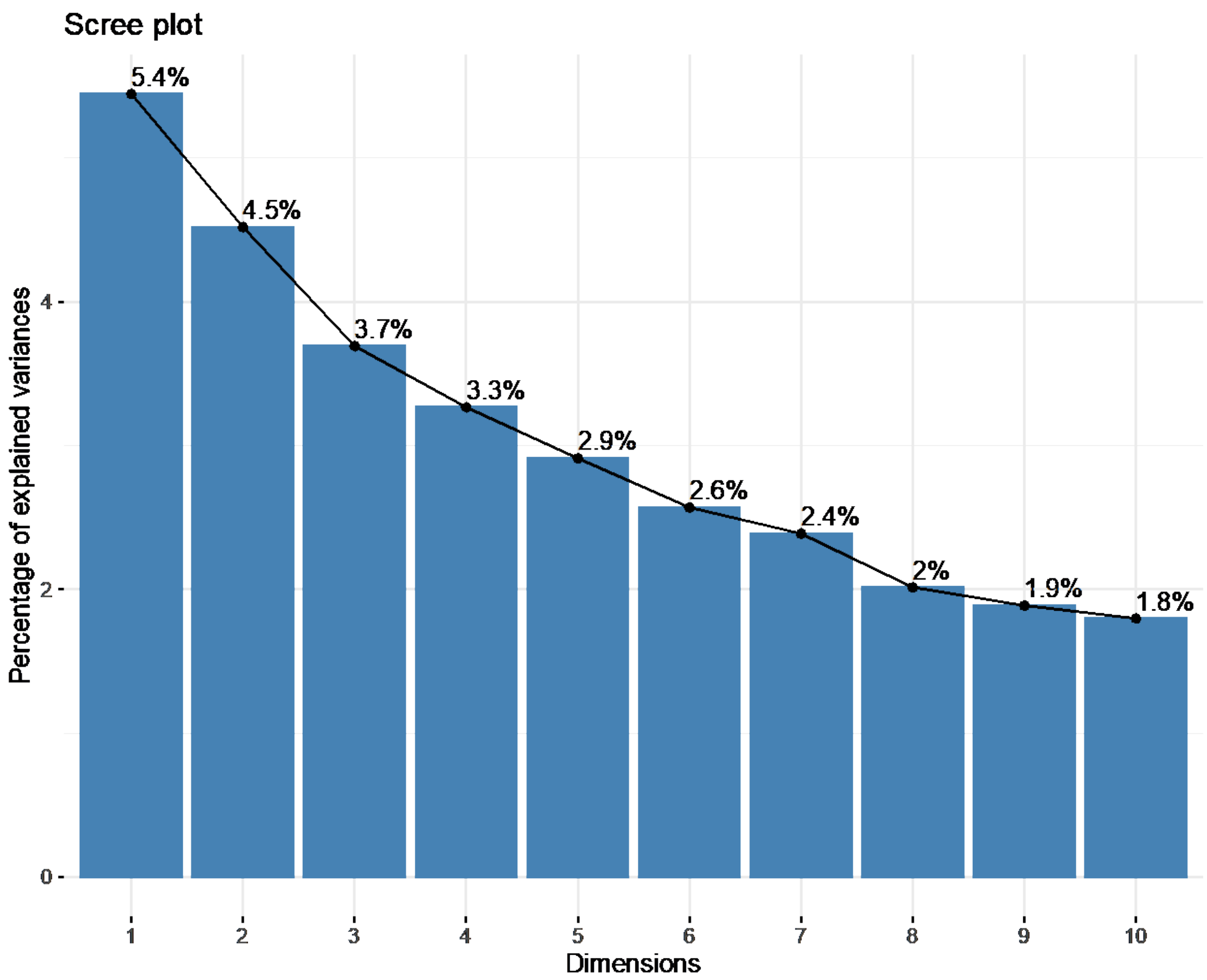

Figure S2: Full sample area PCA scree plot

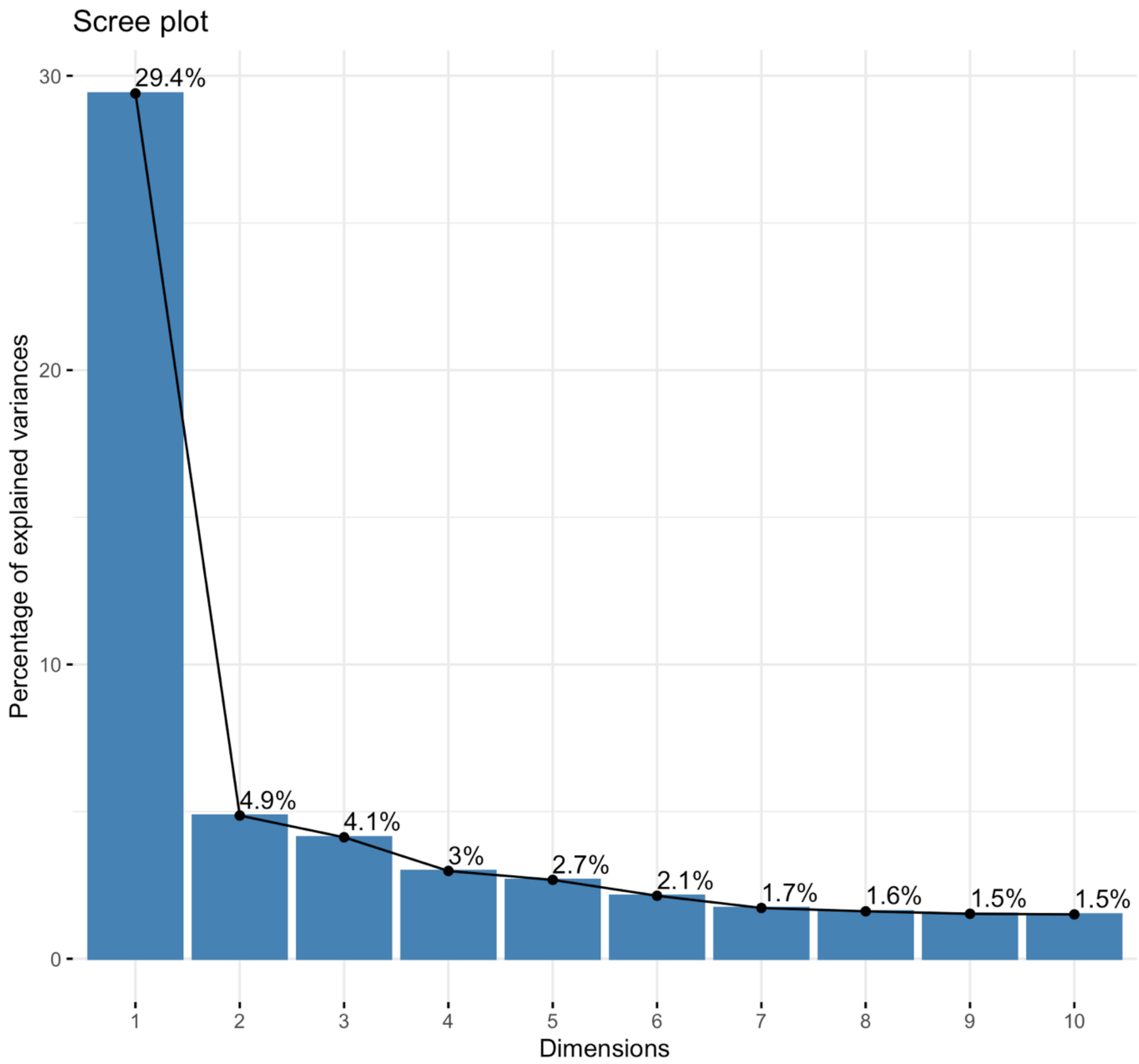

Figure S3: Anxiety matched thickness PCA scree plot

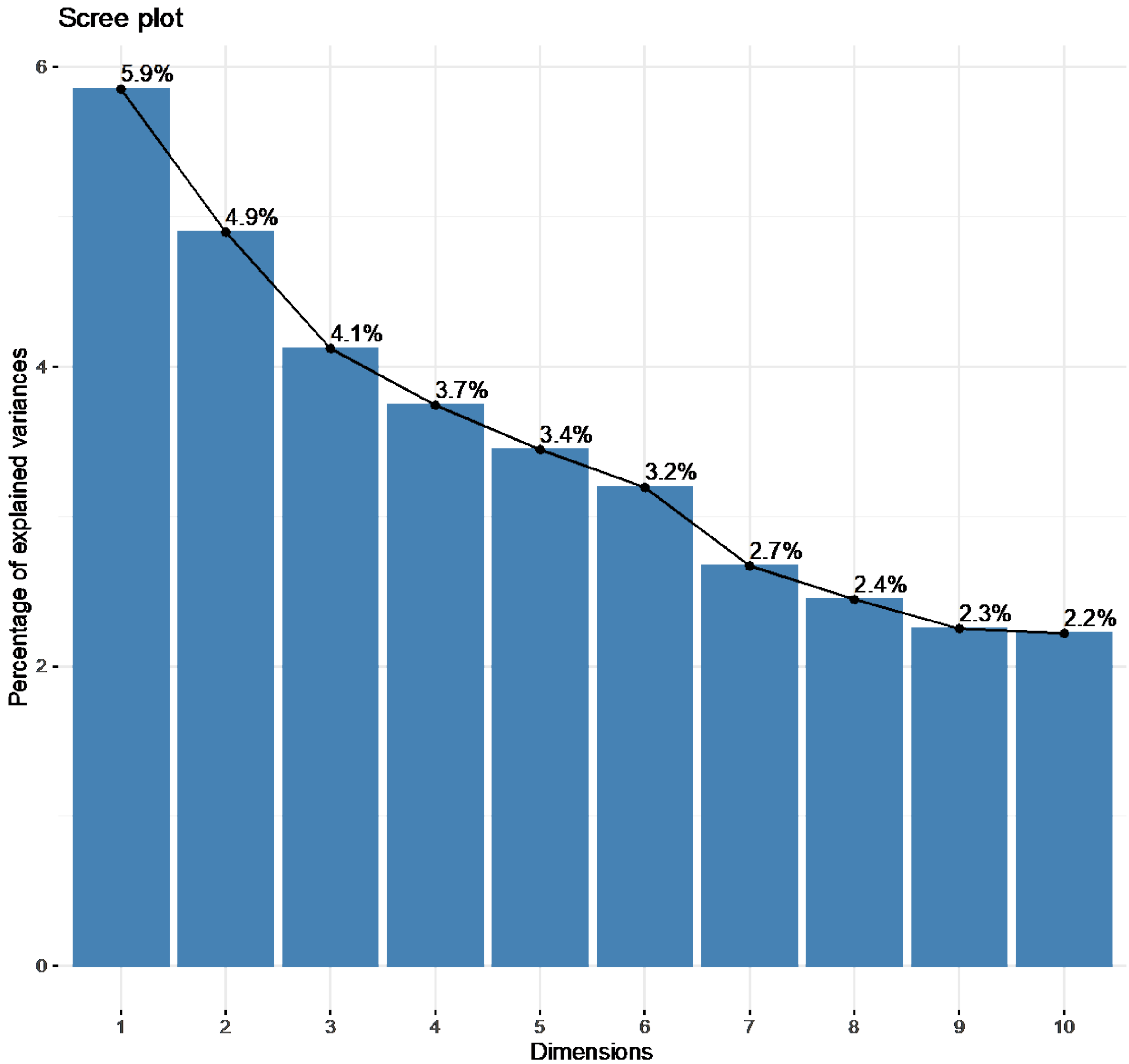

Figure S4: Anxiety matched area PCA scree plot.

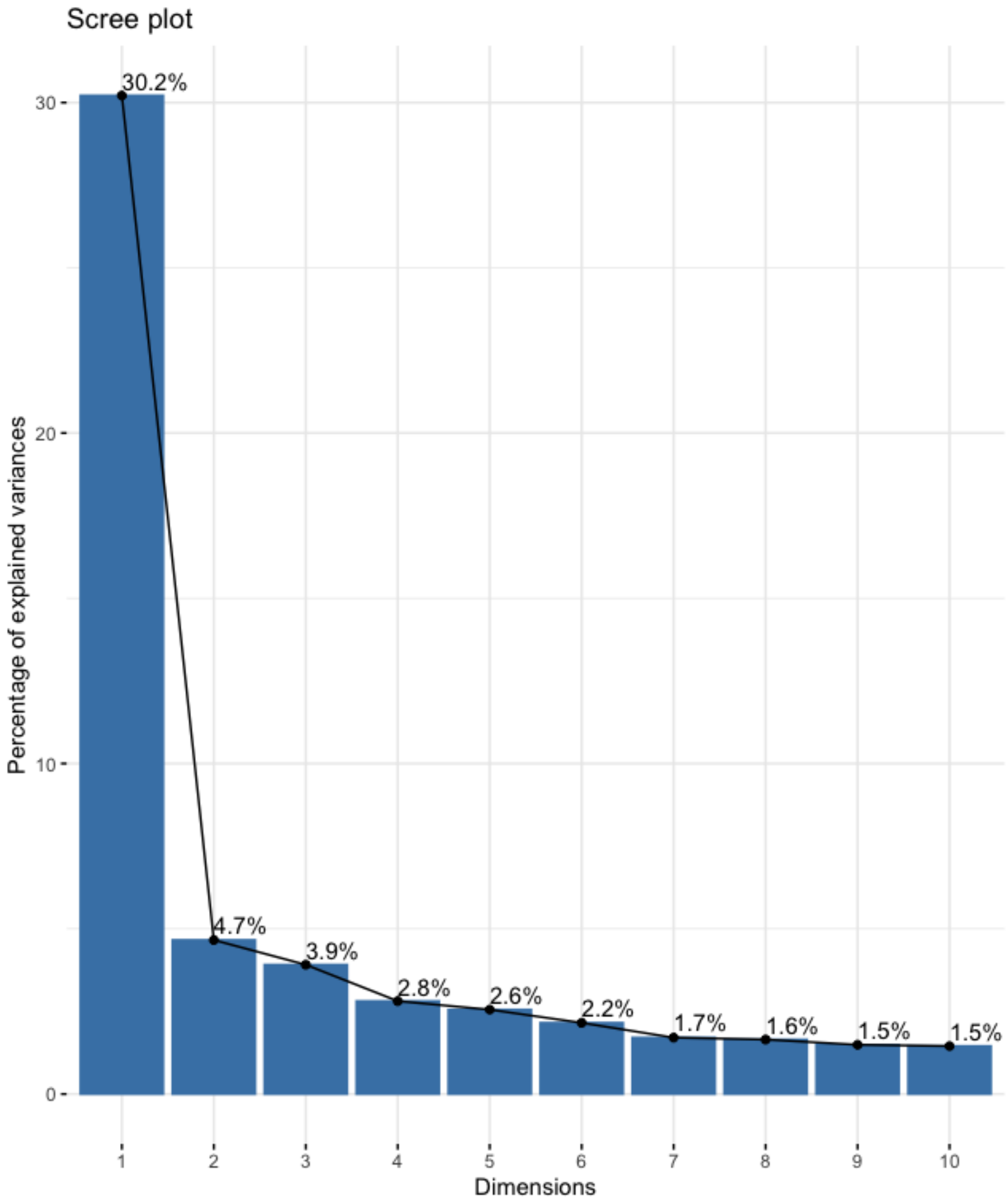

Figure S5: Depression matched thickness PCA scree plot.

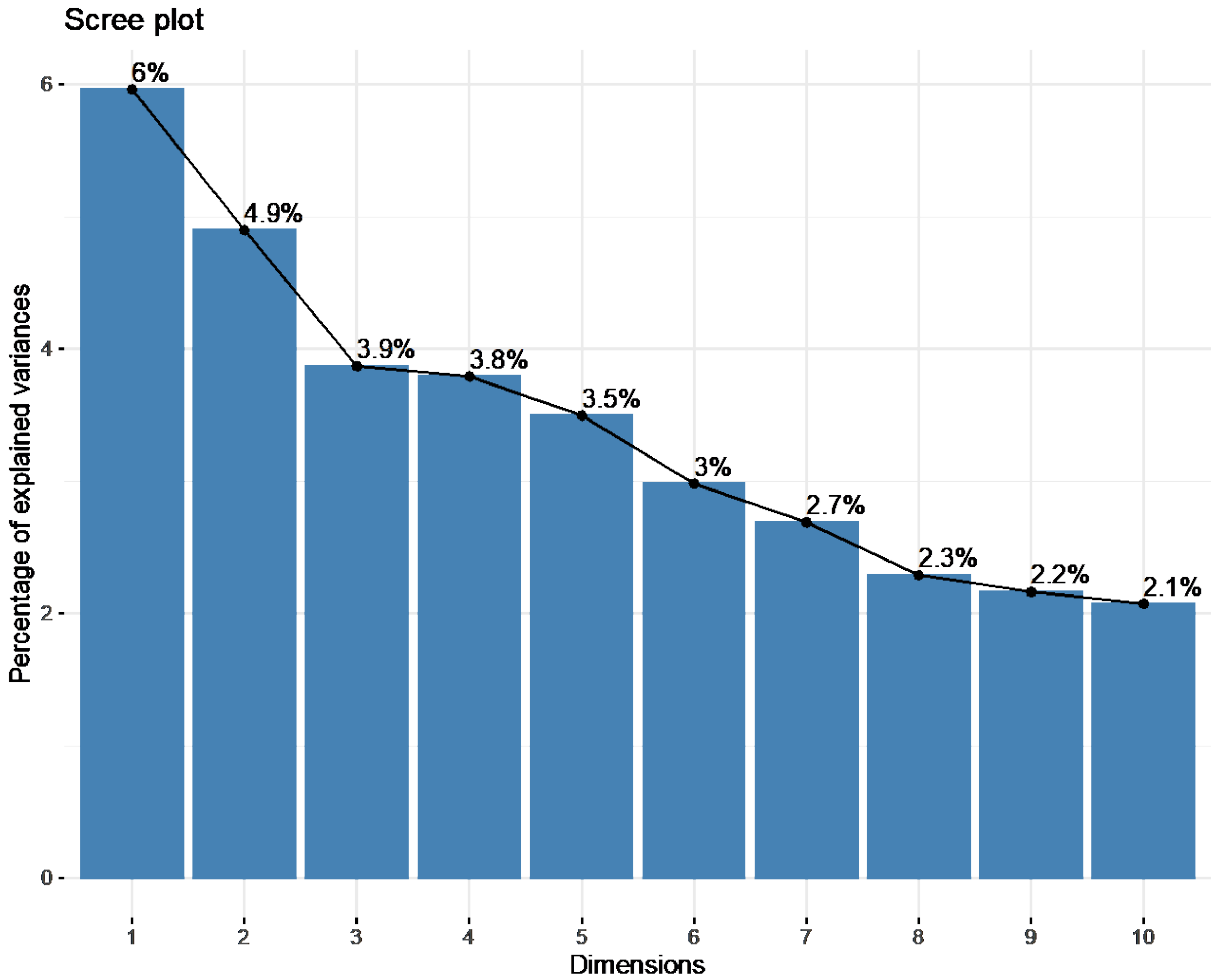

Figure S6: Depression matched area PCA scree plot.

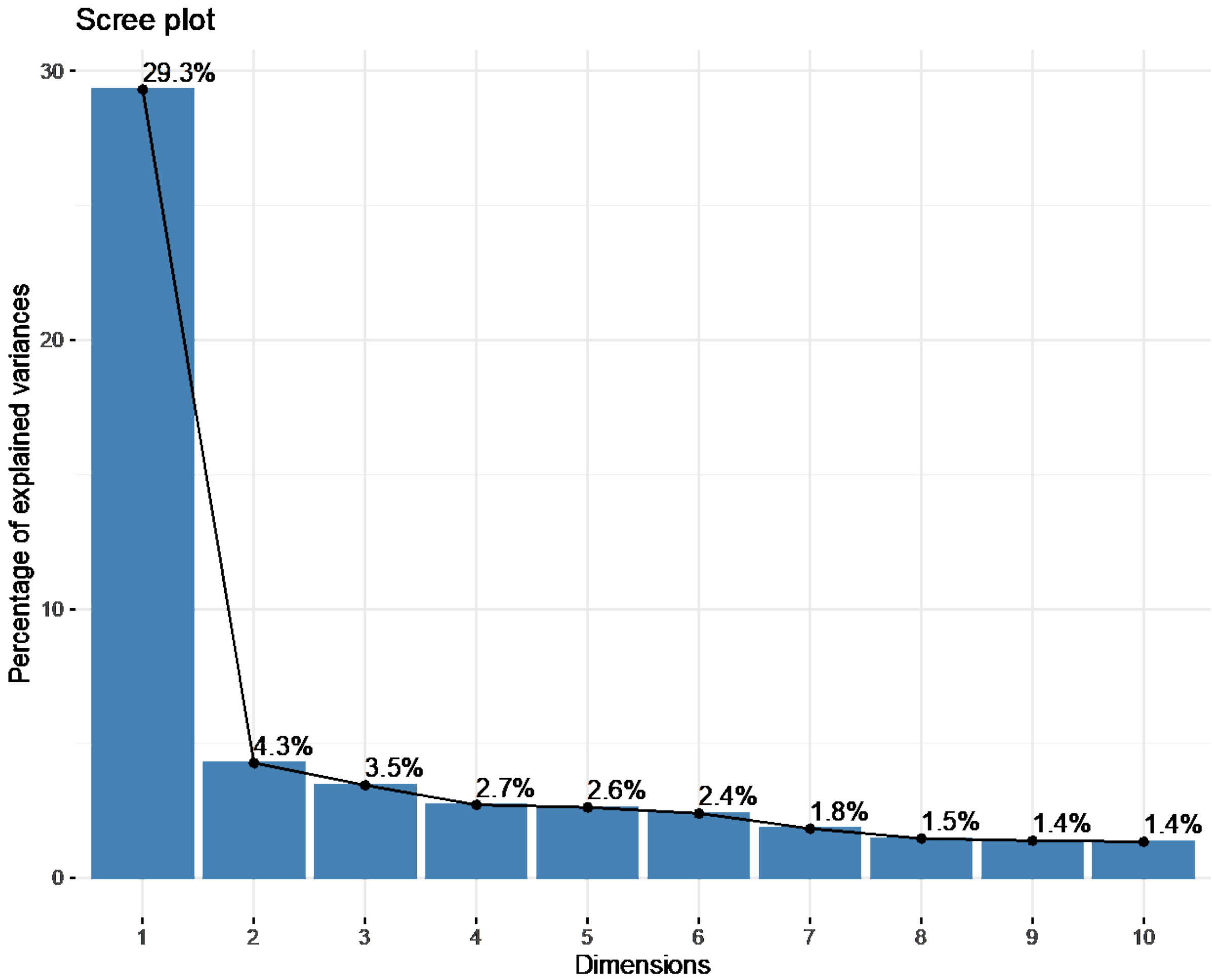

Figure S7: FMD – FS matched thickness scree plot.

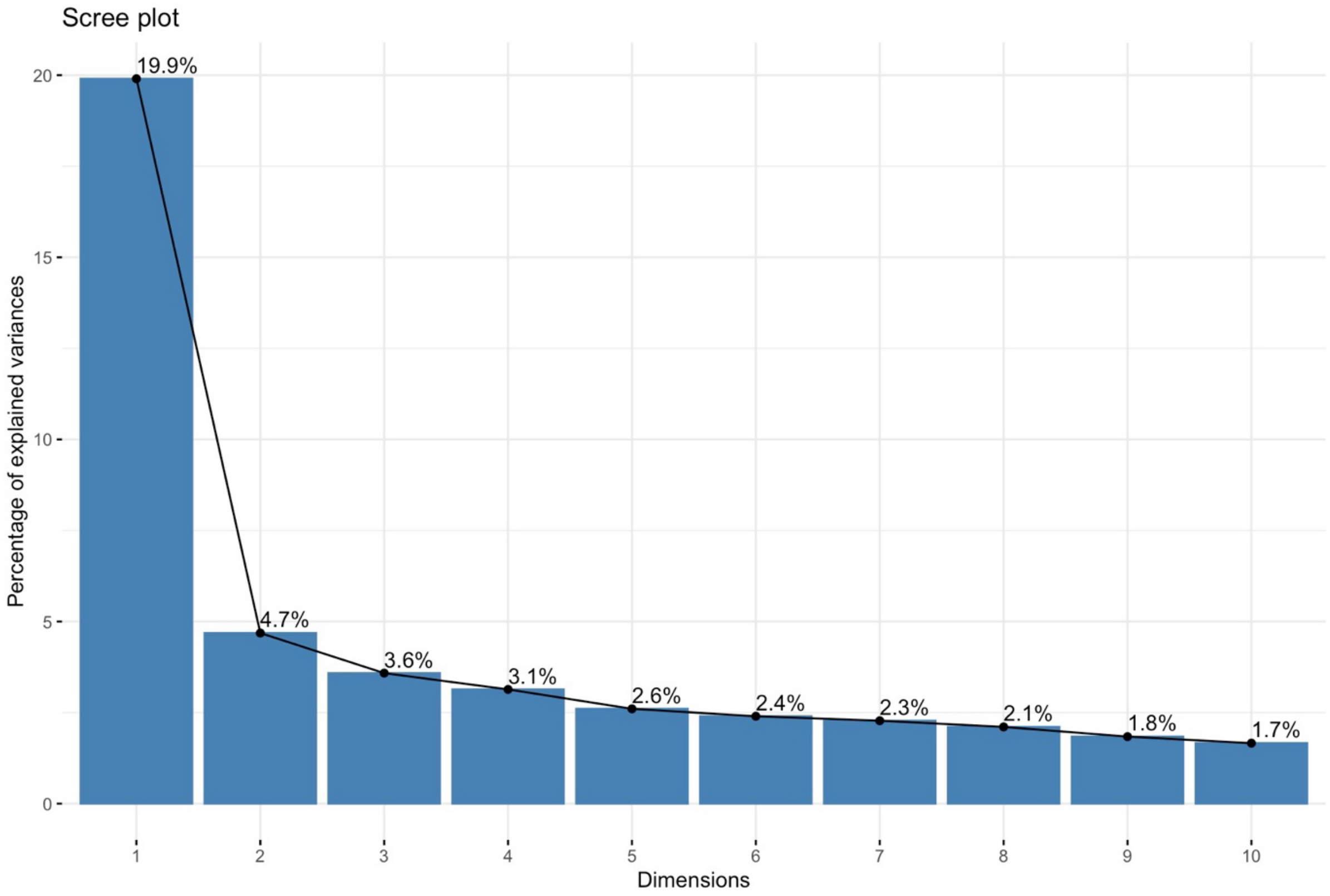

Figure S8: FMD – FS matched area scree plot.

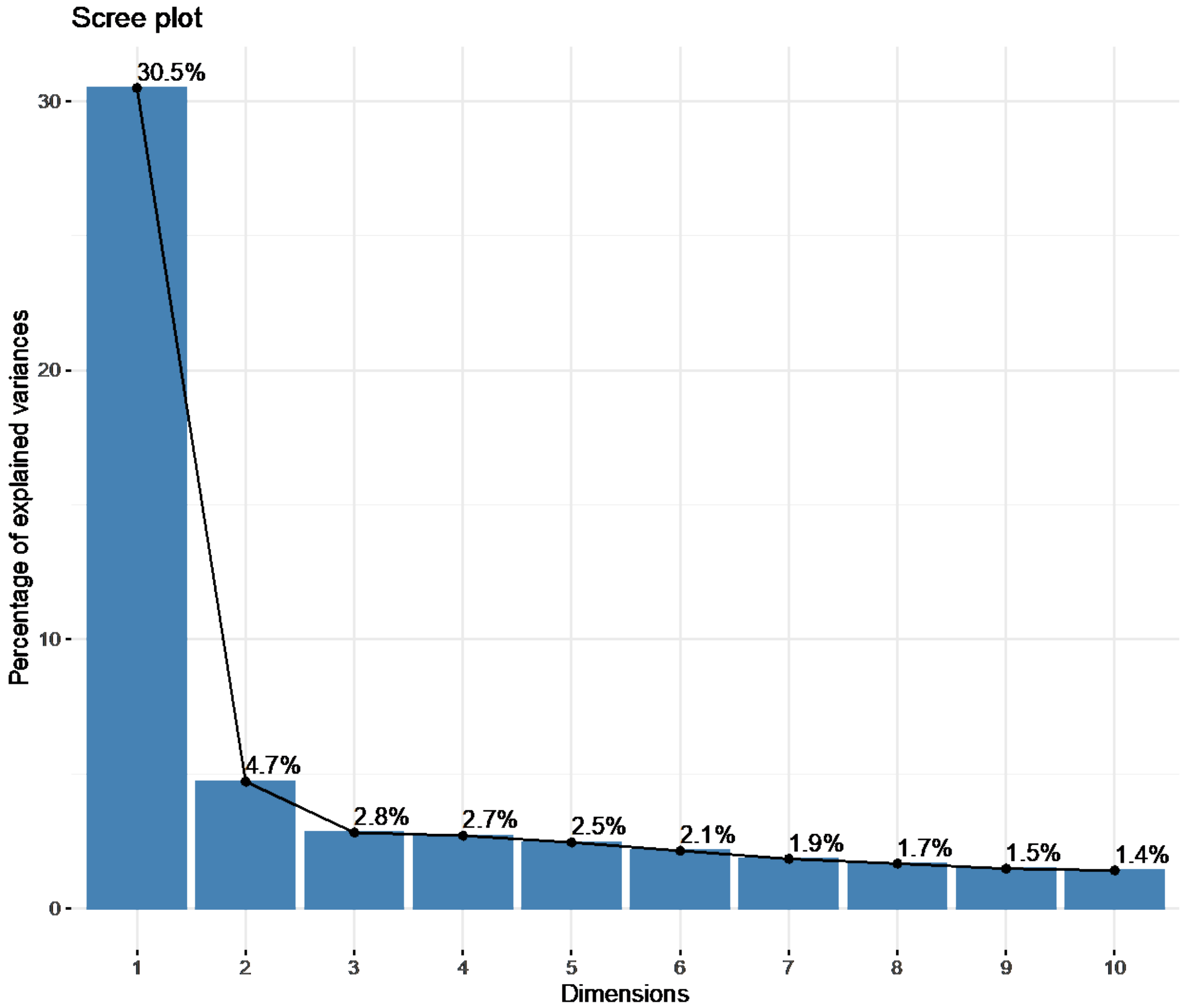

Figure S9: Sex matched PCA thickness scree plot.

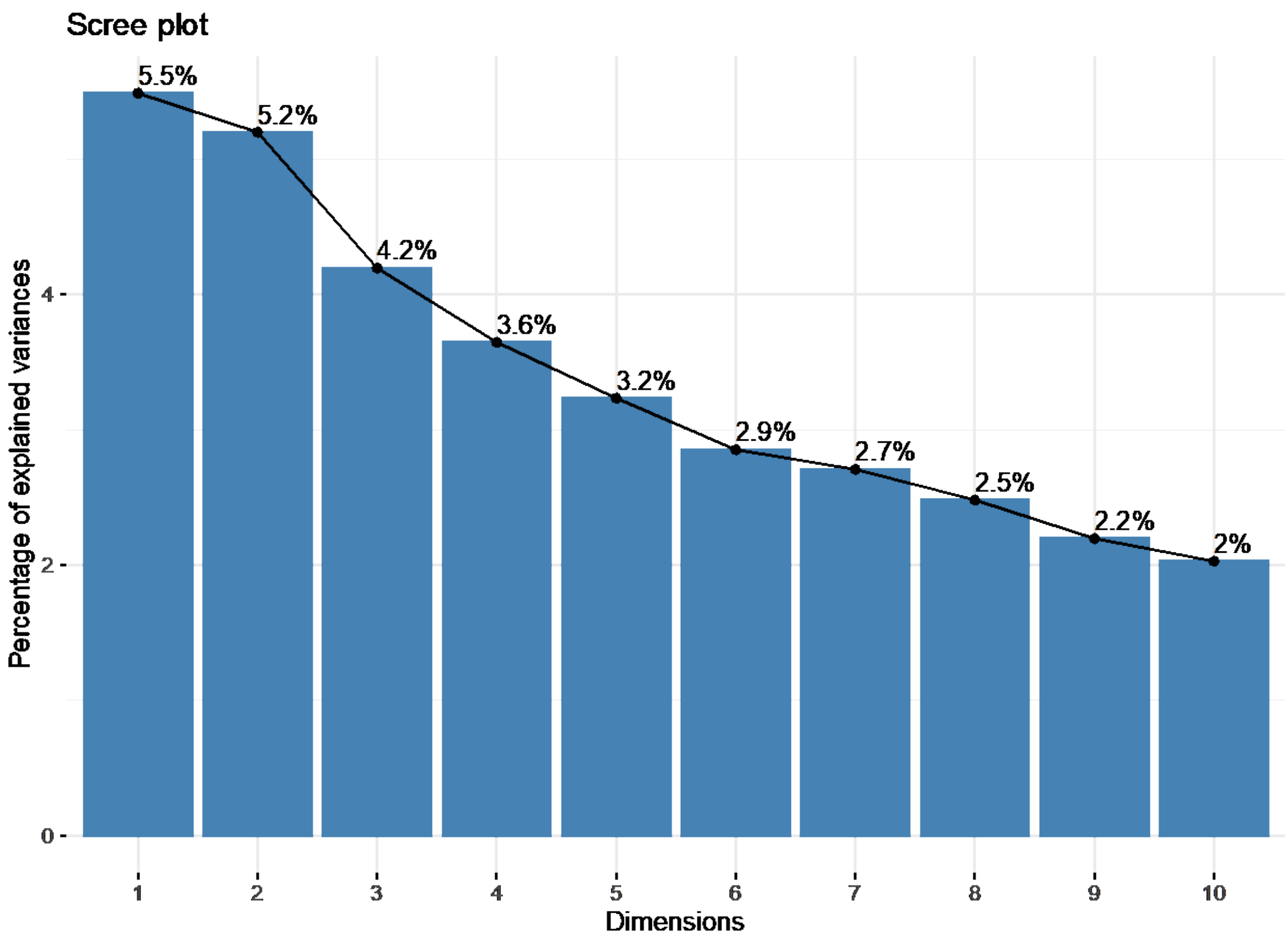

Figure S10: Sex matched PCA area scree plot.

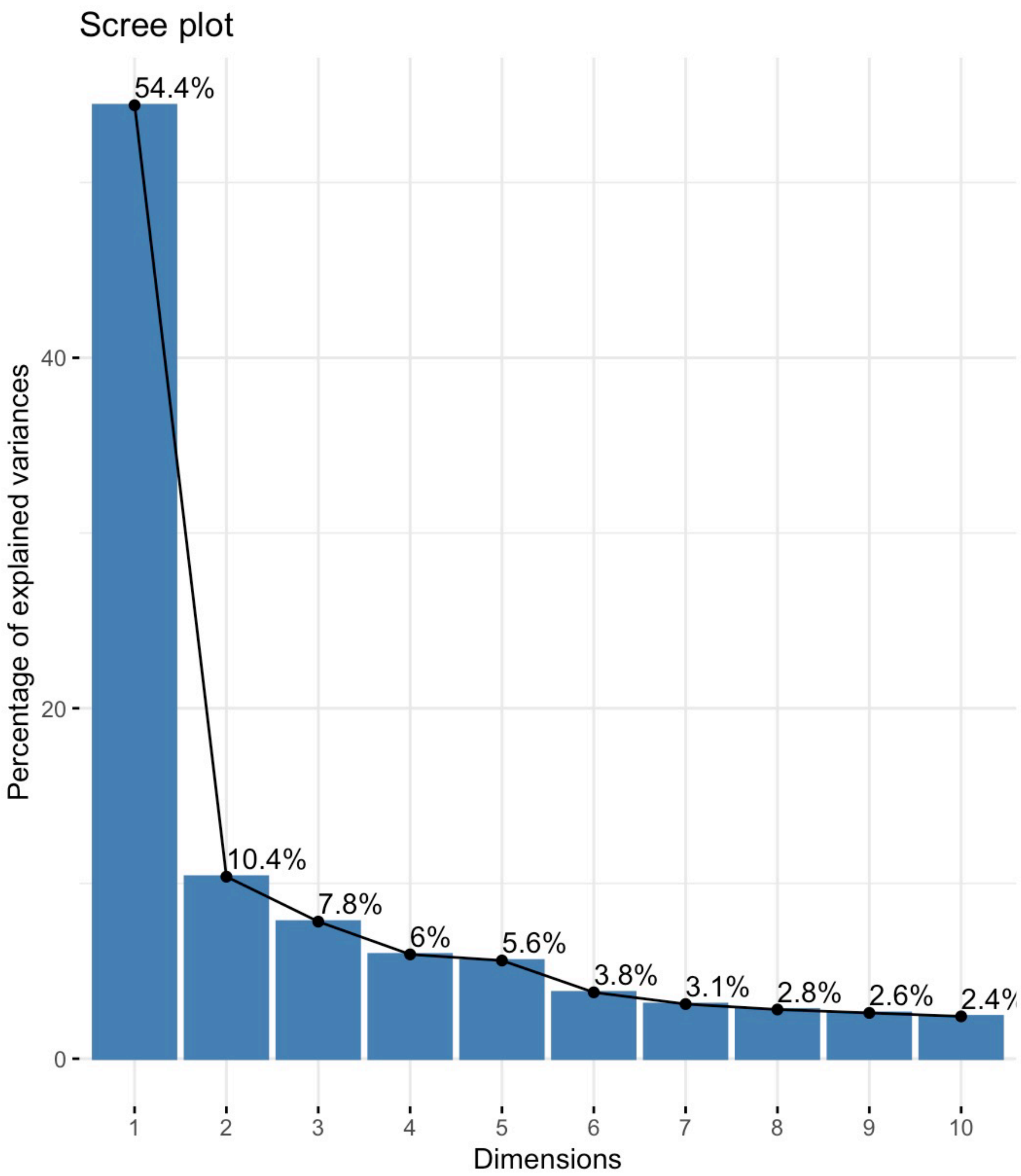

Figure S11: Exploratory PCA – cortical thickness scree plot.

<u>Leave one out analyses</u>

	Site 1		Site 2		Site 3		Site 4		Site 5		Site 6		Site 7		Site 8		Site 9		Site 10		Site 11		Site 12		Site 13		Site 14		Site 15	
	**HC - FND**	**P**	**HC - FND**	**P**	**HC - FND**	**P**	**HC - FND**	**P**	**HC - FND**	**P**	**HC - FND**	**P**	**HC - FND**	**P**	**HC - FND**	**P**	**HC - FND**	**P**	**HC - FND**	**P**	**HC - FND**	**P**	**HC - FND**	**P**	**HC - FND**	**P**	**HC - FND**	**P**	**HC - FND**	**P**
lh_G_cingul.Post.dorsal_thickness	0.006	0.579	0.002	0.890	0.004	0.726	0.007	0.540	0.007	0.516	0.006	0.624	0.008	0.490	0.008	0.464	0.006	0.594	0.006	0.604	0.006	0.617	0.011	0.379	0.003	0.754	0.007	0.512	0.004	0.746
lh_G_cuneus_thickness	0.016	0.060	0.016	0.075	0.016	0.083	0.013	0.136	0.016	0.071	0.012	0.177	0.016	0.061	0.016	0.062	.019*	**0.044**	.020*	**0.023**	0.015	0.088	.020*	**0.030**	0.015	0.080	0.015	0.090	0.013	0.148
lh_G_front_inf.Opercular_thickness	0.019	0.072	.023*	**0.032**	0.020	0.064	.023*	**0.033**	0.020	0.057	0.010	0.372	.022*	**0.040**	.024*	**0.027**	.024*	**0.033**	.023*	**0.036**	0.018	0.095	.023*	**0.047**	0.019	0.072	0.021	0.051	0.020	0.067
lh_G_front_inf.Orbital_thickness	0.008	0.584	0.005	0.726	0.010	0.497	0.010	0.488	0.008	0.579	0.002	0.871	0.010	0.492	0.009	0.551	0.009	0.558	0.014	0.340	0.006	0.682	0.006	0.713	0.006	0.693	0.010	0.491	0.007	0.656
lh_G_front_inf.Triangul_thickness	0.009	0.456	0.010	0.389	0.001	0.963	0.009	0.446	0.007	0.526	0.00	0.994	0.010	0.359	0.011	0.323	0.013	0.298	0.009	0.436	0.008	0.469	0.007	0.585	0.007	0.539	0.008	0.480	0.005	0.631
lh_G_front_middle_thickness	0.017	0.077	0.015	0.120	0.012	0.203	0.017	0.069	0.017	0.080	0.008	0.424	0.017	0.071	.022*	**0.021**	0.019	0.064	0.015	0.126	0.014	0.137	0.018	0.078	0.017	0.071	0.018	0.064	0.015	0.106
lh_G_front_sup_thickness	.034*	**0.001**	.034*	**0.001**	.035*	**0.001**	.033*	**0.001**	.034*	**0.001**	.027*	**0.010**	.036*	**0.000**	.039*	**0.000**	.034*	**0.001**	.035*	**0.001**	.033*	**0.001**	.034*	**0.002**	.034*	**0.001**	.033*	**0.001**	.033*	**0.001**
lh_G_Ins_lg.S_cent_ins_thickness	0.006	0.732	0.001	0.973	0.000	0.992	0.012	0.486	0.006	0.735	-0.007	0.688	0.006	0.737	0.006	0.724	-0.011	0.552	0.008	0.665	0.003	0.839	0.020	0.289	-0.001	0.956	0.008	0.635	0.000	0.986
lh_G_oc.temp_med.Parahip_thickness	-0.005	0.773	-0.012	0.490	-0.016	0.366	-0.015	0.392	-0.004	0.795	-0.010	0.596	-0.007	0.672	-0.005	0.776	-0.016	0.366	-0.002	0.910	-0.015	0.387	0.006	0.763	-0.007	0.687	-0.008	0.652	-0.012	0.466
lh_G_occipital_middle_thickness	0.014	0.151	0.011	0.255	0.011	0.260	0.014	0.139	0.010	0.299	0.003	0.733	0.012	0.197	0.014	0.155	0.013	0.212	0.007	0.490	0.013	0.197	0.016	0.113	0.012	0.202	0.011	0.252	0.013	0.177
lh_G_occipital_sup_thickness	0.000	0.982	-0.002	0.889	-0.001	0.957	0.000	0.990	-0.003	0.832	-0.002	0.843	-0.002	0.883	-0.001	0.901	0.002	0.898	-0.003	0.821	-0.004	0.765	0.001	0.954	-0.002	0.881	-0.001	0.904	0.002	0.898
lh_G_orbital_thickness	0.008	0.422	0.007	0.496	-0.001	0.907	0.008	0.477	0.007	0.514	0.001	0.938	0.006	0.557	0.009	0.373	0.003	0.808	0.007	0.534	0.003	0.766	0.009	0.412	0.007	0.505	0.006	0.582	0.006	0.559
lh_G_pariet_inf.Angular_thickness	0.003	0.739	0.000	0.992	-0.004	0.699	0.003	0.725	0.003	0.750	-0.004	0.707	0.002	0.859	0.007	0.474	0.005	0.625	-0.003	0.748	0.001	0.957	0.002	0.847	0.001	0.880	-0.001	0.890	0.000	0.979
lh_G_pariet_inf.Supramar_thickness	0.002	0.864	-0.001	0.895	-0.002	0.834	0.002	0.877	-0.001	0.930	-0.008	0.434	0.001	0.938	0.003	0.751	0.008	0.467	-0.004	0.731	0.000	0.970	0.000	0.989	0.000	0.976	0.000	0.984	-0.002	0.851
lh_G_parietal_sup_thickness	-0.004	0.666	-0.007	0.429	-0.005	0.620	-0.005	0.592	-0.006	0.536	-0.016	0.100	-0.003	0.742	0.000	0.968	-0.006	0.574	-0.006	0.499	-0.004	0.651	0.000	0.987	-0.004	0.646	-0.006	0.494	-0.006	0.502
lh_G_postcentral_thickness	0.019	0.064	0.020	0.059	0.016	0.126	0.016	0.124	0.017	0.094	0.011	0.329	0.016	0.109	0.017	0.105	0.019	0.086	0.016	0.125	0.013	0.191	0.018	0.109	0.015	0.148	0.014	0.164	0.018	0.084
lh_G_precentral_thickness	.035*	**0.005**	.031*	**0.016**	.033*	**0.011**	.034*	**0.008**	.035*	**0.006**	.028*	**0.034**	.033*	**0.008**	.027*	**0.034**	.042*	**0.002**	.032*	**0.014**	.032*	**0.012**	0.025	0.067	.032*	**0.012**	.031*	**0.015**	.029*	**0.023**
lh_G_precuneus_thickness	0.015	0.235	0.016	0.228	0.020	0.141	0.009	0.449	0.014	0.273	0.006	0.645	0.019	0.143	0.020	0.129	0.008	0.573	0.012	0.346	0.015	0.231	0.019	0.174	0.015	0.244	0.016	0.216	0.014	0.272
lh_G_rectus_thickness	0.001	0.926	######	0.995	0.000	0.978	-0.004	0.764	0.004	0.759	-0.001	0.925	-0.001	0.948	0.007	0.536	-0.014	0.251	0.001	0.943	-0.001	0.911	0.001	0.930	0.000	0.990	0.001	0.948	-0.003	0.829
lh_G_temp_sup.G_T_transv_thickness	0.011	0.470	0.008	0.609	0.007	0.669	0.008	0.573	0.006	0.666	0.011	0.480	0.006	0.705	0.008	0.586	0.014	0.373	0.014	0.360	0.004	0.784	-0.010	0.538	0.008	0.602	0.013	0.383	0.002	0.869

lh_G_temp_sup.Lateral_thickness	0.019	0.176	0.018	0.205	0.022	0.132	0.016	0.239	0.018	0.199	0.010	0.489	0.020	0.144	0.026	0.063	.035*	**0.017**	0.016	0.268	0.014	0.292	0.011	0.450	0.016	0.229	0.017	0.212	0.013	0.344
lh_G_temporal_middle_thickness	0.011	0.374	0.007	0.555	0.003	0.800	0.007	0.586	0.011	0.362	0.001	0.909	0.008	0.510	0.015	0.228	0.020	0.125	0.001	0.933	0.007	0.543	0.003	0.813	0.009	0.456	0.008	0.539	0.008	0.524
lh_G.S_cingul.Ant_thickness	0.000	0.984	0.003	0.775	-0.001	0.924	0.002	0.865	0.002	0.824	-0.006	0.603	0.005	0.622	0.010	0.342	0.001	0.917	0.000	0.969	-0.001	0.882	0.011	0.298	0.004	0.679	0.001	0.953	0.000	0.963
lh_G.S_cingul.Mid.Ant_thickness	0.014	0.197	0.014	0.199	0.011	0.333	0.014	0.220	0.013	0.246	0.011	0.364	0.017	0.118	.023*	**0.035**	0.010	0.394	0.013	0.247	0.011	0.312	0.015	0.212	0.016	0.132	0.016	0.154	0.015	0.175
lh_G.S_cingul.Mid.Post_thickness	0.011	0.219	0.011	0.260	0.013	0.164	0.011	0.248	0.009	0.344	0.007	0.454	0.014	0.124	0.014	0.123	0.012	0.215	0.010	0.315	0.011	0.222	0.012	0.211	0.012	0.193	0.009	0.320	0.013	0.178
lh_G.S_frontomargin_thickness	0.010	0.372	0.008	0.470	0.005	0.661	0.011	0.331	0.009	0.442	0.002	0.849	0.008	0.489	0.014	0.195	0.011	0.357	0.009	0.434	0.008	0.484	0.015	0.219	0.009	0.421	0.009	0.443	0.007	0.515
lh_G.S_paracentral_thickness	.034*	**0.003**	.036*	**0.002**	.030*	**0.013**	.035*	**0.003**	.033*	**0.005**	.027*	**0.027**	.033*	**0.005**	.034*	**0.004**	.037*	**0.003**	.034*	**0.005**	.030*	**0.010**	.029*	**0.025**	.031*	**0.008**	.029*	**0.014**	.028*	**0.017**
lh_G.S_subcentral_thickness	0.003	0.791	0.002	0.863	-0.001	0.946	-0.001	0.958	-0.003	0.821	-0.005	0.694	0.002	0.851	0.002	0.857	0.015	0.210	0.001	0.964	-0.001	0.900	-0.002	0.863	0.000	0.975	0.000	0.985	-0.001	0.930
lh_Lat_Fis.post_thickness	0.019	0.065	.021*	**0.044**	0.019	0.080	.021*	**0.044**	0.016	0.119	0.015	0.169	0.019	0.057	0.018	0.086	.023*	**0.039**	0.017	0.102	0.019	0.068	.027*	**0.016**	0.017	0.098	0.017	0.108	0.016	0.130
lh_Pole_occipital_thickness	-0.017	0.101	-0.016	0.123	-0.019	0.080	-0.015	0.152	-0.018	0.082	-0.020	0.067	-0.017	0.097	-0.019	0.067	-.023*	**0.037**	-0.020	0.056	-0.018	0.092	-0.003	0.815	-0.016	0.115	-0.016	0.126	-0.020	0.061
lh_Pole_temporal_thickness	0.017	0.250	0.019	0.202	0.011	0.466	0.015	0.307	0.014	0.332	0.007	0.640	0.018	0.221	0.025	0.085	0.013	0.393	0.027	0.076	0.015	0.317	0.021	0.201	0.012	0.410	0.015	0.314	0.013	0.365
lh_S_central_thickness	.019*	**0.018**	.021*	**0.012**	.020*	**0.018**	.017*	**0.035**	.019*	**0.018**	.017*	**0.047**	.019*	**0.018**	.021*	**0.009**	.020*	**0.021**	.020*	**0.017**	.017*	**0.036**	.022*	**0.012**	.019*	**0.022**	.017*	**0.037**	0.016	0.050
lh_S_circular_insula_ant_thickness	0.011	0.415	0.010	0.468	0.007	0.632	0.013	0.344	0.016	0.272	0.002	0.916	0.012	0.395	0.015	0.282	0.004	0.776	0.019	0.196	0.012	0.404	0.020	0.181	0.009	0.520	0.009	0.547	0.010	0.461
lh_S_circular_insula_inf_thickness	0.010	0.430	0.007	0.582	0.014	0.272	0.007	0.542	0.010	0.419	0.010	0.422	0.011	0.384	0.015	0.237	0.003	0.831	0.008	0.507	0.007	0.594	0.013	0.306	0.006	0.636	0.008	0.504	0.005	0.707
lh_S_circular_insula_sup_thickness	0.001	0.896	0.005	0.598	0.002	0.851	0.004	0.662	0.001	0.912	-0.003	0.739	0.004	0.673	0.005	0.555	0.000	0.986	0.006	0.525	-0.002	0.828	0.005	0.615	0.000	0.979	-0.003	0.761	0.001	0.947
lh_S_front_inf_thickness	0.010	0.255	0.011	0.224	0.006	0.489	0.011	0.218	0.011	0.217	0.003	0.719	0.010	0.224	.017*	**0.045**	0.014	0.139	0.010	0.241	0.008	0.377	0.014	0.142	0.010	0.244	0.008	0.347	0.008	0.350
lh_S_front_middle_thickness	0.013	0.167	0.013	0.192	0.015	0.148	0.015	0.123	0.013	0.190	0.006	0.595	0.015	0.116	.020*	**0.038**	0.016	0.130	0.015	0.139	0.012	0.238	0.020	0.056	0.016	0.105	0.014	0.146	0.012	0.240
lh_S_front_sup_thickness	.026*	**0.002**	.028*	**0.001**	.029*	**0.001**	.027*	**0.002**	.028*	**0.001**	.021*	**0.018**	.028*	**0.001**	.034*	**0.000**	.027*	**0.003**	.029*	**0.001**	.027*	**0.002**	.032*	**0.000**	.026*	**0.002**	.029*	**0.001**	.026*	**0.002**
lh_S_intrapariet.P_trans_thickness	0.003	0.696	0.003	0.704	0.004	0.645	0.004	0.629	0.003	0.729	-0.003	0.744	0.005	0.487	0.006	0.443	0.001	0.944	0.004	0.626	0.002	0.790	0.009	0.275	0.004	0.581	0.003	0.684	0.003	0.674
lh_S_oc_middle.Lunatus_thickness	0.010	0.332	0.011	0.311	0.005	0.617	0.009	0.401	0.008	0.444	0.005	0.647	0.011	0.303	0.010	0.345	0.009	0.401	0.012	0.259	0.007	0.519	0.019	0.085	0.009	0.391	0.010	0.348	0.011	0.304
lh_S_oc_sup.transversal_thickness	0.002	0.846	0.003	0.787	0.00	0.996	0.005	0.572	0.003	0.730	-0.001	0.932	0.002	0.810	0.003	0.706	0.001	0.911	0.002	0.818	0.002	0.799	0.004	0.720	0.003	0.781	0.002	0.793	0.005	0.612
lh_S_oc.temp_med.Lingual_thickness	0.010	0.310	0.011	0.254	0.010	0.350	0.010	0.305	0.008	0.388	0.011	0.284	0.011	0.242	0.013	0.181	0.002	0.822	0.009	0.343	0.008	0.427	0.015	0.161	0.012	0.230	0.011	0.254	0.009	0.348
lh_S_occipital_ant_thickness	0.000	0.984	0.004	0.710	-0.005	0.663	0.000	0.964	-0.002	0.828	0.004	0.761	-0.001	0.956	-0.001	0.945	-0.003	0.771	-0.002	0.826	-0.002	0.833	0.003	0.829	-0.002	0.833	-0.002	0.874	-0.002	0.853
lh_S_orbital_med.olfact_thickness	0.005	0.702	0.004	0.731	0.004	0.729	0.004	0.704	-0.001	0.937	-0.006	0.654	0.006	0.586	0.013	0.257	0.000	0.991	0.008	0.512	0.004	0.708	0.019	0.128	0.008	0.484	0.001	0.951	0.001	0.909

lh_S_orbital.H_Shaped_thickness	0.013	0.277	0.013	0.276	0.002	0.888	0.015	0.210	0.010	0.398	0.008	0.498	0.014	0.244	0.022	0.064	0.008	0.538	0.017	0.169	0.011	0.356	0.021	0.098	0.012	0.318	0.005	0.663	0.013	0.278
lh_S_parieto_occipital_thickness	0.003	0.701	0.008	0.370	0.007	0.488	0.008	0.385	0.005	0.598	0.004	0.662	0.010	0.275	0.008	0.406	0.000	0.979	0.008	0.380	0.006	0.534	0.006	0.529	0.006	0.473	0.008	0.394	0.005	0.554
lh_S_postcentral_thickness	.022*	**0.009**	.021*	**0.011**	.021*	**0.015**	.020*	**0.014**	.018*	**0.030**	0.009	0.298	.022*	**0.009**	.024*	**0.005**	.020*	**0.026**	.021*	**0.015**	.020*	**0.015**	.030*	**0.001**	.019*	**0.020**	.021*	**0.013**	.020*	**0.018**
lh_S_precentral.inf.part_thickness	0.014	0.135	0.016	0.104	0.012	0.240	0.015	0.114	0.014	0.141	0.004	0.706	0.014	0.135	0.018	0.060	0.011	0.285	0.016	0.103	0.011	0.253	0.018	0.069	0.012	0.193	0.013	0.183	0.014	0.142
lh_S_precentral.sup.part_thickness	.035*	**0.001**	.033*	**0.002**	.030*	**0.006**	.034*	**0.001**	.034*	**0.001**	.026*	**0.018**	.033*	**0.001**	.035*	**0.001**	.034*	**0.002**	.035*	**0.001**	.032*	**0.002**	.048*	**0.000**	.032*	**0.002**	.033*	**0.002**	.033*	**0.002**
lh_S_suborbital_thickness	0.014	0.370	0.019	0.219	0.014	0.412	0.015	0.349	0.021	0.186	0.010	0.551	0.018	0.250	0.027	0.083	0.024	0.149	0.018	0.279	0.012	0.446	0.029	0.083	0.018	0.242	0.024	0.137	0.016	0.303
lh_S_temporal_sup_thickness	-1.692E-05	0.998	0.000	0.951	-0.002	0.786	-0.002	0.771	-0.002	0.817	-0.005	0.500	-0.001	0.860	0.003	0.688	0.006	0.441	-0.003	0.692	-0.002	0.804	0.000	0.977	-0.003	0.726	-0.005	0.531	-0.002	0.754
rh_G_cuneus_thickness	.027*	**0.002**	.027*	**0.002**	.026*	**0.004**	.026*	**0.002**	.026*	**0.002**	.028*	**0.002**	.028*	**0.001**	.027*	**0.001**	.026*	**0.004**	.026*	**0.003**	.024*	**0.005**	.034*	**0.000**	.027*	**0.001**	.027*	**0.001**	.023*	**0.007**
rh_G_front_inf.Opercular_thickness	.033*	**0.002**	.034*	**0.002**	.031*	**0.005**	.035*	**0.001**	.033*	**0.002**	0.021	0.058	.036*	**0.000**	.034*	**0.001**	.040*	**0.000**	.036*	**0.001**	.030*	**0.004**	.035*	**0.002**	.031*	**0.003**	.033*	**0.002**	.034*	**0.001**
rh_G_front_inf.Triangul_thickness	0.006	0.608	0.007	0.520	0.002	0.871	0.007	0.526	0.004	0.734	-0.006	0.646	0.006	0.611	0.008	0.506	0.008	0.487	0.007	0.546	0.005	0.688	0.000	0.975	0.003	0.791	0.008	0.500	0.003	0.797
rh_G_front_middle_thickness	.021*	**0.024**	.019*	**0.043**	0.017	0.076	.022*	**0.017**	.020*	**0.025**	0.013	0.164	.021*	**0.019**	.026*	**0.005**	.023*	**0.017**	0.017	0.066	.019*	**0.038**	0.018	0.073	.018*	**0.042**	.021*	**0.023**	.021*	**0.025**
rh_G_front_sup_thickness	.033*	**0.001**	.031*	**0.002**	.031*	**0.003**	.033*	**0.001**	.033*	**0.001**	.026*	**0.013**	.034*	**0.001**	.034*	**0.001**	.034*	**0.002**	.032*	**0.002**	.031*	**0.002**	.035*	**0.001**	.032*	**0.001**	.031*	**0.002**	.033*	**0.001**
rh_G_oc.temp_med.Lingual_thickness	0.010	0.290	0.010	0.305	0.008	0.401	0.005	0.561	0.007	0.426	0.006	0.526	0.010	0.290	0.006	0.479	0.006	0.516	0.009	0.312	0.008	0.407	0.015	0.120	0.010	0.280	0.010	0.302	0.005	0.562
rh_G_oc.temp_med.Parahip_thickness	0.006	0.694	-0.001	0.947	-0.005	0.784	0.00	0.995	0.003	0.846	-0.001	0.946	0.005	0.756	0.002	0.914	-0.007	0.693	0.008	0.647	-0.002	0.909	0.022	0.197	0.000	0.982	0.002	0.912	-0.002	0.899
rh_G_occipital_middle_thickness	0.011	0.262	0.008	0.446	0.011	0.306	0.010	0.296	0.011	0.262	0.004	0.696	0.010	0.313	0.014	0.150	0.012	0.266	0.007	0.492	0.010	0.316	0.009	0.408	0.009	0.343	0.012	0.215	0.011	0.280
rh_G_occipital_sup_thickness	0.015	0.192	0.012	0.297	0.013	0.302	0.020	0.091	0.014	0.233	0.010	0.418	0.015	0.184	0.015	0.208	0.010	0.438	0.018	0.134	0.014	0.227	0.015	0.236	0.014	0.243	0.016	0.169	0.016	0.177
rh_G_orbital_thickness	0.018	0.086	0.015	0.162	0.011	0.332	0.013	0.236	0.017	0.110	0.009	0.411	0.018	0.085	0.020	0.065	0.015	0.197	0.020	0.061	0.015	0.166	0.017	0.132	0.014	0.183	0.016	0.131	0.014	0.185
rh_G_pariet_inf.Angular_thickness	0.000	0.990	-0.004	0.667	-0.001	0.894	-0.001	0.940	-0.006	0.525	-0.011	0.328	0.000	0.992	0.005	0.625	-0.006	0.583	-0.006	0.595	-0.002	0.828	0.000	0.987	-0.001	0.955	0.000	0.987	0.000	0.970
rh_G_pariet_inf.Supramar_thickness	0.009	0.373	0.010	0.323	0.006	0.547	0.010	0.300	0.007	0.455	0.000	0.989	0.012	0.224	0.016	0.103	0.014	0.162	0.007	0.499	0.008	0.429	0.011	0.292	0.011	0.272	0.012	0.229	0.009	0.335
rh_G_parietal_sup_thickness	-0.011	0.260	-0.011	0.261	-0.010	0.308	-0.011	0.244	-0.013	0.189	-0.019	0.061	-0.009	0.342	-0.004	0.648	-0.012	0.238	-0.012	0.218	-0.011	0.256	-0.005	0.657	-0.010	0.311	-0.011	0.248	-0.010	0.328
rh_G_postcentral_thickness	.025*	**0.018**	.025*	**0.021**	0.017	0.125	.024*	**0.027**	.026*	**0.018**	0.012	0.295	.025*	**0.019**	.023*	**0.034**	.027*	**0.018**	.024*	**0.029**	0.021	0.055	.025*	**0.031**	.021*	**0.047**	.024*	**0.029**	.024*	**0.028**
rh_G_precentral_thickness	.056*	**0.000**	.050*	**0.000**	.046*	**0.000**	.054*	**0.000**	.054*	**0.000**	.048*	**0.000**	.053*	**0.000**	.046*	**0.000**	.056*	**0.000**	.044*	**0.001**	.049*	**0.000**	.047*	**0.001**	.052*	**0.000**	.051*	**0.000**	.054*	**0.000**
rh_G_precuneus_thickness	0.001	0.948	0.001	0.945	0.000	0.964	-0.002	0.822	-0.001	0.950	-0.007	0.490	0.004	0.707	0.005	0.586	-0.002	0.880	-0.003	0.798	-0.003	0.787	0.003	0.755	0.001	0.958	0.000	0.970	0.000	0.970
rh_G_rectus_thickness	0.016	0.227	0.014	0.273	0.012	0.361	0.015	0.243	0.014	0.289	0.013	0.338	0.015	0.257	.026*	**0.042**	0.009	0.523	0.015	0.247	0.015	0.238	0.019	0.172	0.013	0.304	0.007	0.567	0.010	0.448

rh_G_temp_sup.G_T_transv_thickness	0.007	0.643	0.007	0.653	-0.003	0.861	0.003	0.848	0.000	0.979	0.011	0.499	0.005	0.766	0.007	0.669	0.009	0.607	0.007	0.676	0.004	0.792	-0.013	0.447	0.003	0.833	0.007	0.664	0.002	0.889
rh_G_temp_sup.Lateral_thickness	0.015	0.238	0.014	0.277	0.013	0.338	0.012	0.337	0.011	0.392	0.012	0.388	0.013	0.313	0.016	0.198	.028*	**0.040**	0.011	0.411	0.012	0.352	0.003	0.816	0.012	0.339	0.015	0.237	0.011	0.379
rh_G_temp_sup.Plan_polar_thickness	0.025	0.150	0.024	0.171	0.026	0.159	0.019	0.272	0.022	0.207	0.026	0.153	0.025	0.154	0.026	0.141	0.032	0.083	0.020	0.258	0.022	0.216	0.022	0.246	0.027	0.127	0.024	0.183	0.021	0.230
rh_G_temp_sup.Plan_tempo_thickness	0.016	0.220	0.014	0.294	0.016	0.242	0.019	0.153	0.010	0.445	0.006	0.652	0.016	0.226	0.016	0.219	0.019	0.159	0.011	0.415	0.016	0.219	0.010	0.457	0.015	0.256	0.015	0.245	0.012	0.362
rh_G_temporal_middle_thickness	-0.003	0.786	-0.006	0.605	-0.005	0.686	-0.005	0.657	-0.006	0.632	-0.013	0.313	-0.004	0.736	0.001	0.914	0.011	0.355	-0.009	0.455	-0.005	0.704	-0.009	0.467	-0.005	0.688	-0.007	0.552	-0.005	0.666
rh_G.S_cingul.Ant_thickness	-0.003	0.790	-0.003	0.803	-0.009	0.383	0.001	0.958	-0.001	0.929	-0.006	0.602	0.000	0.987	0.006	0.564	-0.006	0.595	-0.005	0.648	-0.006	0.532	0.007	0.507	-0.002	0.855	-0.004	0.704	-0.004	0.720
rh_G.S_cingul.Mid.Ant_thickness	.022*	**0.033**	.021*	**0.043**	0.018	0.091	.024*	**0.021**	.023*	**0.025**	0.020	0.063	.024*	**0.020**	.025*	**0.017**	0.016	0.132	0.019	0.071	0.019	0.068	.024*	**0.031**	.021*	**0.036**	0.019	0.062	.022*	**0.035**
rh_G.S_cingul.Mid.Post_thickness	0.012	0.195	0.011	0.227	0.008	0.394	0.013	0.154	0.010	0.258	0.004	0.675	0.012	0.187	0.016	0.085	0.007	0.473	0.011	0.227	0.009	0.306	0.013	0.177	0.010	0.287	0.009	0.305	0.012	0.191
rh_G.S_frontomargin_thickness	-.026*	**0.031**	-0.020	0.101	-.028*	**0.026**	-0.020	0.099	-0.021	0.080	-.027*	**0.032**	-.025*	**0.036**	-0.018	0.138	-0.016	0.214	-0.023	0.057	-.024*	**0.042**	-0.024	0.066	-.023*	**0.049**	-.027*	**0.026**	-.026*	**0.031**
rh_G.S_paracentral_thickness	.044*	**0.000**	.037*	**0.001**	.039*	**0.001**	.041*	**0.000**	.039*	**0.001**	.029*	**0.016**	.041*	**0.000**	.042*	**0.000**	.043*	**0.000**	.041*	**0.001**	.041*	**0.000**	.043*	**0.001**	.040*	**0.001**	.037*	**0.002**	.039*	**0.001**
rh_G.S_subcentral_thickness	0.004	0.728	0.003	0.760	-0.003	0.816	0.001	0.930	-0.002	0.853	-0.008	0.506	0.004	0.718	0.004	0.737	0.015	0.213	0.004	0.744	-0.001	0.902	0.000	0.967	-0.002	0.872	0.005	0.661	0.000	0.978
rh_G.S_transv_frontopol_thickness	-0.005	0.654	-0.003	0.782	-0.013	0.299	-0.005	0.652	-0.003	0.803	-0.002	0.846	-0.004	0.732	0.003	0.792	0.002	0.859	-0.008	0.532	-0.006	0.591	######	0.996	-0.004	0.712	-0.003	0.772	-0.007	0.584
rh_Lat_Fis.post_thickness	0.012	0.212	0.014	0.172	0.015	0.155	0.015	0.123	0.011	0.274	0.005	0.617	0.014	0.143	0.017	0.080	0.009	0.406	0.014	0.172	0.012	0.232	0.014	0.189	0.013	0.197	0.012	0.228	0.012	0.243
rh_Pole_occipital_thickness	-0.005	0.560	-0.010	0.289	-0.006	0.524	-0.006	0.538	-0.007	0.433	-0.012	0.211	-0.006	0.529	-0.009	0.343	-0.008	0.454	-0.006	0.504	-0.011	0.267	0.007	0.459	-0.008	0.385	-0.009	0.373	-0.007	0.428
rh_Pole_temporal_thickness	0.012	0.398	0.013	0.359	0.003	0.837	0.010	0.475	0.008	0.563	0.004	0.781	0.012	0.410	0.014	0.330	0.009	0.538	0.008	0.592	0.009	0.538	0.017	0.285	0.008	0.571	0.007	0.618	0.007	0.620
rh_S_calcarine_thickness	0.015	0.119	0.012	0.203	0.012	0.222	0.012	0.186	0.011	0.237	0.009	0.364	0.012	0.188	0.011	0.246	0.015	0.141	0.014	0.141	0.013	0.182	0.018	0.069	0.012	0.202	0.016	0.091	0.011	0.224
rh_S_central_thickness	.020*	**0.013**	.020*	**0.017**	.019*	**0.022**	.018*	**0.031**	.019*	**0.017**	0.016	0.070	.018*	**0.026**	.021*	**0.011**	.019*	**0.032**	.020*	**0.018**	0.015	0.057	.019*	**0.029**	.018*	**0.026**	0.016	0.056	0.016	0.054
rh_S_cingul.Marginalis_thickness	0.010	0.267	0.011	0.263	0.008	0.418	0.010	0.273	0.008	0.394	0.004	0.693	0.011	0.241	0.015	0.114	0.004	0.665	0.008	0.390	0.012	0.205	0.014	0.156	0.009	0.356	0.010	0.278	0.011	0.241
rh_S_circular_insula_ant_thickness	-0.010	0.471	-0.006	0.675	-0.009	0.560	-0.006	0.696	-0.007	0.612	-0.016	0.307	-0.005	0.712	-0.008	0.568	-0.020	0.185	-0.005	0.732	-0.005	0.727	0.003	0.858	-0.012	0.421	-0.009	0.532	-0.007	0.650
rh_S_circular_insula_sup_thickness	0.009	0.310	0.006	0.509	0.005	0.552	0.007	0.429	0.006	0.496	0.001	0.947	0.007	0.436	0.010	0.245	0.005	0.583	0.007	0.447	0.004	0.672	0.011	0.257	0.006	0.515	0.006	0.488	0.005	0.537
rh_S_front_inf_thickness	0.003	0.697	0.006	0.518	0.001	0.895	0.005	0.520	0.004	0.623	-0.001	0.896	0.004	0.667	0.010	0.263	0.008	0.349	0.003	0.750	0.001	0.895	0.004	0.697	0.002	0.840	0.002	0.791	0.001	0.876
rh_S_front_middle_thickness	0.007	0.402	0.008	0.375	-0.002	0.805	0.010	0.243	0.008	0.392	0.008	0.374	0.009	0.279	0.014	0.126	0.003	0.761	0.007	0.462	0.006	0.525	0.016	0.101	0.007	0.434	0.008	0.367	0.007	0.422
rh_S_front_sup_thickness	.029*	**0.001**	.031*	**0.000**	.028*	**0.002**	.032*	**0.000**	.027*	**0.002**	.026*	**0.005**	.031*	**0.000**	.035*	**0.000**	.023*	**0.014**	.031*	**0.001**	.029*	**0.001**	.035*	**0.000**	.029*	**0.001**	.030*	**0.001**	.029*	**0.001**
rh_S_intrapariet.P_trans_thickness	0.004	0.607	0.002	0.779	0.003	0.745	0.003	0.685	0.003	0.736	-0.003	0.709	0.004	0.616	0.008	0.335	0.000	0.978	0.002	0.817	0.002	0.752	0.010	0.229	0.003	0.704	0.001	0.861	0.001	0.898

rh_S_oc_middle.Lunatus_thickness	0.001	0.919	0.001	0.958	0.002	0.861	0.004	0.722	-0.001	0.932	-0.003	0.829	0.004	0.690	0.006	0.594	-0.003	0.782	-0.004	0.702	0.002	0.851	0.009	0.434	0.000	0.987	0.003	0.763	0.002	0.845
rh_S_oc_sup.transversal_thickness	0.008	0.409	0.006	0.527	0.009	0.359	0.009	0.353	0.009	0.355	0.008	0.421	0.009	0.326	0.010	0.278	0.006	0.567	0.010	0.283	0.008	0.406	0.007	0.464	0.007	0.447	0.011	0.228	0.008	0.370
rh_S_occipital_ant_thickness	0.002	0.854	0.006	0.596	0.004	0.717	0.007	0.566	0.004	0.745	0.006	0.607	0.003	0.762	0.009	0.457	-0.001	0.922	0.001	0.915	0.004	0.719	0.005	0.697	0.004	0.699	0.002	0.835	0.003	0.786
rh_S_orbital_med.olfact_thickness	0.011	0.383	0.008	0.520	0.003	0.821	0.011	0.402	0.010	0.439	0.004	0.748	0.012	0.366	0.017	0.200	0.006	0.662	0.011	0.423	0.009	0.482	0.024	0.090	0.012	0.349	0.007	0.574	0.006	0.614
rh_S_orbital.H_Shaped_thickness	0.019	0.110	.024*	**0.049**	0.012	0.326	0.021	0.084	0.019	0.113	0.012	0.345	0.021	0.080	.027*	**0.025**	0.017	0.171	0.017	0.174	0.013	0.280	0.024	0.058	0.020	0.093	0.018	0.148	0.019	0.113
rh_S_parieto_occipital_thickness	0.018	0.054	0.018	0.060	.022*	**0.024**	.019*	**0.040**	0.016	0.086	0.010	0.324	.020*	**0.032**	.020*	**0.032**	0.013	0.199	.020*	**0.040**	0.015	0.107	0.019	0.071	0.018	0.056	.019*	**0.041**	0.017	0.080
rh_S_postcentral_thickness	0.012	0.170	0.011	0.211	0.009	0.320	0.009	0.303	0.010	0.235	0.001	0.871	0.012	0.138	0.011	0.194	0.009	0.311	0.011	0.203	0.009	0.278	.019*	**0.034**	0.009	0.311	0.010	0.238	0.011	0.214
rh_S_precentral.inf.part_thickness	.019*	**0.046**	0.018	0.064	0.016	0.105	.021*	**0.024**	.020*	**0.035**	0.012	0.216	.021*	**0.023**	.022*	**0.018**	.020*	**0.048**	0.017	0.074	0.016	0.088	.023*	**0.022**	0.017	0.068	0.018	0.051	.022*	**0.020**
rh_S_precentral.sup.part_thickness	.044*	**0.000**	.043*	**0.000**	.038*	**0.001**	.046*	**0.000**	.043*	**0.000**	.042*	**0.001**	.046*	**0.000**	.043*	**0.000**	.039*	**0.001**	.044*	**0.000**	.041*	**0.000**	.053*	**0.000**	.043*	**0.000**	.043*	**0.000**	.047*	**0.000**
rh_S_temporal_sup_thickness	-0.011	0.152	-0.011	0.165	-0.015	0.068	-0.010	0.173	-0.014	0.072	-.018*	**0.032**	-0.012	0.132	-0.009	0.246	-0.008	0.346	-0.014	0.077	-0.012	0.128	-0.010	0.218	-0.012	0.122	-0.012	0.132	-0.013	0.093

Table S13: Leave one out analyses for cortical thickness (main FND cohort).

Each site heading shows the effect of removing that site and running the same MANCOVA with the fourteen remaining sites. Those boxes in bold are significant. Boxes coded green are those that were significant in the main analysis and remain significant after removal of that site; boxes red are regions significant in the main analysis but became no longer significant after the removal of that site; boxes coded in blue were not significant in the main analysis but are significant after the removal of that site.

	Site 1		Site 2		Site 3		Site 4		Site 5		Site 6		Site 7		Site 8		Site 9		Site 10		Site 11		Site 12		Site 13		Site 14		Site 15	
	HC - FND	P	HC - FND	P	HC - FND	P	HC - FND	P	HC - FND	P	HC - FND	P	HC - FND	P	HC - FND	P	HC - FND	P	HC - FND	P	HC - FND	P	HC - FND	P	HC - FND	P	HC - FND	P	HC - FND	P
Left.Cerebellum.White.Matter	-44.156	0.738	27.804	0.835	-16.113	0.907	-32.213	0.808	-32.643	0.805	-72.931	0.603	-72.931	0.603	-36.324	0.785	26.749	0.846	-17.299	0.898	-81.964	0.533	31.459	0.825	-39.390	0.764	-39.506	0.768	-49.549	0.706
Left.Cerebellum.Cortex	544.751	0.118	780.883*	**0.027**	547.320	0.132	464.292	0.184	485.057	0.164	286.738	0.438	286.738	0.438	639.179	0.069	342.714	0.350	300.827	0.396	538.828	0.123	529.133	0.158	507.665	0.145	570.062	0.106	569.727	0.104
Left.Thalamus.Proper	49.867	0.224	34.380	0.395	46.116	0.284	46.280	0.260	40.496	0.324	42.429	0.332	42.429	0.332	63.851	0.120	48.829	0.261	41.798	0.318	36.981	0.364	70.945	0.104	43.560	0.283	44.964	0.279	59.828	0.146
Left.Caudate	42.691	0.089	32.801	0.191	53.196*	**0.043**	50.030*	**0.046**	47.666	0.056	32.798	0.215	32.798	0.215	41.624	0.099	49.168	0.062	41.091	0.109	46.119	0.064	51.874	0.053	44.347	0.075	46.326	0.066	44.967	0.074
Left.Putamen	65.792*	**0.050**	54.551	0.105	68.600	0.050	76.672*	**0.023**	66.707*	**0.046**	44.080	0.214	44.080	0.214	42.417	0.207	52.190	0.136	54.538	0.111	58.365	0.082	85.102*	**0.018**	65.092	0.052	68.024*	**0.045**	72.213*	**0.032**
Left.Pallidum	35.508*	**0.011**	32.076*	**0.022**	31.852*	**0.027**	36.387*	**0.009**	29.589*	**0.033**	27.773	0.059	27.773	0.059	33.056*	**0.018**	28.701*	**0.049**	33.376*	**0.019**	31.849*	**0.022**	32.715*	**0.029**	35.729*	**0.010**	32.289*	**0.021**	34.450*	**0.013**
Left.Hippocampus	55.164*	**0.013**	49.252*	**0.027**	61.915*	**0.008**	50.874*	**0.022**	53.937*	**0.015**	52.881*	**0.022**	52.881*	**0.022**	53.402*	**0.017**	53.123*	**0.026**	48.665*	**0.032**	49.283*	**0.026**	45.226	0.059	50.257*	**0.023**	42.023	0.063	56.923*	**0.011**
Left.Amygdala	12.238	0.296	10.514	0.376	15.087	0.215	12.763	0.279	9.775	0.404	10.556	0.386	10.556	0.386	6.898	0.560	10.507	0.401	10.992	0.358	8.927	0.446	3.558	0.777	11.815	0.310	9.164	0.438	11.385	0.334
Left.Accumbens.area	4.252	0.472	2.464	0.677	2.651	0.664	4.358	0.464	4.931	0.402	4.645	0.455	4.645	0.455	-0.220	0.970	4.736	0.444	2.290	0.704	3.982	0.499	6.136	0.333	4.661	0.427	5.089	0.393	5.328	0.368
Left.VentralDC	-5.226	0.835	-18.834	0.448	-9.646	0.711	-3.982	0.873	-8.078	0.747	-15.313	0.567	-15.313	0.567	-11.584	0.644	1.415	0.957	-14.086	0.582	-7.324	0.768	2.564	0.925	-7.363	0.767	-12.512	0.622	-6.899	0.784
Right.Cerebellum.White.Matter	-283.390*	**0.044**	-212.652	0.136	-289.841*	**0.049**	-279.400*	**0.048**	-289.855*	**0.041**	-286.375	0.052	-286.375	0.052	-242.740	0.088	-253.684	0.087	-281.971	0.050	-318.737*	**0.024**	-301.590*	**0.050**	-257.970	0.065	-270.643	0.057	-283.799*	**0.044**
Right.Cerebellum.Cortex	543.084	0.129	752.774*	**0.037**	501.930	0.177	454.763	0.204	494.325	0.166	262.199	0.490	262.199	0.490	673.536	0.061	396.522	0.291	299.726	0.408	493.357	0.170	580.975	0.132	458.223	0.199	527.506	0.144	549.615	0.125
Right.Thalamus.Proper	71.330	0.056	65.945	0.076	77.641*	**0.047**	75.201*	**0.044**	70.504	0.059	63.331	0.108	63.331	0.108	82.251*	**0.027**	73.567	0.061	65.493	0.088	66.134	0.074	87.226*	**0.028**	68.031	0.065	77.676*	**0.040**	82.439*	**0.027**
Right.Caudate	39.619	0.117	30.966	0.220	54.112*	**0.040**	45.954	0.069	41.897	0.095	32.025	0.229	32.025	0.229	37.764	0.136	45.851	0.083	37.375	0.146	39.031	0.119	45.000	0.098	41.578	0.098	42.498	0.094	42.575	0.092
Right.Putamen	63.314	0.055	60.072	0.070	68.558*	**0.046**	71.055*	**0.032**	66.321*	**0.043**	47.001	0.181	47.001	0.181	44.526	0.180	42.367	0.216	52.837	0.117	61.744	0.062	79.217*	**0.025**	65.043*	**0.048**	62.809	0.061	72.303*	**0.029**
Right.Pallidum	17.840	0.194	17.498	0.207	12.696	0.374	20.004	0.147	14.392	0.294	12.978	0.375	12.978	0.375	19.475	0.160	10.964	0.443	15.911	0.261	13.813	0.315	10.080	0.495	16.583	0.227	13.985	0.314	17.243	0.213
Right.Hippocampus	76.627*	**0.001**	70.320*	**0.002**	87.864*	**0.000**	78.089*	**0.001**	80.261*	**0.000**	74.781*	**0.002**	74.781*	**0.002**	72.050*	**0.002**	73.259*	**0.003**	73.788*	**0.002**	75.720*	**0.001**	69.329*	**0.005**	73.170*	**0.002**	67.814*	**0.004**	80.251*	**0.001**
Right.Amygdala	8.029	0.476	8.594	0.450	8.223	0.483	11.073	0.325	10.954	0.328	9.087	0.438	9.087	0.438	7.822	0.489	7.633	0.521	12.526	0.282	9.903	0.379	4.279	0.721	7.476	0.504	7.153	0.529	10.769	0.341
Right.Accumbens.area	14.864*	**0.010**	13.866*	**0.016**	15.041*	**0.011**	16.094*	**0.005**	15.688*	**0.006**	12.577*	**0.037**	12.577*	**0.037**	11.888*	**0.039**	17.503*	**0.004**	13.643*	**0.020**	15.435*	**0.007**	13.655*	**0.025**	15.637*	**0.006**	15.523*	**0.008**	17.662*	**0.002**
Right.VentralDC	26.359	0.243	17.131	0.447	21.736	0.355	29.757	0.185	18.970	0.397	19.165	0.424	19.165	0.424	14.668	0.516	31.218	0.187	21.590	0.349	25.446	0.258	23.786	0.326	22.399	0.317	19.974	0.382	23.964	0.289

Table S14: Leave one out analyses for subcortical volumes (main FND cohort).

Each site heading shows the effect of removing that site and running the same MANCOVA with the fourteen remaining sites. Those boxes in bold are significant. Boxes coded green are those that were significant in the main analysis and remain significant after removal of that site; boxes red are regions significant in the main analysis but became no longer significant after the removal of that site; boxes coded in blue were not significant in the main analysis but are significant after the removal of that site.

	F	*p*	***ηp²***	*Observed Power[a]*	*Mean Difference (I-J)*
lh_G_cingul.Post.dorsal_thickness	*0.171719*	*0.678685*	*0.000187*	*0.069852*	*0.004545*
lh_G_cuneus_thickness	*5.027424*	*0.025187*	*0.005435*	*0.610231*	*0.019000*
lh_G_front_inf.Opercular_thickness	*3.078183*	*0.079682*	*0.003335*	*0.417982*	*0.018290*
lh_G_front_inf.Orbital_thickness	*0.157554*	*0.691511*	*0.000171*	*0.068198*	*0.005690*
lh_G_front_inf.Triangul_thickness	*0.543499*	*0.461174*	*0.00059*	*0.114073*	*0.008268*
lh_G_front_middle_thickness	*2.513832*	*0.113195*	*0.002725*	*0.353614*	*0.014809*
lh_G_front_sup_thickness	*10.89965*	*0.000999*	*0.011709*	*0.90956*	*0.033000*
lh_G_Ins_lg.S_cent_ins_thickness	*0.005219*	*0.942424*	*5.67E-06*	*0.050597*	*0.001221*
lh_G_oc.temp_med.Parahip_thickness	*0.687191*	*0.407336*	*0.000746*	*0.131498*	*-0.013729*
lh_G_occipital_middle_thickness	*1.456751*	*0.227758*	*0.001581*	*0.226119*	*0.011489*
lh_G_occipital_sup_thickness	*0.001855*	*0.965659*	*2.02E-06*	*0.050212*	*-0.000501*
lh_G_orbital_thickness	*0.19115*	*0.662064*	*0.000208*	*0.072124*	*0.004533*
lh_G_pariet_inf.Angular_thickness	*0.005281*	*0.942083*	*5.74E-06*	*0.050604*	*0.000710*
lh_G_pariet_inf.Supramar_thickness	*0.059878*	*0.806743*	*6.51E-05*	*0.056873*	*-0.002424*
lh_G_parietal_sup_thickness	*0.431724*	*0.511309*	*0.000469*	*0.10063*	*-0.006047*
lh_G_postcentral_thickness	*2.336509*	*0.126716*	*0.002533*	*0.332753*	*0.015488*
lh_G_precentral_thickness	*4.735707*	*0.029797*	*0.005121*	*0.584718*	*0.027000*
lh_G_precuneus_thickness	*0.842289*	*0.358984*	*0.000915*	*0.150446*	*0.011493*
lh_G_rectus_thickness	*0.023127*	*0.879162*	*2.51E-05*	*0.052648*	*0.001763*
lh_G_temp_sup.G_T_transv_thickness	*0.065158*	*0.798578*	*7.08E-05*	*0.057482*	*0.003687*
lh_G_temp_sup.Lateral_thickness	*1.065163*	*0.302312*	*0.001156*	*0.177842*	*0.013900*
lh_G_temporal_middle_thickness	*0.212791*	*0.644698*	*0.000231*	*0.074661*	*0.005536*
lh_G.S_cingul.Ant_thickness	*0.067288*	*0.795385*	*7.31E-05*	*0.057727*	*0.002571*
lh_G.S_cingul.Mid.Ant_thickness	*1.777751*	*0.182756*	*0.001929*	*0.265489*	*0.014481*
lh_G.S_cingul.Mid.Post_thickness	*1.65631*	*0.198426*	*0.001797*	*0.250637*	*0.011715*

lh_G.S_frontomargin_thickness	*1.072316*	*0.300695*	*0.001164*	*0.178723*	*0.011333*
lh_G.S_paracentral_thickness	*6.798733*	*0.00927*	*0.007336*	*0.740458*	*0.030000*
lh_G.S_subcentral_thickness	*0.027825*	*0.867557*	*3.02E-05*	*0.053187*	*-0.001834*
lh_Lat_Fis.post_thickness	*3.456458*	*0.063324*	*0.003743*	*0.459148*	*0.018880*
lh_Pole_occipital_thickness	*1.679143*	*0.195363*	*0.001822*	*0.253434*	*-0.013099*
lh_Pole_temporal_thickness	*0.514141*	*0.473534*	*0.000559*	*0.110532*	*0.010234*
lh_S_central_thickness	*5.820575*	*0.016034*	*0.006287*	*0.673688*	*0.019000*
lh_S_circular_insula_ant_thickness	*0.591503*	*0.442036*	*0.000643*	*0.119878*	*0.010705*
lh_S_circular_insula_inf_thickness	*0.350079*	*0.554214*	*0.00038*	*0.090885*	*0.007121*
lh_S_circular_insula_sup_thickness	*0.057342*	*0.8108*	*6.23E-05*	*0.056581*	*0.002031*
lh_S_front_inf_thickness	*1.650324*	*0.199238*	*0.001791*	*0.249903*	*0.010858*
lh_S_front_middle_thickness	*2.388182*	*0.1226*	*0.002589*	*0.33886*	*0.014884*
lh_S_front_sup_thickness	*11.40875*	*0.000762*	*0.012249*	*0.921347*	*0.028000*
lh_S_intrapariet.P_trans_thickness	*0.345675*	*0.556716*	*0.000376*	*0.090361*	*0.004600*
lh_S_oc_middle.Lunatus_thickness	*1.332811*	*0.248606*	*0.001447*	*0.210846*	*0.011859*
lh_S_oc_sup.transversal_thickness	*0.109299*	*0.741019*	*0.000119*	*0.062586*	*0.003022*
lh_S_oc.temp_med.Lingual_thickness	*1.111085*	*0.292123*	*0.001206*	*0.183501*	*0.010150*
lh_S_occipital_ant_thickness	*0.003499*	*0.952845*	*3.8E-06*	*0.0504*	*-0.000647*
lh_S_orbital_med.olfact_thickness	*0.170658*	*0.679624*	*0.000185*	*0.069728*	*0.004796*
lh_S_orbital.H_Shaped_thickness	*1.073778*	*0.300366*	*0.001166*	*0.178903*	*0.012111*
lh_S_parieto_occipital_thickness	*0.494122*	*0.482272*	*0.000537*	*0.108121*	*0.006299*
lh_S_postcentral_thickness	*6.509854*	*0.010889*	*0.007026*	*0.72201*	*0.021000*
lh_S_precentral.inf.part_thickness	*2.055914*	*0.151956*	*0.00223*	*0.29923*	*0.013353*
lh_S_precentral.sup.part_thickness	*11.02846*	*0.000933*	*0.011845*	*0.912686*	*0.034000*
lh_S_suborbital_thickness	*1.701393*	*0.192432*	*0.001846*	*0.256158*	*0.020262*
lh_S_temporal_sup_thickness	*0.055746*	*0.813403*	*6.06E-05*	*0.056397*	*-0.001772*
rh_G_cuneus_thickness	*12.63146*	*0.000399*	*0.013544*	*0.944128*	*0.030000*
rh_G_front_inf.Opercular_thickness	*9.812313*	*0.001788*	*0.010553*	*0.878844*	*0.032000*
rh_G_front_inf.Triangul_thickness	*0.191857*	*0.661478*	*0.000208*	*0.072207*	*0.004927*

rh_G_front_middle_thickness	*4.564174*	*0.032911*	*0.004937*	*0.569166*	*0.019000*
rh_G_front_sup_thickness	*9.983191*	*0.001631*	*0.010735*	*0.884221*	*0.031000*
rh_G_oc.temp_med.Lingual_thickness	*1.416655*	*0.234262*	*0.001537*	*0.221181*	*0.010605*
rh_G_oc.temp_med.Parahip_thickness	*0.022124*	*0.881791*	*2.4E-05*	*0.052533*	*-0.002348*
rh_G_occipital_middle_thickness	*1.016652*	*0.31358*	*0.001104*	*0.171867*	*0.009792*
rh_G_occipital_sup_thickness	*1.763468*	*0.184522*	*0.001913*	*0.263745*	*0.015267*
rh_G_orbital_thickness	*1.983881*	*0.15932*	*0.002152*	*0.290535*	*0.014608*
rh_G_pariet_inf.Angular_thickness	*0.158715*	*0.690435*	*0.000172*	*0.068334*	*-0.003997*
rh_G_pariet_inf.Supramar_thickness	*0.540644*	*0.462353*	*0.000587*	*0.113728*	*0.007026*
rh_G_parietal_sup_thickness	*1.275947*	*0.258948*	*0.001385*	*0.203834*	*-0.010865*
rh_G_postcentral_thickness	*4.236657*	*0.039841*	*0.004584*	*0.538349*	*0.022000*
rh_G_precentral_thickness	*13.78948*	*0.000217*	*0.014767*	*0.959904*	*0.046000*
rh_G_precuneus_thickness	*0.031138*	*0.859972*	*3.38E-05*	*0.053567*	*-0.001688*
rh_G_rectus_thickness	*1.696964*	*0.193011*	*0.001841*	*0.255616*	*0.016574*
rh_G_temp_sup.G_T_transv_thickness	*0.001548*	*0.968629*	*1.68E-06*	*0.050177*	*0.000602*
rh_G_temp_sup.Lateral_thickness	*0.739162*	*0.390153*	*0.000803*	*0.137833*	*0.010652*
rh_G_temp_sup.Plan_polar_thickness	*1.267245*	*0.260578*	*0.001376*	*0.20276*	*0.019214*
rh_G_temp_sup.Plan_tempo_thickness	*0.920657*	*0.337555*	*0.001*	*0.160062*	*0.012283*
rh_G_temporal_middle_thickness	*0.221879*	*0.637723*	*0.000241*	*0.075728*	*-0.005471*
rh_G.S_cingul.Ant_thickness	*1.07E-05*	*0.997385*	*1.17E-08*	*0.050001*	*0.000033*
rh_G.S_cingul.Mid.Ant_thickness	*4.981192*	*0.025864*	*0.005385*	*0.606266*	*0.022000*
rh_G.S_cingul.Mid.Post_thickness	*1.777948*	*0.182732*	*0.001929*	*0.265513*	*0.011872*
rh_G.S_frontomargin_thickness	*2.806712*	*0.09421*	*0.003041*	*0.38743*	*-0.019597*
rh_G.S_paracentral_thickness	*11.97969*	*0.000563*	*0.012854*	*0.93288*	*0.039000*
rh_G.S_subcentral_thickness	*4.12E-05*	*0.994882*	*4.47E-08*	*0.050005*	*-0.000070*
rh_G.S_transv_frontopol_thickness	*0.017505*	*0.89477*	*1.9E-05*	*0.052004*	*-0.001549*
rh_Lat_Fis.post_thickness	*1.620478*	*0.203347*	*0.001758*	*0.246243*	*0.012433*
rh_Pole_occipital_thickness	*0.122807*	*0.72609*	*0.000133*	*0.064154*	*-0.003220*
rh_Pole_temporal_thickness	*0.087805*	*0.767053*	*9.54E-05*	*0.060097*	*0.004108*

rh_S_calcarine_thickness	*2.367541*	*0.124226*	*0.002567*	*0.336423*	*0.014244*
rh_S_central_thickness	*5.343269*	*0.021023*	*0.005774*	*0.63653*	*0.019000*
rh_S_cingul.Marginalis_thickness	*1.376335*	*0.24103*	*0.001494*	*0.216212*	*0.010806*
rh_S_circular_insula_ant_thickness	*0.370607*	*0.542824*	*0.000403*	*0.093329*	*-0.008731*
rh_S_circular_insula_sup_thickness	*0.578345*	*0.447157*	*0.000628*	*0.118285*	*0.006611*
rh_S_front_inf_thickness	*0.285395*	*0.593316*	*0.00031*	*0.083213*	*0.004457*
rh_S_front_middle_thickness	*1.23622*	*0.266491*	*0.001342*	*0.198933*	*0.009647*
rh_S_front_sup_thickness	*12.62113*	*0.000401*	*0.013533*	*0.943964*	*0.030000*
rh_S_intrapariet.P_trans_thickness	*0.27368*	*0.601*	*0.000297*	*0.081829*	*0.004055*
rh_S_oc_middle.Lunatus_thickness	*0.103064*	*0.748256*	*0.000112*	*0.061863*	*0.003531*
rh_S_oc_sup.transversal_thickness	*0.898959*	*0.34331*	*0.000976*	*0.157397*	*0.008809*
rh_S_occipital_ant_thickness	*0.132893*	*0.715535*	*0.000144*	*0.065326*	*0.004112*
rh_S_orbital_med.olfact_thickness	*1.013971*	*0.314218*	*0.001101*	*0.171537*	*0.012667*
rh_S_orbital.H_Shaped_thickness	*2.431152*	*0.119289*	*0.002636*	*0.343921*	*0.018464*
rh_S_parieto_occipital_thickness	*3.967962*	*0.046669*	*0.004294*	*0.511978*	*0.019000*
rh_S_postcentral_thickness	*1.804982*	*0.179443*	*0.001958*	*0.26881*	*0.011186*
rh_S_precentral.inf.part_thickness	*4.1928*	*0.04088*	*0.004537*	*0.534111*	*0.019000*
rh_S_precentral.sup.part_thickness	*14.99608*	*0.000115*	*0.016039*	*0.971835*	*0.044000*
rh_S_temporal_sup_thickness	*2.662721*	*0.103067*	*0.002886*	*0.37091*	*-0.012408*

Table S15: cortical thickness differences FND vs HCs (with inclusion of Euler as covariate)

	F	Sig (FDR)	Partial Eta Squared	Observed Power	Mean Difference (I-J)
lh_G_cingul.Post.ventral_area	4.757178	0.282518	0.005144	0.586636	-0.00002673000
lh_G_cuneus_area	1.658106	0.656054	0.001799	0.250857	0.00008851054
lh_G_front_inf.Triangul_area	0.046387	0.948226	5.04E-05	0.055319	0.00001123353
lh_G_front_middle_area	0.02522	0.953296	2.74E-05	0.052888	0.00001912454
lh_G_front_sup_area	1.026936	0.737376	0.001115	0.173134	-0.00014873469
lh_G_Ins_lg.S_cent_ins_area	1.988479	0.586479	0.002157	0.291091	-0.00003073010
lh_G_insular_short_area	0.32677	0.802431	0.000355	0.088115	0.00001297232
lh_G_oc.temp_med.Lingual_area	0.037372	0.948226	4.06E-05	0.054283	0.00002000821
lh_G_occipital_middle_area	0.493995	0.771725	0.000537	0.108106	0.00005361842
lh_G_occipital_sup_area	0.967444	0.737376	0.00105	0.165813	0.00004624097
lh_G_pariet_inf.Angular_area	0.631514	0.771725	0.000686	0.124729	0.00006677061
lh_G_pariet_inf.Supramar_area	0.043586	0.948226	4.74E-05	0.054997	0.00002166572
lh_G_parietal_sup_area	0.044053	0.948226	4.79E-05	0.055051	0.00002086166
lh_G_postcentral_area	14.05874	0.018072	0.015051	0.962919	0.00023144196
lh_G_precentral_area	0.026901	0.953296	2.92E-05	0.053081	0.00001034885
lh_G_precuneus_area	3.638333	0.355408	0.003939	0.478318	-0.00015573705
lh_G_temp_sup.G_T_transv_area	0.170498	0.8701	0.000185	0.069709	0.00001020561
lh_G_temp_sup.Plan_tempo_area	1.868681	0.611414	0.002027	0.276566	-0.00006737758
lh_G_temporal_inf_area	4.080954	0.355408	0.004416	0.523186	0.00000000000
lh_G_temporal_middle_area	0.41743	0.802431	0.000454	0.098919	0.00004671899
lh_G.S_cingul.Ant_area	0.016033	0.96512	1.74E-05	0.051835	0.00000710388
lh_G.S_cingul.Mid.Ant_area	1.427967	0.737376	0.00155	0.222574	-0.00004956316
lh_G.S_cingul.Mid.Post_area	3.784587	0.355408	0.004097	0.493428	-0.00007084893

lh_G.S_occipital_inf_area	0.117313	0.900979	0.000127	0.063516	0.00002264005
lh_G.S_paracentral_area	0.723904	0.771725	0.000786	0.135972	-0.00003354283
lh_Lat_Fis.post_area	5.015323	0.282518	0.005422	0.609196	-0.00007242000
lh_Pole_occipital_area	0.004094	0.979612	4.45E-06	0.050468	-0.00000390785
lh_S_calcarine_area	0.545526	0.771725	0.000593	0.114318	-0.00008062934
lh_S_central_area	0.00102	0.984782	1.11E-06	0.050117	0.00000224855
lh_S_cingul.Marginalis_area	3.567517	0.355408	0.003863	0.470904	-0.00006071904
lh_S_circular_insula_ant_area	0.785674	0.751286	0.000853	0.143516	0.00001666086
lh_S_circular_insula_inf_area	4.775765	0.282518	0.005164	0.588291	-0.00007518000
lh_S_circular_insula_sup_area	4.863355	0.282518	0.005258	0.596027	-0.00007843000
lh_S_front_inf_area	1.174954	0.737376	0.001275	0.191377	-0.00008612044
lh_S_front_middle_area	0.086535	0.922435	9.41E-05	0.05995	-0.00001791235
lh_S_front_sup_area	0.174037	0.8701	0.000189	0.070123	-0.00003866801
lh_S_intrapariet.P_trans_area	0.107522	0.902956	0.000117	0.06238	-0.00003358733
lh_S_oc_middle.Lunatus_area	1.084236	0.737376	0.001177	0.180192	0.00006252606
lh_S_oc.temp_lat_area	0.896617	0.737376	0.000974	0.15711	0.00004190493
lh_S_orbital_lateral_area	3.090924	0.42043	0.003348	0.419396	0.00004055736
lh_S_parieto_occipital_area	0.000191	0.988974	2.08E-07	0.050022	0.00000106859
lh_S_pericallosal_area	0.885224	0.737376	0.000961	0.155711	0.00005086302
lh_S_postcentral_area	1.308912	0.737376	0.001421	0.207899	0.00011182582
lh_S_precentral.sup.part_area	0.505388	0.771725	0.000549	0.109478	-0.00004080514
lh_S_subparietal_area	0.260088	0.802431	0.000283	0.080225	-0.00002451244
lh_S_temporal_inf_area	0.598676	0.771725	0.00065	0.120747	0.00005135751
lh_S_temporal_sup_area	0.271224	0.802431	0.000295	0.081539	0.00007775176
lh_S_temporal_transverse_area	2.681176	0.453355	0.002906	0.373039	0.00003995923
rh_G_cingul.Post.ventral_area	3.212019	0.414651	0.003479	0.432741	-0.00002304609
rh_G_cuneus_area	2.649978	0.453355	0.002872	0.369438	-0.00011340495

rh_G_front_inf.Orbital_area	0.970762	0.737376	0.001054	0.166221	-0.00002017447
rh_G_front_inf.Triangul_area	0.400754	0.802431	0.000435	0.096926	-0.00003222711
rh_G_front_middle_area	0.122501	0.900979	0.000133	0.064118	0.00004277665
rh_G_front_sup_area	4.781246	0.282518	0.00517	0.588778	0.00000000000
rh_G_oc.temp_med.Lingual_area	0.637228	0.771725	0.000692	0.125423	-0.00007869130
rh_G_occipital_middle_area	0.804681	0.751286	0.000874	0.145841	0.00007492576
rh_G_occipital_sup_area	0.002104	0.983924	2.29E-06	0.050241	0.00000240274
rh_G_pariet_inf.Angular_area	1.134703	0.737376	0.001232	0.186413	0.00010961323
rh_G_pariet_inf.Supramar_area	0.660968	0.771725	0.000718	0.128307	0.00006679435
rh_G_parietal_sup_area	3.622509	0.355408	0.003922	0.476667	0.00015810547
rh_G_postcentral_area	1.362136	0.737376	0.001478	0.214462	0.00006343538
rh_G_precentral_area	2.030143	0.586479	0.002202	0.296123	-0.00008620717
rh_G_precuneus_area	0.357834	0.802431	0.000389	0.091807	-0.00004483262
rh_G_temp_sup.G_T_transv_area	0.565069	0.771725	0.000614	0.116679	0.00001233325
rh_G_temp_sup.Lateral_area	0.340353	0.802431	0.00037	0.089728	-0.00002749073
rh_G_temp_sup.Plan_tempo_area	0.921606	0.737376	0.001001	0.160178	-0.00003029179
rh_G_temporal_inf_area	2.259413	0.555746	0.00245	0.323602	0.00011894818
rh_G_temporal_middle_area	0.387454	0.802431	0.000421	0.095338	0.00004390551
rh_G.S_cingul.Ant_area	0.53494	0.771725	0.000581	0.11304	0.00004568349
rh_G.S_cingul.Mid.Ant_area	0.276354	0.802431	0.0003	0.082145	0.00002087459
rh_G.S_cingul.Mid.Post_area	3.912323	0.355408	0.004235	0.506396	-0.00007414000
rh_G.S_occipital_inf_area	0.347659	0.802431	0.000378	0.090597	0.00002686887
rh_G.S_paracentral_area	7.306291	0.200267	0.007879	0.770421	-0.00009828000
rh_G.S_transv_frontopol_area	1.202043	0.737376	0.001305	0.194718	0.00004056004
rh_Lat_Fis.post_area	9.049874	0.129536	0.009741	0.852034	-0.00009774000
rh_Pole_occipital_area	0.014312	0.96512	1.56E-05	0.051638	-0.00001143966
rh_S_calcarine_area	1.086549	0.737376	0.00118	0.180477	-0.00010387227
rh_S_central_area	0.004658	0.979612	5.06E-06	0.050533	0.00000492166

rh_S_cingul.Marginalis_area	6.988202	0.200267	0.007539	0.752002	0.00000000000
rh_S_circular_insula_ant_area	0.291988	0.802431	0.000317	0.083993	0.00001239222
rh_S_circular_insula_inf_area	5.341696	0.282518	0.005773	0.636403	-0.00006599000
rh_S_circular_insula_sup_area	2.012682	0.586479	0.002183	0.294016	-0.00004748146
rh_S_collat_transv_post_area	2.852341	0.439583	0.003091	0.392621	-0.00004624681
rh_S_front_inf_area	0.509364	0.771725	0.000553	0.109956	0.00005597181
rh_S_front_middle_area	0.036051	0.948226	3.92E-05	0.054131	0.00001837027
rh_S_front_sup_area	3.007595	0.42043	0.003258	0.410116	-0.00015902681
rh_S_intrapariet.P_trans_area	0.117373	0.900979	0.000128	0.063523	0.00003872530
rh_S_oc_middle.Lunatus_area	1.08667	0.737376	0.00118	0.180492	0.00005846272
rh_S_occipital_ant_area	0.320704	0.802431	0.000348	0.087395	-0.00002346639
rh_S_orbital_lateral_area	0.074484	0.930345	8.1E-05	0.058558	0.00000730416
rh_S_postcentral_area	1.664969	0.656054	0.001806	0.251698	0.00011240760
rh_S_precentral.sup.part_area	0.862347	0.737376	0.000936	0.152905	-0.00005595155
rh_S_subparietal_area	0.008419	0.977843	9.15E-06	0.050963	0.00000523127
rh_S_temporal_inf_area	0.282975	0.802431	0.000307	0.082927	0.00002781709
rh_S_temporal_sup_area	0.504679	0.771725	0.000548	0.109392	-0.00010702623
rh_S_temporal_transverse_area	0.881594	0.737376	0.000957	0.155266	0.00001690235

Table S16: cortical surface area differences FND vs HCs (with inclusion of Euler as covariate)

	F	Sig.	Partial Eta Squared	Observed Power	Mean Difference (I-J)
lh_G.S_cingul.Ant_thickness	0.860521	0.615347	0.001411	0.152569	0.011433
lh_G.S_cingul.Mid.Post_thickness	1.299101	0.514202	0.002129	0.206519	0.013404
lh_G.S_subcentral_thickness	0.543177	0.706639	0.000891	0.113964	-0.00986
lh_G_cingul.Post.dorsal_thickness	1.0811	0.544704	0.001772	0.179663	0.014213
lh_G_cingul.Post.ventral_thickness	0.136582	0.874315	0.000224	0.065738	-0.00766
lh_G_cuneus_thickness	4.195609	0.205111	0.006842	0.53395	0.022
lh_G_front_inf.Opercular_thickness	4.932616	0.167751	0.008034	0.601611	0.03
lh_G_front_inf.Orbital_thickness	0.578206	0.702029	0.000949	0.118194	0.013354
lh_G_front_inf.Triangul_thickness	0.504199	0.720085	0.000827	0.10927	0.00965
lh_G_front_middle_thickness	3.897952	0.212448	0.00636	0.504528	0.023
lh_G_front_sup_thickness	8.405981	0.048648	0.013615	0.825047	0.035
lh_G_Ins_lg.S_cent_ins_thickness	2.254611	0.30841	0.003688	0.322744	0.031255
lh_G_insular_short_thickness	0.016992	0.946592	2.79E-05	0.051943	-0.00264
lh_G_oc.temp_med.Lingual_thickness	2.929876	0.271231	0.004788	0.401038	0.017607
lh_G_oc.temp_med.Parahip_thickness	0.184566	0.847666	0.000303	0.07133	0.008715
lh_G_occipital_middle_thickness	3.201164	0.255388	0.005229	0.431177	0.021105
lh_G_occipital_sup_thickness	0.000172	0.989545	2.82E-07	0.05002	-0.00019
lh_G_orbital_thickness	0.928582	0.592569	0.001522	0.160914	0.0119
lh_G_pariet_inf.Angular_thickness	0.237559	0.847666	0.00039	0.077541	0.005931
lh_G_pariet_inf.Supramar_thickness	0.267883	0.847666	0.00044	0.081111	0.006414
lh_G_parietal_sup_thickness	0.280076	0.847666	0.00046	0.082549	0.00599
lh_G_postcentral_thickness	2.094115	0.322445	0.003427	0.303559	0.017944
lh_G_precentral_thickness	2.025866	0.330798	0.003316	0.295346	0.021712
lh_G_precuneus_thickness	0.947351	0.592569	0.001553	0.163218	0.014524
lh_G_rectus_thickness	0.149959	0.867629	0.000246	0.067294	0.005406

lh_G_temp_sup.G_T_transv_thickness	1.127343	0.543838	0.001848	0.185357	-0.0188
lh_G_temp_sup.Lateral_thickness	0.0026	0.980276	4.27E-06	0.050297	0.000852
lh_G_temp_sup.Plan_polar_thickness	3.408039	0.238264	0.005565	0.453586	0.041036
lh_G_temp_sup.Plan_tempo_thickness	0.039526	0.92427	6.49E-05	0.054526	0.003198
lh_G_temporal_inf_thickness	0.698876	0.646093	0.001146	0.13283	0.011511
lh_G_temporal_middle_thickness	0.074838	0.908556	0.000123	0.058589	0.004108
lh_Lat_Fis.ant.Horizont_thickness	0.018504	0.946592	3.04E-05	0.052116	0.002638
lh_Lat_Fis.post_thickness	5.242957	0.156523	0.008536	0.627865	0.028
lh_Pole_occipital_thickness	0.002162	0.980276	3.55E-06	0.050247	-0.00058
lh_Pole_temporal_thickness	2.760963	0.271231	0.004513	0.381867	0.029226
lh_S_central_thickness	2.844447	0.271231	0.004649	0.39138	0.016545
lh_S_circular_insula_ant_thickness	4.80303	0.171211	0.007825	0.590256	0.037
lh_S_circular_insula_inf_thickness	0.000429	0.989545	7.04E-07	0.050049	-0.0003
lh_S_circular_insula_sup_thickness	0.380344	0.789018	0.000624	0.094441	0.006435
lh_S_collat_transv_post_thickness	0.048515	0.914793	7.97E-05	0.055558	-0.00336
lh_S_front_inf_thickness	2.554187	0.284908	0.004177	0.358005	0.01657
lh_S_front_middle_thickness	1.691582	0.398345	0.00277	0.254736	0.015559
lh_S_front_sup_thickness	9.440418	0.035794	0.015265	0.866007	0.031
lh_S_intrapariet.P_trans_thickness	0.774089	0.637556	0.001269	0.141998	0.008379
lh_S_oc.temp_med.Lingual_thickness	2.79468	0.271231	0.004568	0.385717	0.020066
lh_S_oc_middle.Lunatus_thickness	1.229989	0.522698	0.002016	0.198003	0.013854
lh_S_oc_sup.transversal_thickness	0.019093	0.946592	3.14E-05	0.052183	0.001584
lh_S_occipital_ant_thickness	0.023022	0.946592	3.78E-05	0.052633	0.002035
lh_S_orbital.H_Shaped_thickness	3.19011	0.255388	0.005211	0.429966	0.025294
lh_S_orbital_med.olfact_thickness	4.689018	0.173701	0.007641	0.580074	0.03
lh_S_parieto_occipital_thickness	0.097465	0.898052	0.00016	0.061203	0.003506
lh_S_postcentral_thickness	14.77566	0.010769	0.023687	0.969801	0.039
lh_S_precentral.inf.part_thickness	5.153531	0.156523	0.008391	0.620436	0.026
lh_S_precentral.sup.part_thickness	13.46014	0.010769	0.021624	0.955721	0.045

lh_S_suborbital_thickness	2.382573	0.298697	0.003897	0.337898	0.030549
lh_S_temporal_inf_thickness	0.187105	0.847666	0.000307	0.071627	0.00554
lh_S_temporal_sup_thickness	0.004186	0.980276	6.87E-06	0.050478	0.000591
rh_G.S_cingul.Ant_thickness	0.205516	0.847666	0.000337	0.073782	0.005649
rh_G.S_cingul.Mid.Ant_thickness	5.242276	0.156523	0.008535	0.627809	0.028
rh_G.S_cingul.Mid.Post_thickness	5.301827	0.156523	0.008631	0.632695	0.026
rh_G.S_frontomargin_thickness	7.454272	0.066893	0.012092	0.778157	-0.039
rh_G.S_paracentral_thickness	10.82056	0.019995	0.017458	0.907299	0.046
rh_G.S_subcentral_thickness	0.055938	0.912891	9.18E-05	0.056412	-0.00323
rh_G.S_transv_frontopol_thickness	0.202916	0.847666	0.000333	0.073477	-0.00652
rh_G_cingul.Post.dorsal_thickness	2.325984	0.300742	0.003805	0.331213	0.021338
rh_G_cuneus_thickness	7.968967	0.055529	0.012916	0.804698	0.029
rh_G_front_inf.Opercular_thickness	6.493542	0.104253	0.01055	0.72048	0.033
rh_G_front_inf.Triangul_thickness	0.200575	0.847666	0.000329	0.073203	0.006155
rh_G_front_middle_thickness	3.559816	0.235774	0.005811	0.469694	0.02103
rh_G_front_sup_thickness	10.88445	0.019995	0.017559	0.908896	0.039
rh_G_insular_short_thickness	1.09906	0.544704	0.001801	0.181875	-0.0224
rh_G_oc.temp_med.Lingual_thickness	0.828774	0.621478	0.001359	0.148682	0.010006
rh_G_oc.temp_med.Parahip_thickness	2.81064	0.271231	0.004594	0.387536	0.03232
rh_G_occipital_middle_thickness	0.440654	0.753916	0.000723	0.101644	0.008098
rh_G_occipital_sup_thickness	3.05992	0.268373	0.004999	0.415589	0.025138
rh_G_orbital_thickness	2.147024	0.317647	0.003513	0.309904	0.018413
rh_G_pariet_inf.Angular_thickness	2.369615	0.298697	0.003876	0.33637	0.018902
rh_G_pariet_inf.Supramar_thickness	2.800209	0.271231	0.004577	0.386348	0.020038
rh_G_parietal_sup_thickness	0.066901	0.908556	0.00011	0.057674	0.003043
rh_G_postcentral_thickness	3.536514	0.235774	0.005774	0.467239	0.024271
rh_G_precentral_thickness	12.43764	0.012781	0.020014	0.940759	0.053
rh_G_precuneus_thickness	0.691584	0.646093	0.001134	0.131943	0.009905
rh_G_rectus_thickness	2.512593	0.284908	0.004109	0.353156	0.02492

rh_G_temp_sup.G_T_transv_thickness	0.066933	0.908556	0.00011	0.057678	-0.00478
rh_G_temp_sup.Lateral_thickness	0.249824	0.847666	0.00041	0.078984	0.007706
rh_G_temp_sup.Plan_polar_thickness	0.539906	0.706639	0.000886	0.11357	0.015871
rh_G_temp_sup.Plan_tempo_thickness	1.789251	0.379841	0.002929	0.266659	0.020768
rh_G_temporal_inf_thickness	0.760031	0.637556	0.001246	0.140282	0.012153
rh_G_temporal_middle_thickness	0.009729	0.964122	1.6E-05	0.051112	-0.00142
rh_Lat_Fis.post_thickness	3.894994	0.212448	0.006355	0.50423	0.024
rh_Pole_occipital_thickness	0.190511	0.847666	0.000313	0.072026	0.004819
rh_Pole_temporal_thickness	0.739848	0.638774	0.001213	0.137821	0.014814
rh_S_calcarine_thickness	3.441955	0.238264	0.00562	0.45721	0.020828
rh_S_central_thickness	3.57808	0.235774	0.005841	0.471613	0.018672
rh_S_cingul.Marginalis_thickness	4.069184	0.207687	0.006637	0.521598	0.023
rh_S_circular_insula_ant_thickness	0.054224	0.912891	8.9E-05	0.056215	0.004076
rh_S_circular_insula_sup_thickness	1.227725	0.522698	0.002012	0.197724	0.011695
rh_S_front_inf_thickness	0.164949	0.859783	0.000271	0.069041	0.004047
rh_S_front_middle_thickness	2.739487	0.271231	0.004478	0.379408	0.017524
rh_S_front_sup_thickness	13.3149	0.010769	0.021396	0.953835	0.038
rh_S_intrapariet.P_trans_thickness	1.129936	0.543838	0.001852	0.185677	0.010179
rh_S_oc.temp_med.Lingual_thickness	2.624123	0.284567	0.00429	0.366122	0.02062
rh_S_oc_middle.Lunatus_thickness	0.224591	0.847666	0.000369	0.076018	0.006354
rh_S_oc_sup.transversal_thickness	0.351316	0.801994	0.000577	0.090987	0.006847
rh_S_orbital.H_Shaped_thickness	0.081523	0.908556	0.000134	0.059361	0.004219
rh_S_orbital_med.olfact_thickness	4.162949	0.205111	0.006789	0.530779	0.032
rh_S_parieto_occipital_thickness	2.191053	0.314889	0.003585	0.315169	0.01718
rh_S_postcentral_thickness	4.434466	0.191718	0.007229	0.556694	0.022
rh_S_precentral.inf.part_thickness	9.066643	0.038293	0.014669	0.852305	0.033
rh_S_suborbital_thickness	0.106282	0.895019	0.000174	0.062223	0.009486
rh_S_temporal_inf_thickness	2.513723	0.284908	0.004111	0.353288	-0.02124
rh_S_temporal_sup_thickness	0.108183	0.895019	0.000178	0.062443	-0.00312

rh_S_temporal_transverse_thickness	5.831423	0.13939	0.009485	0.674033	-0.055

Table S17: cortical thickness differences FND-motor vs HCs (with inclusion of Euler as covariate)

Dependent Variable	Mean Difference (I-J)	Sig (FDR)
lh_G.S_occipital_inf_area	-2.7E-05	0.840387
lh_G.S_paracentral_area	-6.4E-05	0.602776
lh_G.S_cingul.Ant_area	-5E-05	0.765513
lh_G.S_cingul.Mid.Ant_area	-3.3E-05	0.786769
lh_G_cingul.Post.ventral_area	-3.658E-5*	0.364409
lh_G_cuneus_area	9.85E-05	0.644357
lh_G_front_inf.Triangul_area	2.4E-05	0.818981
lh_G_front_middle_area	1.4E-05	0.946429
lh_G_front_sup_area	-0.00028	0.482873
lh_G_insular_short_area	2.51E-05	0.735064
lh_G_occipital_middle_area	7.72E-05	0.735064
lh_G_occipital_sup_area	0.000104	0.402069
lh_G_oc.temp_lat.fusifor_area	-7.4E-05	0.735064
lh_G_oc.temp_med.Lingual_area	-6E-05	0.786769
lh_G_pariet_inf.Angular_area	4.91E-05	0.786769
lh_G_pariet_inf.Supramar_area	-7E-05	0.786769
lh_G_parietal_sup_area	-0.00012	0.735064
lh_G_precentral_area	-1.4E-05	0.912499
lh_G_precuneus_area	-0.00018	0.402069
lh_G_temp_sup.G_T_transv_area	-3.8E-05	0.609495
lh_G_temp_sup.Plan_tempo_area	-0.0001	0.412002
lh_G_temporal_inf_area	0.000205	0.402069
lh_G_temporal_middle_area	7.96E-05	0.735064
lh_Lat_Fis.post_area	-6.9E-05	0.412002

lh_Pole_occipital_area	-2E-05	0.867631
lh_S_calcarine_area	-0.00011	0.735064
lh_S_central_area	6.85E-05	0.735064
lh_S_circular_insula_ant_area	1.18E-05	0.786769
lh_S_circular_insula_inf_area	-8.814E-5*	0.402069
lh_S_circular_insula_sup_area	.000*	0.282559
lh_S_collat_transv_post_area	-1.8E-05	0.786769
lh_S_front_inf_area	3.62E-05	0.818981
lh_S_front_middle_area	2.41E-05	0.840401
lh_S_front_sup_area	6.96E-05	0.786769
lh_S_intrapariet.P_trans_area	-9.7E-05	0.735064
lh_S_oc_middle.Lunatus_area	0.000106	0.516989
lh_S_oc_sup.transversal_area	6.33E-05	0.735064
lh_S_oc.temp_med.Lingual_area	5.78E-06	0.946429
lh_S_pericallosal_area	3.21E-05	0.786769
lh_S_postcentral_area	0.000181	0.482873
lh_S_precentral.inf.part_area	-0.00011	0.516989
lh_S_subparietal_area	-2.9E-05	0.786769
lh_S_temporal_inf_area	2.92E-05	0.818981
lh_S_temporal_sup_area	0.000341	0.402069
lh_S_temporal_transverse_area	3.85E-05	0.595346
rh_G.S_occipital_inf_area	-1.1E-05	0.906713
rh_G.S_paracentral_area	-9.059E-5*	0.402069
rh_G.S_subcentral_area	2.11E-05	0.818981
rh_G.S_cingul.Mid.Ant_area	-1.8E-05	0.818981
rh_G.S_cingul.Mid.Post_area	-9.760E-5*	0.402069
rh_G_cingul.Post.ventral_area	-4.574E-5*	0.282559

rh_G_cuneus_area	.000*	0.402069
rh_G_front_inf.Orbital_area	-1.5E-05	0.786769
rh_G_front_middle_area	-2.2E-05	0.924981
rh_G_front_sup_area	.000*	0.402069
rh_G_occipital_middle_area	-1.2E-05	0.934493
rh_G_occipital_sup_area	-3.9E-06	0.951603
rh_G_oc.temp_med.Lingual_area	-0.00017	0.509555
rh_G_pariet_inf.Angular_area	8.19E-05	0.786769
rh_G_pariet_inf.Supramar_area	0.000175	0.402069
rh_G_parietal_sup_area	0.000175	0.412002
rh_G_postcentral_area	7.63E-05	0.644357
rh_G_precentral_area	.000*	0.402069
rh_G_precuneus_area	-9.1E-05	0.735064
rh_G_temp_sup.Lateral_area	-3E-05	0.786769
rh_G_temp_sup.Plan_tempo_area	1.84E-05	0.787533
rh_G_temporal_inf_area	0.000161	0.429146
rh_G_temporal_middle_area	-7.3E-05	0.735064
rh_Lat_Fis.post_area	-2.9E-05	0.760808
rh_Pole_occipital_area	-8.7E-05	0.760808
rh_S_calcarine_area	-0.00022	0.402069
rh_S_central_area	9.18E-05	0.707775
rh_S_circular_insula_ant_area	1.81E-05	0.786769
rh_S_circular_insula_inf_area	-5.6E-05	0.459088
rh_S_circular_insula_sup_area	-2.4E-05	0.786769
rh_S_collat_transv_post_area	-3.2E-05	0.735064
rh_S_front_inf_area	0.000102	0.735064
rh_S_front_middle_area	6.25E-05	0.786769
rh_S_front_sup_area	-8.9E-05	0.735064

rh_S_intrapariet.P_trans_area	0.000254	0.402069
rh_S_oc_middle.Lunatus_area	5.95E-05	0.735064
rh_S_oc_sup.transversal_area	-3.7E-05	0.786769
rh_S_orbital_lateral_area	2.83E-05	0.735064
rh_S_postcentral_area	.000*	0.284808
rh_S_subparietal_area	1.82E-05	0.867631
rh_S_temporal_inf_area	6.41E-05	0.735064
rh_S_temporal_sup_area	-0.00017	0.735064

Table S18: relative surface area differences in FND-motor vs HCs (with inclusion of Euler as covariate)

Dependent Variable	F	Sig.	Partial Eta Squared	Observed Power	Mean difference (I-J)
lh_G.S_frontomargin_thickness	0.14005	0.819076	0.00037	0.06611	0.006538
lh_G.S_cingul.Ant_thickness	0.123	0.819076	0.000325	0.064134	0.005617
lh_G_cuneus_thickness	7.011067	0.123785	0.01821	0.752112	.037*
lh_G_front_middle_thickness	0.25524	0.772554	0.000675	0.079564	0.007466
lh_G_occipital_sup_thickness	0.272462	0.772554	0.00072	0.081589	-0.00922
lh_G_oc.temp_med.Lingual_thickness	1.963446	0.633835	0.005167	0.287351	0.018711
lh_G_oc.temp_med.Parahip_thickness	1.036148	0.63996	0.002734	0.173886	-0.02674
lh_G_parietal_sup_thickness	0.264149	0.772554	0.000698	0.080611	-0.00785
lh_G_temp_sup.Lateral_thickness	0.254049	0.772554	0.000672	0.079424	0.01097
lh_G_temp_sup.Plan_polar_thickness	0.000746	0.978218	1.97E-06	0.050085	-0.00071
lh_Pole_occipital_thickness	1.357817	0.63996	0.003579	0.213429	-0.01907
lh_S_calcarine_thickness	1.59988	0.63996	0.004215	0.243128	0.015963
lh_S_cingul.Marginalis_thickness	0.349278	0.772554	0.000923	0.090665	0.008423
lh_S_collat_transv_post_thickness	1.745288	0.633835	0.004596	0.260887	0.026138
lh_S_front_inf_thickness	0.152262	0.819076	0.000403	0.067528	0.005154
lh_S_front_middle_thickness	1.16074	0.63996	0.003061	0.189195	0.016401
lh_S_front_sup_thickness	4.507624	0.378327	0.011784	0.562698	.028*
lh_S_intrapariet.P_trans_thickness	0.003278	0.976568	8.67E-06	0.050374	0.00071
lh_S_oc_middle.Lunatus_thickness	0.361392	0.772554	0.000955	0.092102	0.010003
lh_S_oc_sup.transversal_thickness	0.270393	0.772554	0.000715	0.081346	0.007704
lh_S_orbital_lateral_thickness	0.289785	0.772554	0.000766	0.08363	-0.01315
lh_S_orbital.H_Shaped_thickness	0.288964	0.772554	0.000764	0.083533	-0.00973
lh_S_postcentral_thickness	0.018796	0.933455	4.97E-05	0.052145	0.001679
lh_S_suborbital_thickness	2.524077	0.62126	0.006633	0.353928	0.041372

rh_G.S_frontomargin_thickness	1.441633	0.63996	0.003799	0.223726	-0.02226
rh_G.S_transv_frontopol_thickness	1.800131	0.633835	0.00474	0.267562	0.026295
rh_G.S_cingul.Ant_thickness	2.617515	0.62126	0.006877	0.364772	-0.02625
rh_G_cuneus_thickness	11.57538	0.017722	0.029713	0.924163	.045*
rh_G_occipital_sup_thickness	0.254939	0.772554	0.000674	0.079528	-0.0089
rh_G_oc.temp_med.Lingual_thickness	1.901995	0.633835	0.005007	0.279922	0.019345
rh_G_parietal_sup_thickness	2.893191	0.62126	0.007596	0.396269	-0.02587
rh_G_temp_sup.G_T_transv_thickness	0.094242	0.834923	0.000249	0.060809	0.007282
rh_G_temp_sup.Lateral_thickness	2.106653	0.633835	0.005542	0.304576	0.028433
rh_Pole_occipital_thickness	0.393201	0.772554	0.001039	0.095883	-0.0089
rh_S_cingul.Marginalis_thickness	1.014317	0.63996	0.002676	0.171206	-0.01516
rh_S_front_middle_thickness	0.562115	0.772554	0.001485	0.116119	-0.01081
rh_S_front_sup_thickness	1.270789	0.63996	0.003351	0.202728	0.015671
rh_S_intrapariet.P_trans_thickness	0.960847	0.63996	0.002535	0.164648	-0.01227
rh_S_oc_sup.transversal_thickness	0.055682	0.873113	0.000147	0.05637	0.003367
rh_S_orbital_lateral_thickness	11.41215	0.017722	0.029306	0.920675	-.067*
rh_S_orbital_med.olfact_thickness	0.150337	0.819076	0.000398	0.067304	-0.00758
rh_S_parieto_occipital_thickness	0.99602	0.63996	0.002628	0.168961	0.014248
rh_S_postcentral_thickness	0.933687	0.63996	0.002464	0.161319	-0.01227
rh_S_precentral.sup.part_thickness	3.995498	0.407771	0.01046	0.513532	.035*

Table S19: cortical thickness differences in FND-seiz vs HCs (with inclusion of Euler as covariate)

	F	Sig.	Partial Eta Squared	Observed Power[a]	Mean difference (I-J)
lh_G_cingul.Post.dorsal_thickness	0.038882	0.925129	0.000114	0.054441	-0.00366
lh_G_cuneus_thickness	4.086066	0.439999	0.011807	0.522269	.028[*]
lh_G_front_inf.Opercular_thickness	0.12245	0.925129	0.000358	0.064062	0.00604
lh_G_front_inf.Orbital_thickness	0.025217	0.93561	7.37E-05	0.052877	0.003582
lh_G_front_inf.Triangul_thickness	0.065862	0.925129	0.000193	0.057536	0.004634
lh_G_front_middle_thickness	0.042785	0.925129	0.000125	0.054888	-0.00328
lh_G_front_sup_thickness	0.040319	0.925129	0.000118	0.054605	0.003199
lh_G_Ins_lg.S_cent_ins_thickness	3.341255	0.439999	0.009675	0.445519	-0.05099
lh_G_occipital_middle_thickness	1.995744	0.534713	0.005802	0.291118	-0.02212
lh_G_occipital_sup_thickness	0.051962	0.925129	0.000152	0.05594	-0.00426
lh_G_orbital_thickness	1.573977	0.586322	0.004581	0.239854	-0.02214
lh_G_pariet_inf.Angular_thickness	3.395442	0.439999	0.009831	0.451338	-0.03066
lh_G_pariet_inf.Supramar_thickness	0.941924	0.703598	0.002747	0.162267	-0.0164
lh_G_parietal_sup_thickness	2.774318	0.439999	0.008047	0.382612	-0.02611
lh_G_postcentral_thickness	0.00255	0.981321	7.46E-06	0.050291	0.000875
lh_G_precentral_thickness	2.683153	0.443449	0.007784	0.372175	0.033518
lh_G.S_cingul.Ant_thickness	0.08234	0.925129	0.000241	0.059432	-0.00466
lh_G.S_cingul.Mid.Ant_thickness	0.044164	0.925129	0.000129	0.055046	0.003676
lh_G.S_cingul.Mid.Post_thickness	0.047327	0.925129	0.000138	0.055408	0.003285
lh_G.S_frontomargin_thickness	0.221287	0.922077	0.000647	0.075567	-0.00887
lh_G.S_occipital_inf_thickness	1.045783	0.698887	0.003049	0.175	-0.02008
lh_G.S_paracentral_thickness	0.008641	0.968567	2.53E-05	0.050985	-0.0018
lh_Lat_Fis.post_thickness	1.20586	0.653586	0.003514	0.194664	-0.01838
lh_S_central_thickness	0.159714	0.925129	0.000467	0.068385	0.005239

lh_S_cingul.Marginalis_thickness	0.01508	0.954797	4.41E-05	0.05172	-0.00199
lh_S_circular_insula_ant_thickness	8.315611	0.126777	0.023737	0.820065	-.068*
lh_S_circular_insula_inf_thickness	0.540085	0.752214	0.001577	0.113431	-0.0136
lh_S_circular_insula_sup_thickness	0.0003	0.989624	8.78E-07	0.050034	-0.00024
lh_S_front_inf_thickness	0.310114	0.862225	0.000906	0.08601	-0.00782
lh_S_front_middle_thickness	0.847631	0.716185	0.002472	0.150735	0.015297
lh_S_front_sup_thickness	0.100164	0.925129	0.000293	0.061487	-0.00456
lh_S_intrapariet.P_trans_thickness	0.775585	0.718736	0.002263	0.141948	-0.01163
lh_S_oc_middle.Lunatus_thickness	0.000169	0.989624	4.95E-07	0.050019	-0.00023
lh_S_oc_sup.transversal_thickness	0.733466	0.728671	0.00214	0.136823	0.012821
lh_S_orbital_lateral_thickness	0.378104	0.829561	0.001104	0.094063	-0.01588
lh_S_orbital_med.olfact_thickness	5.189159	0.303481	0.014946	0.622343	-.043*
lh_S_orbital.H_Shaped_thickness	9.82289	0.126777	0.02792	0.878066	-.063*
lh_S_parieto_occipital_thickness	0.105918	0.925129	0.00031	0.062151	0.004955
lh_S_postcentral_thickness	5.84008	0.245519	0.01679	0.673604	-.034*
lh_S_precentral.inf.part_thickness	0.031478	0.930889	9.2E-05	0.053593	-0.00275
lh_S_precentral.sup.part_thickness	0.624045	0.752214	0.001821	0.123556	-0.01371
lh_S_suborbital_thickness	0.18187	0.925129	0.000532	0.070963	0.010961
lh_S_temporal_sup_thickness	0.589762	0.752214	0.001721	0.119415	-0.00939
rh_G_cuneus_thickness	3.067838	0.439999	0.008891	0.415629	0.023313
rh_G_front_inf.Opercular_thickness	1.109803	0.68336	0.003235	0.182861	0.018488
rh_G_front_inf.Triangul_thickness	0.174423	0.925129	0.00051	0.070096	0.007782
rh_G_front_middle_thickness	0.110743	0.925129	0.000324	0.062708	-0.00528
rh_G_front_sup_thickness	0.831844	0.716185	0.002426	0.148807	-0.01492
rh_G_occipital_middle_thickness	0.065905	0.925129	0.000193	0.057541	0.004257
rh_G_occipital_sup_thickness	2.344987	0.466158	0.00681	0.332778	-0.02945
rh_G_orbital_thickness	0.493771	0.770671	0.001442	0.10787	-0.01208

rh_G_pariet_inf.Angular_thickness	5.948554	0.245519	0.017096	0.681595	-.041*
rh_G_pariet_inf.Supramar_thickness	1.697112	0.586322	0.004938	0.254901	-0.02079
rh_G_parietal_sup_thickness	2.408413	0.466158	0.006993	0.340244	-0.02545
rh_G_postcentral_thickness	0.612695	0.752214	0.001788	0.122184	-0.01447
rh_G_precentral_thickness	0.806133	0.716185	0.002352	0.145671	-0.01905
rh_G_precuneus_thickness	0.541911	0.752214	0.001582	0.113651	-0.01221
rh_G_temp_sup.G_T_transv_thickness	3.711981	0.439999	0.010737	0.484607	0.049436
rh_G_temp_sup.Lateral_thickness	1.559297	0.586322	0.004539	0.238056	0.025175
rh_G_temp_sup.Plan_tempo_thickness	0.234462	0.922077	0.000685	0.07711	0.009999
rh_G_temporal_middle_thickness	0.880025	0.716185	0.002567	0.154693	-0.01735
rh_G.S_cingul.Ant_thickness	6.234586	0.245519	0.017903	0.701926	-.042*
rh_G.S_cingul.Mid.Ant_thickness	1.313976	0.638207	0.003827	0.207952	-0.01766
rh_G.S_cingul.Mid.Post_thickness	2.816149	0.439999	0.008167	0.387373	-0.02385
rh_G.S_frontomargin_thickness	4.710991	0.348735	0.013588	0.581006	.045*
rh_G.S_occipital_inf_thickness	0.085862	0.925129	0.000251	0.059837	-0.00671
rh_G.S_paracentral_thickness	2.805786	0.439999	0.008137	0.386195	-0.03079
rh_G.S_subcentral_thickness	1.372579	0.629681	0.003997	0.215153	0.020901
rh_G.S_transv_frontopol_thickness	3.252658	0.439999	0.009421	0.435929	0.035182
rh_Lat_Fis.post_thickness	1.636315	0.586322	0.004762	0.247478	-0.02048
rh_Pole_occipital_thickness	1.238084	0.653586	0.003607	0.198625	-0.01729
rh_S_calcarine_thickness	0.10333	0.925129	0.000302	0.061852	0.00489
rh_S_central_thickness	0.459644	0.781735	0.001342	0.103783	0.00913
rh_S_cingul.Marginalis_thickness	8.617191	0.126777	0.024577	0.833301	-.045*
rh_S_circular_insula_ant_thickness	2.994406	0.439999	0.00868	0.407456	-0.04328
rh_S_circular_insula_sup_thickness	0.559761	0.752214	0.001634	0.115799	-0.01061
rh_S_front_inf_thickness	0.363509	0.829561	0.001062	0.09233	-0.00861
rh_S_front_middle_thickness	3.052801	0.439999	0.008847	0.41396	-0.02598
rh_S_front_sup_thickness	2.49372	0.466158	0.007239	0.350232	-0.02352

rh_S_intrapariet.P_trans_thickness	1.903038	0.54808	0.005534	0.279925	-0.01838
rh_S_oc_middle.Lunatus_thickness	1.444699	0.616156	0.004206	0.224007	-0.02154
rh_S_oc_sup.transversal_thickness	0.00361	0.981321	1.06E-05	0.050411	0.000943
rh_S_occipital_ant_thickness	0.070884	0.925129	0.000207	0.058113	-0.00491
rh_S_orbital_lateral_thickness	3.473635	0.439999	0.010055	0.459672	-0.03965
rh_S_orbital_med.olfact_thickness	2.225925	0.478216	0.006466	0.318675	-0.03068
rh_S_orbital.H_Shaped_thickness	0.581406	0.752214	0.001697	0.118408	0.015056
rh_S_parieto_occipital_thickness	0.093064	0.925129	0.000272	0.060667	-0.00484
rh_S_postcentral_thickness	2.327069	0.466158	0.006758	0.330662	-0.02081
rh_S_precentral.inf.part_thickness	1.75027	0.585936	0.005092	0.261379	-0.02004
rh_S_precentral.sup.part_thickness	0.976203	0.703598	0.002846	0.166467	-0.01931
rh_S_temporal_sup_thickness	0.967503	0.703598	0.002821	0.1654	-0.01254

Table S20: cortical thickness differences in FND-seiz vs FND-motor (with inclusion of Euler as covariate)

	F	Sig.	Partial Eta Squared	Observed Power	Mean Difference (I-J)
lh_G.S_frontomargin_area.1	0.981276	0.666674	0.002861	0.167088	7.44E-06
lh_G.S_paracentral_area.1	0.228097	0.796834	0.000667	0.076364	-3.9E-06
lh_G.S_subcentral_area.1	0.118431	0.823984	0.000346	0.063597	3.05E-06
lh_G.S_cingul.Ant_area.1	0.448473	0.796834	0.00131	0.102448	-8.1E-06
lh_G.S_cingul.Mid.Post_area.1	0.148345	0.81929	0.000434	0.067064	3.22E-06
lh_G_cuneus_area.1	0.699902	0.762034	0.002042	0.132745	1.27E-05
lh_G_front_inf.Opercular_area.1	2.491855	0.399826	0.007233	0.350014	1.51E-05
lh_G_front_middle_area.1	0.209947	0.796834	0.000614	0.07424	1.26E-05
lh_G_front_sup_area.1	0.083745	0.855225	0.000245	0.059593	9.95E-06
lh_G_occipital_sup_area.1	2.970799	0.384199	0.008612	0.404816	1.79E-05
lh_G_orbital_area.1	0.190208	0.796834	0.000556	0.071935	-4.9E-06
lh_Pole_occipital_area.1	1.091709	0.634604	0.003182	0.180639	-1.3E-05
lh_S_cingul.Marginalis_area.1	3.036961	0.384199	0.008802	0.4122	1.19E-05
lh_S_circular_insula_sup_area.1	0.511003	0.796269	0.001492	0.109937	-6.1E-06
lh_S_front_inf_area.1	2.482023	0.399826	0.007205	0.348866	2.69E-05
lh_S_oc.temp_med.Lingual_area.1	1.50725	0.535871	0.004388	0.23168	1.52E-05
lh_S_orbital.H_Shaped_area.1	0.567773	0.777865	0.001657	0.116764	6.78E-06
lh_S_temporal_sup_area.1	2.934463	0.384199	0.008507	0.40074	5.54E-05
rh_G.S_subcentral_area.1	1.207974	0.603408	0.00352	0.194924	1.02E-05
rh_G.S_cingul.Ant_area.1	0.202593	0.796834	0.000592	0.073381	-6.9E-06
rh_G.S_cingul.Mid.Ant_area.1	2.585881	0.399826	0.007504	0.360949	-1.4E-05
rh_G.S_cingul.Mid.Post_area.1	0.013249	0.938709	3.87E-05	0.051511	9.62E-07
rh_G_cuneus_area.1	2.212561	0.427219	0.006428	0.317085	-2.4E-05
rh_G_front_middle_area.1	0.220761	0.796834	0.000645	0.075505	-1.2E-05
rh_G_front_sup_area.1	0.266911	0.796834	0.00078	0.080919	1.78E-05

rh_G_oc.temp_med.Lingual_area.1	2.10475	0.436241	0.006117	0.304213	-3.2E-05
rh_G_orbital_area.1	1.425283	0.535871	0.00415	0.221624	1.61E-05
rh_G_rectus_area.1	1.775812	0.480586	0.005166	0.264488	6.1E-06
rh_G_temporal_inf_area.1	0.048139	0.883462	0.000141	0.055502	3.54E-06
rh_G_temporal_middle_area.1	0.187892	0.796834	0.000549	0.071665	-7.2E-06
rh_Lat_Fis.post_area.1	4.566832	0.384199	0.013177	0.567928	1.460E-5*
rh_Pole_occipital_area.1	3.978736	0.384199	0.0115	0.511651	-4.387E-5*
rh_Pole_temporal_area.1	0.65442	0.76424	0.00191	0.127231	-7.6E-06
rh_S_calcarine_area.1	3.334484	0.384199	0.009656	0.444789	-4E-05
rh_S_circular_insula_inf_area.1	4.024922	0.384199	0.011632	0.516239	1.256E-5*
rh_S_circular_insula_sup_area.1	3.950869	0.384199	0.01142	0.508869	1.464E-5*
rh_S_temporal_sup_area.1	0.459463	0.796834	0.001342	0.103762	-2.2E-05
lh_G.S_occipital_inf_area.1	2.870021	0.384199	0.008322	0.393478	-2.3E-05
lh_G_cingul.Post.ventral_area.1	4.54704	0.384199	0.013121	0.56611	-5.359E-6*
lh_G_occipital_middle_area.1	0.367228	0.796834	0.001073	0.092772	1.04E-05
lh_G_oc.temp_med.Lingual_area.1	0.34861	0.796834	0.001018	0.090564	-1.3E-05
lh_G_pariet_inf.Supramar_area.1	1.45136	0.535871	0.004226	0.224825	-2.6E-05
lh_G_temp_sup.G_T_transv_area.1	0.693378	0.762034	0.002023	0.131954	-4E-06
lh_G_temp_sup.Lateral_area.1	2.276799	0.427219	0.006613	0.324714	1.54E-05
lh_G_temp_sup.Plan_tempo_area.1	0.052013	0.883462	0.000152	0.055946	-2.2E-06
lh_Lat_Fis.post_area.1	0.210219	0.796834	0.000614	0.074272	3E-06
lh_S_calcarine_area.1	1.875006	0.480586	0.005453	0.276531	-3.2E-05
lh_S_circular_insula_inf_area.1	1.755805	0.480586	0.005108	0.262053	9.16E-06
lh_S_collat_transv_post_area.1	0.352393	0.796834	0.001029	0.091012	-2.9E-06
lh_S_oc_middle.Lunatus_area.1	0.589605	0.777865	0.001721	0.119397	-9.6E-06
rh_G.S_occipital_inf_area.1	0.002341	0.96144	6.84E-06	0.050267	-4.8E-07
rh_G_cingul.Post.ventral_area.1	3.447943	0.384199	0.009981	0.456942	-5E-06

rh_G_occipital_middle_area.1	0.420849	0.796834	0.001229	0.099151	-1.2E-05
rh_G_occipital_sup_area.1	0.183901	0.796834	0.000537	0.0712	4.65E-06
rh_G_pariet_inf.Supramar_area.1	4.440018	0.384199	0.012816	0.556189	3.720E-5*
rh_G_temp_sup.G_T_transv_area.1	5.444496	0.384199	0.01567	0.643134	8.545E-6*
rh_G_temp_sup.Lateral_area.1	0.838611	0.720879	0.002446	0.149633	9.17E-06
rh_G_temp_sup.Plan_tempo_area.1	3.858633	0.384199	0.011157	0.499589	1.28E-05
rh_S_collat_transv_post_area.1	0.038175	0.888185	0.000112	0.05436	-1.1E-06
rh_S_oc_middle.Lunatus_area.1	0.126142	0.823984	0.000369	0.06449	-4.1E-06
rh_S_oc_sup.transversal_area.1	0.0024	0.96144	7.02E-06	0.050273	5.37E-07
rh_S_temporal_transverse_area.1	2.838343	0.384199	0.008231	0.389892	6E-06

Table S21: relative cortical surface area differences in FND-seiz vs FND-motor (with inclusion of Euler as covariate)

Dependent Variable	Mean Difference (I-J)	Sig.[b]
lh_G_cingul.Post.dorsal_thickness	-0.00403	0.857196
lh_G_cuneus_thickness	0.009017	0.587046
lh_G_front_inf.Opercular_thickness	-.047*	0.034341
lh_G_front_inf.Triangul_thickness	0.004469	0.839428
lh_G_front_middle_thickness	0.004291	0.823748
lh_G_front_sup_thickness	0.028984	0.16532
lh_G_Ins_lg.S_cent_ins_thickness	0.01817	0.591691
lh_G_occipital_middle_thickness	0.033255	0.085226
lh_G_occipital_sup_thickness	0.042883	0.073711
lh_G_orbital_thickness	0.000658	0.974146
lh_G_pariet_inf.Angular_thickness	.055*	0.006436
lh_G_pariet_inf.Supramar_thickness	.041*	0.045121
lh_G_parietal_sup_thickness	.042*	0.025523
lh_G_postcentral_thickness	.046*	0.037369
lh_G_precentral_thickness	0.021604	0.402779
lh_G_rectus_thickness	-0.02412	0.319371
lh_G_subcallosal_thickness	-0.03136	0.461307
lh_G_temp_sup.Lateral_thickness	0.001191	0.965365
lh_G_temp_sup.Plan_polar_thickness	-0.03188	0.334354
lh_G_temp_sup.Plan_tempo_thickness	0.00871	0.743389
lh_G.S_cingul.Ant_thickness	0.034232	0.099656
lh_G.S_cingul.Mid.Post_thickness	0.017078	0.357634
lh_G.S_frontomargin_thickness	0.005023	0.830025

lh_G.S_occipital_inf_thickness	0.0156	0.497496
lh_G.S_paracentral_thickness	0.011597	0.63762
lh_G.S_subcentral_thickness	-0.00316	0.885567
lh_Lat_Fis.post_thickness	0.02814	0.197146
lh_Pole_occipital_thickness	-0.02725	0.188643
lh_S_central_thickness	0.00618	0.692291
lh_S_cingul.Marginalis_thickness	-0.00299	0.88342
lh_S_circular_insula_ant_thickness	-0.01605	0.583005
lh_S_circular_insula_inf_thickness	-0.00597	0.7979
lh_S_circular_insula_sup_thickness	-0.01283	0.450437
lh_S_front_inf_thickness	-.046*	0.007715
lh_S_front_middle_thickness	-0.02615	0.161928
lh_S_front_sup_thickness	-0.00809	0.631737
lh_S_intrapariet.P_trans_thickness	0.014091	0.389034
lh_S_oc_middle.Lunatus_thickness	0.022148	0.287028
lh_S_oc_sup.transversal_thickness	0.022271	0.231399
lh_S_oc.temp_med.Lingual_thickness	0.035689	0.060873
lh_S_occipital_ant_thickness	.061*	0.007482
lh_S_orbital_med.olfact_thickness	-0.02351	0.30145
lh_S_orbital.H_Shaped_thickness	-.050*	0.04059
lh_S_parieto_occipital_thickness	0.02488	0.148598
lh_S_postcentral_thickness	0.022342	0.181125
lh_S_precentral.inf.part_thickness	-0.00846	0.653931
lh_S_precentral.sup.part_thickness	0.023813	0.243012
lh_S_suborbital_thickness	0.061337	0.057279
lh_S_temporal_sup_thickness	-0.01277	0.391683
rh_G_cingul.Post.dorsal_thickness	-0.01784	0.414492
rh_G_cuneus_thickness	0.026113	0.10035

rh_G_front_inf.Opercular_thickness	-0.02324	0.294919
rh_G_front_inf.Triangul_thickness	0.011985	0.597187
rh_G_front_middle_thickness	0.014079	0.478255
rh_G_front_sup_thickness	0.01791	0.386777
rh_G_insular_short_thickness	0.006456	0.852095
rh_G_occipital_middle_thickness	0.012402	0.534979
rh_G_occipital_sup_thickness	0.031197	0.194574
rh_G_orbital_thickness	-0.00598	0.762885
rh_G_pariet_inf.Angular_thickness	.054*	0.012247
rh_G_pariet_inf.Supramar_thickness	0.019616	0.314366
rh_G_parietal_sup_thickness	.045*	0.027007
rh_G_postcentral_thickness	0.043874	0.060132
rh_G_precentral_thickness	0.03153	0.224638
rh_G_precuneus_thickness	-0.00134	0.947127
rh_G_temp_sup.G_T_transv_thickness	0.023664	0.463961
rh_G_temp_sup.Lateral_thickness	0.015856	0.539923
rh_G_temp_sup.Plan_tempo_thickness	0.033357	0.202271
rh_G_temporal_middle_thickness	-0.00238	0.917349
rh_G.S_cingul.Ant_thickness	0.031537	0.126293
rh_G.S_cingul.Mid.Ant_thickness	0.032279	0.106824
rh_G.S_cingul.Mid.Post_thickness	0.005604	0.771159
rh_G.S_paracentral_thickness	0.017881	0.454438
rh_G.S_subcentral_thickness	-0.00262	0.907601
rh_Lat_Fis.post_thickness	0.005015	0.820811
rh_Pole_occipital_thickness	0.017441	0.342906
rh_S_calcarine_thickness	0.014837	0.44077
rh_S_central_thickness	0.008097	0.618899
rh_S_cingul.Marginalis_thickness	-0.0039	0.836065

rh_S_circular_insula_ant_thickness	-0.00133	0.965728
rh_S_circular_insula_inf_thickness	-0.02201	0.404525
rh_S_circular_insula_sup_thickness	-0.02621	0.144352
rh_S_front_inf_thickness	-0.02007	0.270606
rh_S_front_middle_thickness	-0.00459	0.791547
rh_S_front_sup_thickness	-0.00382	0.825865
rh_S_intrapariet.P_trans_thickness	0.001206	0.940015
rh_S_oc_sup.transversal_thickness	0.016408	0.421349
rh_S_oc.temp_med.Lingual_thickness	.049*	0.015525
rh_S_occipital_ant_thickness	0.00595	0.779042
rh_S_orbital_med.olfact_thickness	-0.00117	0.965847
rh_S_orbital.H_Shaped_thickness	-0.00773	0.752412
rh_S_parieto_occipital_thickness	0.012259	0.542252
rh_S_postcentral_thickness	0.027457	0.127428
rh_S_precentral.inf.part_thickness	0.024659	0.206645
rh_S_temporal_sup_thickness	-0.00791	0.61211

Table S22: relative cortical surface area differences in FND-seiz vs FND-motor (with inclusion of Euler as covariate)

Supplementary References

1. Fortin JP, Cullen N, Sheline YI, Taylor WD, Aselcioglu I, Cook PA, et al. Harmonization of cortical thickness measurements across scanners and sites. NeuroImage. 2018 Feb;167:104–20.

2. Johnson WE, Li C, Rabinovic A. Adjusting batch effects in microarray expression data using empirical Bayes methods. Biostatistics. 2007 Jan 1;8(1):118–27.

3. Fortin JP, Parker D, Tunç B, Watanabe T, Elliott MA, Ruparel K, et al. Harmonization of multi-site diffusion tensor imaging data. NeuroImage. 2017 Nov;161:149–70.

4. Jaramillo-Jimenez A, Tovar-Rios DA, Mantilla-Ramos YJ, Ochoa-Gomez JF, Bonanni L, Brønnick K. ComBat models for harmonization of resting-state EEG features in multisite studies. Clin Neurophysiol. 2024 Nov;167:241–53.

5. Radua J, Vieta E, Shinohara R, Kochunov P, Quidé Y, Green MJ, et al. Increased power by harmonizing structural MRI site differences with the ComBat batch adjustment method in ENIGMA. NeuroImage. 2020 Sep;218:116956.
